# DNA-PK driven nucleosome unwrapping enables NHEJ in chromatin

**DOI:** 10.1038/s41467-026-77534-9

**Published:** 2026-09-04

**Authors:** Weifeng Lu, Alex Vogt, Susan P. Lees-Miller, Yuan He

**Affiliations:** 1 Department of Biophysics, Johns Hopkins University, Baltimore, MD USA; 2 Doctoral Program in Cell, Molecular, Developmental Biology, and Biophysics, Johns Hopkins University, Baltimore, MD USA; 3 Department of Molecular Biosciences, Northwestern University, Evanston, IL USA; 4 Interdisciplinary Biological Sciences Program, Northwestern University, Evanston, IL USA; 5 Department of Biochemistry and Molecular Biology, University of Calgary, Calgary, AB Canada; 6 Robson DNA Science Centre, Charbonneau Cancer Institute, University of Calgary, Calgary, AB Canada; 7 Department of Biology, Johns Hopkins University, Baltimore, MD USA

**Keywords:** Cryoelectron microscopy, Cryoelectron microscopy, Non-homologous-end joining, Nucleosomes

## Abstract

DNA double-strand breaks, one of the most cytotoxic forms of DNA damage, are primarily repaired by non-homologous end joining (NHEJ) in human cells. NHEJ is initiated by the Ku70/80 heterodimer (Ku) and DNA-dependent protein kinase catalytic subunit (DNA-PKcs), followed by factors that bridge and ligate DNA ends. Ku and DNA-PKcs require ~28 base pairs of free duplex DNA, which is often unavailable in chromatin with nucleosomes acting as barriers. The role of DNA-PKcs remains unclear, as previous in vitro studies mainly used naked DNA. Here, in vitro ligation assays show that DNA-PKcs promotes NHEJ on nucleosomes with limited DNA accessibility. Cryo-EM structures of nucleosome-bound Ku and DNA-PKcs reveal that Ku encounters nucleosomal barriers and overcomes them with DNA-PKcs. Distinct structural states support a stepwise model of DNA-PK progressive translocating along nucleosomal DNA, highlighting DNA-PKcs function in chromatin-associated NHEJ in vivo.

## Abstract (web summary)

DNA repair proteins work around nucleosomes, which package DNA in cells and limits DNA accessibility for DNA repair factors. Here, by using cryo-EM and biochemical assays, the authors show that DNA-PKcs promotes NHEJ by overcoming histone barriers and moving along nucleosomal DNA.

## Introduction

DNA double-strand breaks (DSBs) are one of the most cytotoxic forms of DNA damage, and their improper repair can lead to genomic instability or apoptosis^1,2^. In human cells, non-homologous end joining (NHEJ) is the predominant pathway that accounts for most DSB repair events^3–5^. NHEJ proceeds through a set of mechanistically distinct steps to join DNA ends. Within seconds upon the formation of a DSB, the DNA ends are recognized and bound by the Ku70/80 (Ku) heterodimer, which subsequently recruits DNA-dependent protein kinase catalytic subunit (DNA-PKcs) to the site of damage^6–9^. Together, Ku and DNA-PKcs form the holoenzyme DNA-PK, a critical protein kinase that phosphorylates multiple NHEJ and DSB repair factors^8,10–12^. The bound DNA ends are then brought into proximity through the formation of a long-range (LR) synaptic complex, mediated by scaffolding proteins including XRCC4, DNA Ligase-IV, XLF and PAXX^13–19^. Autophosphorylation of DNA-PKcs in trans triggers its disassociation from the LR complex, followed by the transition to a short-range (SR) synaptic complex, in which the DNA ends are aligned and ligated by DNA Ligase-IV^14,19–21^.

Although DNA-PKcs is a key component of the NHEJ pathway, whether it is indispensable remains unclear, with various in vivo*/*ex vivo and in vitro studies supporting conflicting conclusions. Loss of DNA-PKcs or inhibition of its kinase activity sensitizes human cells to DSB-inducing agents^22,23^ and blocks end joining in cell extracts^24,25^. In contrast, studies using reconstitution of NHEJ systems in vitro using purified NHEJ factors provide different results: some demonstrating that DNA-PKcs is not essential for formation of a ligation-competent NHEJ complex^14,26^, while others indicating that DNA-PKcs is required for end joining^27^. In addition, mice with catalytically inactive DNA-PKcs mutations are lethal, whereas DNA-PKcs knockout mice are viable^28^. It is well established that chromatinized DNA plays an active role in regulating DNA-dependent processes such as transcription and replication^29–33^, yet the function of DNA-PKcs in a chromatin context remains poorly characterized, especially at the biochemical and structural levels. In human cells, genomic DNA is packaged into chromatin composed of nucleosomes separated by linker regions with an average length of 50 base pairs (bp)^34^. These linker regions are more prone to DSBs, as nucleosomes effectively shield DNA from radiation and oxidative damage^35^. Damage occurring in linker regions can therefore produce nucleosomes with DNA linkers shorter than the footprint of either Ku or both Ku and DNA-PKcs, which combined require approximately 25 bp of free duplex DNA for full assembly and activity^36^. As the first sensors of DSBs in mammalian cells, Ku and DNA-PKcs are recruited immediately upon break formation, and their assembly can facilitate downstream histone modifications and local chromatin reorganization^37^. In addition, some chromatin-remodeling complexes directly related to DNA-damage response have also been shown to slide the histone octamer to the DNA ends, potentially favoring the formation of short DNA linkers^38–40^. Therefore, understanding how Ku and DNA-PKcs engage chromatinized DNA ends under such constrained conditions, where only minimal free duplex DNA is available, is critical for defining the molecular basis of NHEJ within chromatin.

Here, we explore the role of DNA-PKcs in a chromatin context by reconstituting human NHEJ complexes on defined nucleosomal substrates. We examine the activity of the NHEJ complexes on nucleosomal substrates and show that DNA-PKcs promotes effective NHEJ in a chromatin context in vitro. Furthermore, we determine a series of cryo-EM structures of nucleosome-bound Ku and DNA-PK complexes, which capture DNA-PK at various steps of nucleosome translocation. Together, our findings support a stepwise model of DNA-PKcs assisting Ku in initiating NHEJ on chromatinized DNA.

## Results

### Ku encounters nucleosomal barriers during NHEJ

**Fig. 1:**
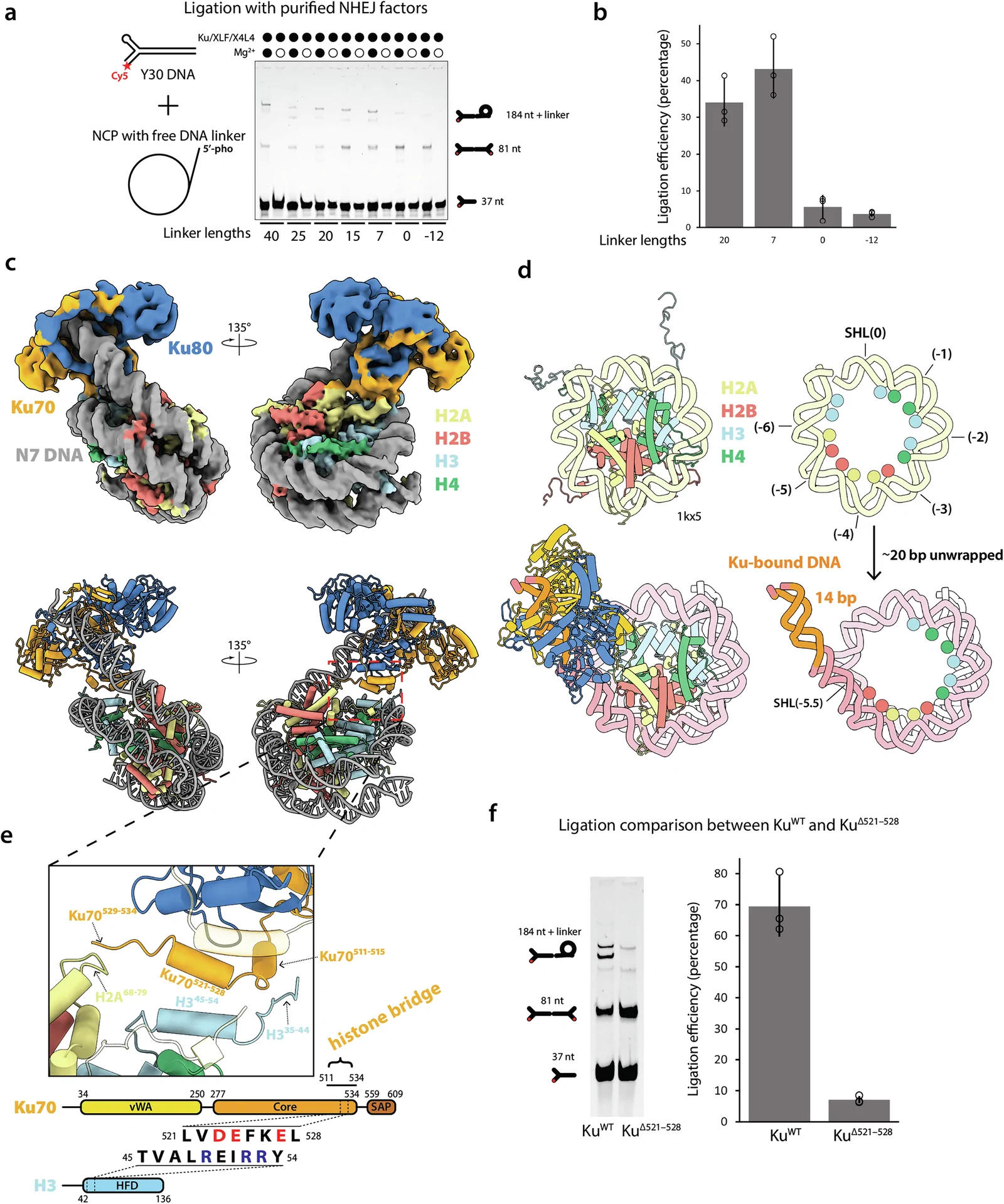
Ku binding promotes nucleosomal DNA unwrapping and direct histone contacts. **a** Denaturing TBE-urea gel analysis showing end joining of blunt-end DNA and nucleosomal substrates by components of the SR complex. Simplified substrate designs are indicated on the left, with defined linker lengths marked below the gel. Three detectable species of the reaction and their corresponding lengths are shown on the right. Source data are provided as a Source Data file. **b** Quantification of the ligation reactions shown in a from three replicates. Ligation efficiency was calculated as the intensity of the Y30-nucleosome ligation product divided by the intensity of the Y30 internal ligation product. Individual replicate values are shown for each condition. Error bars are presented as mean values ±SD. *P* value of each condition from left to right: 0.0014, 0.0221, 0.0152, 0.0093. Source data are provided as a Source Data file. **c** Composite map (top) and structural model (bottom) of the N7-Ku complex. **d** Comparison of the intact nucleosome (top) and the Ku-bound N7 (bottom). Cartoon representations of the intact nucleosome (PDB: 1kx5) and the Ku-bound N7 are shown on the left; histones are represented by colored circles to indicate DNA contact interfaces (right). Super Helical Locations (SHL) are labeled. In the intact nucleosome, DNA beyond SHL (0.5) is omitted for clarity. **e** Close-up view of the Ku70-histone interface and domain architectures of Ku70 and H3. Regions containing residues responsible for the interface are numbered. Sequences of helices showing major interactions are listed below, with positively charged residues in purple and negatively charged residues in red. **f** Denaturing TBE-urea gel analysis showing end joining of blunt-end DNA and nucleosomal substrates using Ku^WT^ and Ku^Δ521-528^. Quantification of three replicates is shown on the right. Ligation efficiency is calculated by dividing the band intensity of Y30-nucleosome ligation by that of the Y30 internal ligation. Individual replicate values are shown for each condition. Error bars are presented as mean values ± SD. *P* value of each condition from left to right: 0.043, 0.00114. Source data are provided as a Source Data file.

In vitro ligation assays have been used previously to assess the ligation activities of various reconstituted NHEJ assemblies under different conditions^14,41–44^. These studies demonstrate that the minimal set of NHEJ factors needed to carry out end joining on free DNA is Ku, XLF, and the XRCC4-DNA LigIV (X4L4) complex. To further examine the extent of these factors’ ability to mediate ligation, we assayed ligation on DNA substrates incorporating a series of nucleosomal substrates that were designed, each consisting of a mononucleosome reconstituted on a Widom 601 sequence^45^ and a ligatable 5′-phosphorylated linker DNA of a defined length (Fig. 1a, and Supplementary Table 1). For detection, the nucleosomal substrates were mixed with a Cy5-labeled Y-shape DNA (Y30). NHEJ factors (XLF, X4L4) were added to the substrates, followed by Mg^2+^ to activate DNA Ligase IV. This assay generated three detectable species: unligated Y30, self-ligated Y30, and Y30 ligated to the nucleosome. In Ku-mediated reactions, substrates with the linker of 0 bp and −12 bp (N0 and N-12; a negative linker length corresponds to an incomplete Widom 601 sequence) showed lower activity for Y30-nucleosome ligation than the Y30 self-ligation, which served as an internal control (Fig. 1a, b), whereas on substrates with linker longer than 7 bp, Ku-mediated nucleosomal ligation shows much higher activity (Fig. 1a, b). This difference in activity suggests that Ku only has limited access to nucleosomes lacking free DNA linkers. Notably, this nucleosome-related reduction in activity resembles observations from in vitro studies of other DNA-repair pathways, such as NER^46^ and BER^47,48^. Our results indicate that nucleosomes can likewise impede NHEJ-mediated ligation by serving as a barrier for Ku, thereby preventing the assembly of the rest of the complex.

Ku requires 14 bp of free DNA duplex to load^49^; therefore, the mechanism of how it engages protein-occluded DNA ends is particularly intriguing. One previous study has suggested that yeast Ku can load onto nucleosomal linkers by peeling ~50 bp DNA away^50^. To structurally characterize how Ku loads onto chromatinized DNA ends, we modified the N7 (nucleosome substrate with free DNA linker of 7 bp) and N0 substrates used in the ligation assays to include one linker of the defined length and biotinylated the opposite end in order to purify the resulting complex using a streptavidin-based pulldown. SDS-PAGE analysis of the N0-Ku and N7-Ku pulldown samples showed that both N0 and N7 substrates were eluted with Ku (Supplementary Fig. 1a). However, further examination by NS-EM revealed a clear difference in assembly efficiency between the two substrates. The N7-Ku sample contained 71% assembled complex, whereas the N0-Ku sample contained only 22% assembled complex (Supplementary Fig. 1b–d). Despite their similar SDS-PAGE profiles, the difference observed by NS-EM suggests that Ku does not stably engage the N0 substrate. As a result, N7 supports more stable Ku binding, resulting in a higher proportion of assembled N7-Ku particles in the NS-EM dataset. This difference in complex formation is consistent with the distinct ligation activities observed for the N0 and N7 substrates.

We then determined the cryo-EM structure of the N7-Ku complex at 4 Å for the nucleosome and 6 Å for the Ku with ~80,000 particles collected in 7095 cryo-EM movies (Fig. 1c, and Supplementary Fig. 2). In the structure, Ku occupies the first 15 bp in the substrate. Comparison between the intact nucleosome (1kx5^51^) revealed that Ku unwraps approximately two turns of nucleosomal DNA from SHL (−7) to SHL (−5.5) when binding the N7 nucleosome (Fig. 1d). In the intact nucleosome, this region interacts with histone H3 and H2A^51^, but in the Ku-bound state, those interfaces are released and replaced by contacts with the C-terminal region of the Ku70 core domain (Fig. 1e). A new interface between Ku and H3 involves Ku residues 511-528, which includes two helices (helices 511-515 and 521-528) with conserved negatively charged residues (Supplementary Fig. 1e), and H3 residues 35-54, which contains positively charged residues that normally engage the DNA entering the intact nucleosome^51^. The exposed DNA-binding region of H2A^51^ (residues 68-79) interacts with the adjacent region in Ku70 (residues 529-534) in the N7-Ku model. No specific function has been assigned to this region (residues 511–539) of Ku70, although the domain to which it belongs participates in the dimerization of Ku70 and Ku80^52^. In the N7-Ku structure, we can find that through this ‘histone-bridge’ region in Ku70, Ku is able to outcompete the histone-DNA interactions by stabilizing the exposed histone interfaces.

To further examine the role of Ku70-histone interactions in nucleosomal DNA engagement, we performed mutagenesis analysis. We generated a Ku70 mutant lacking residues 521-528, which correspond to one of the helices within the Ku70 histone-bridge region. We then compared the enzymatic activity of NHEJ reactions containing wild-type Ku70/80 (Ku^WT^) or the Ku70^Δ521-528^ mutant (Ku^Δ521-528^). The Ku^Δ521-528^-containing reaction displayed only approximately one-seventh of the ligation efficiency observed with Ku^WT^ (Fig. 1f), further suggesting that the Ku70 histone-bridge region plays an important role in supporting NHEJ-mediated ligation on nucleosomal substrates.

A recent study from Hall et al. reported the structure of an N0-Ku complex^53^, suggesting that Ku can bind nucleosomal substrates even in the absence of an available DNA linker (Supplementary Fig. 3b). Notably, Ku does not occupy in its full footprint in that structure, arguing that Ku does not reach full activity on the N0 substrate (Supplementary Fig. 3a). Given the different linker lengths used in the two studies, it is striking that both nucleosomes are unwrapped to the same position, SHL (−5.5), suggesting that Ku alone cannot outcompete histone-DNA interactions beyond SHL (−5.5) (Supplementary Fig. 3c). Moreover, that study used GraFix for complex reconstitution, in which crosslinking and gradient purification can stabilize transient or weakly associated complexes, explaining why they were able to assemble the complex for cryo-EM while we did not observe a stable N0-Ku complex under NS-EM.

### DNA-PKcs melts the DNA end when recruited to nucleosome-bound Ku

**Fig. 2:**
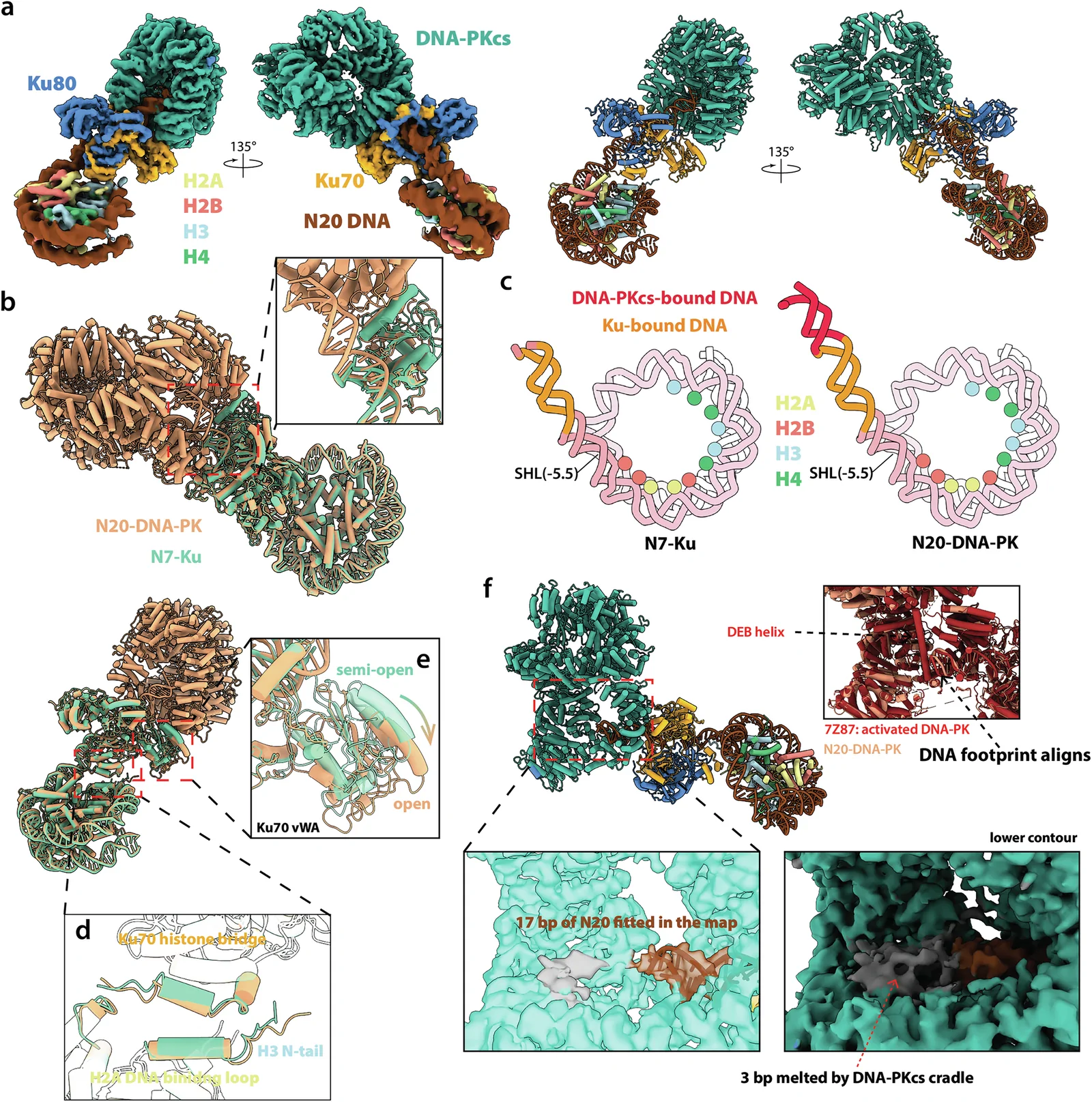
Histone-Ku70 interactions drive DNA end-melting within DNA-PKcs on the N20 substrate. **a** Composite map (left) and structural model (right) of the N20-DNA-PK complex. **b** Superimposed models of N7-Ku (green) and N20-DNA-PK (peach) at a alternate orientation, with a close-up view of the Ku-bound DNA. The front portion of structure is omitted for clarity. **c** Cartoon representations of Ku-bound N7 and DNA-PK-bound N20. DNA engaged by Ku and DNA-PKcs are shown in yellow and red, respectively. Histones are represented by colored circles to indicate DNA contact interfaces. **d** Close-up view of overlapping N7-Ku and N20-DNA-PK models highlighting the Ku70-histone interface. **e** Close-up of the Ku70 vWA domain in N7-Ku and N20-DNA-PK, with an arrow indicating the direction of domain opening. **f** Close-up view of the DNA end in the DNA-PKcs cradle. A comparison of the DNA end between a previously reported activated DNA-PK structure (PDB: 7Z87) and the N20-bound DNA-PK is shown at upper right, where DEB helix is indicated. The front portion of the structure is omitted for clarity. The Cryo-EM density map with different contour levels, with the DNA end docked, are shown at bottom.

Ku recruits DNA-PKcs upon binding to a DNA end, a process well characterized on naked DNA substrates. Similar to Ku, DNA-PKcs also requires ~14 bp of free DNA duplex for assembly, a step accompanied by inward translocation of Ku along the DNA and resulting in a combined footprint of 25–30 bp for the entire complex^49^. This raises the question of how DNA-PK assembles on DNA with restricted accessibility, such as nucleosome linkers. To visualize this process, we first examined the N20 substrate by cryo-EM. A total of 9296 cryo-EM movies were collected, from which ~69,000 particles were picked for reconstruction (Supplementary Fig. 4). The final map yielded a N20-DNA-PK structure with a local resolution of 6.19 Å for the nucleosome and 4.05 Å for the DNA-PK (Fig. 2a). The overall architecture of the N20-DNA-PK complex closely resembles that of the N7-Ku complex (Fig. 2b). In the structure, DNA-PKcs binds to 11 bp along the linker while Ku occupies the next 14 bp relative to the nucleosome (Fig. 2c). This combined footprint (25 bp) of the assembled DNA-PK is similar to the previously reported DNA-PK structures^6,54^. The nucleosomal DNA is again unwrapped from SHL (−7) to SHL (−5.5) in the N20-DNA-PK complex, reproducing the previously observed contacts between Ku70 and histones (Fig. 2d).

On naked DNA substrates, Ku adopts a closed conformation at the Ku70 vWA domain^15,52^. It has been previously shown that recruitment of DNA-PKcs causes the Ku70 vWA domain to move outward, and this opening is further reinforced and stabilized by the subsequent binding of PAXX, resulting in a fully open state^15,55^. In the context of nucleosomal substrates, however, the Ku70 vWA domain exhibits a distinct conformational change (Supplementary Fig. 5). Comparison of the previously reported apo Ku-DNA structure with the N7-Ku structure resolved in this study shows that the PAXX binding pocket in the N7-Ku structure adopts a position similar to that in the open conformation of the vWA domain, while the overall conformation of the Ku70 vWA domain remains closed. Therefore, the Ku70 vWA domain in the N7-Ku structure adopts a semi-open position. In the N20-DNA-PK structure, the Ku70 vWA domain opens fully upon DNA-PKcs binding, occupying a similar conformation as in previously reported PAXX-bound structures (Fig. 2e)^15,55^. These observations suggest that Ku favors a position that is more open to the binding of additional NHEJ factors on the nucleosomal substrate.

The fully assembled DNA-PK is able to protect the DNA end from aberrant end-processing or ligation reactions^56,57^. Depending on the type of DNA ends, DNA-PK undergoes different conformational changes. Upon ATP activation on a blunt DNA end, DNA-PK favors a conformation in which the DEB helix (2737-2767) is ordered; in contrast, when a hairpin end is encountered, the DEB helix becomes disordered^57,58^. Previous cryo-EM structures showed that the terminal base pair of a blunt end DNA end can be separated by the DEB helix in an activated DNA-PK^14,59^. In the N20-DNA-PK structure, we observed indications of a partial DNA end melting (Fig. 2f). Using the nucleosome as a fiducial marker, we modeled the DNA linker into the map precisely. However, only 17 bp of the 20 bp linker could be built into the density map with the DNA termini positions right at the location of the DEB helix in an activated DNA-PK (Fig. 2f), while a lower-contour map revealed additional unassigned density extending from the DNA end, likely corresponding to the missing 3 bp. This observation suggests that interactions between the Ku70 histone bridge and the exposed histones may drive the DNA-PK to thread the DNA end by an additional 3 bp beyond its canonical full footprint, thereby inducing the cradle to partially melt the DNA end.

### DNA-PKcs is able to assist Ku to overcome nucleosomal barriers

**Fig. 3:**
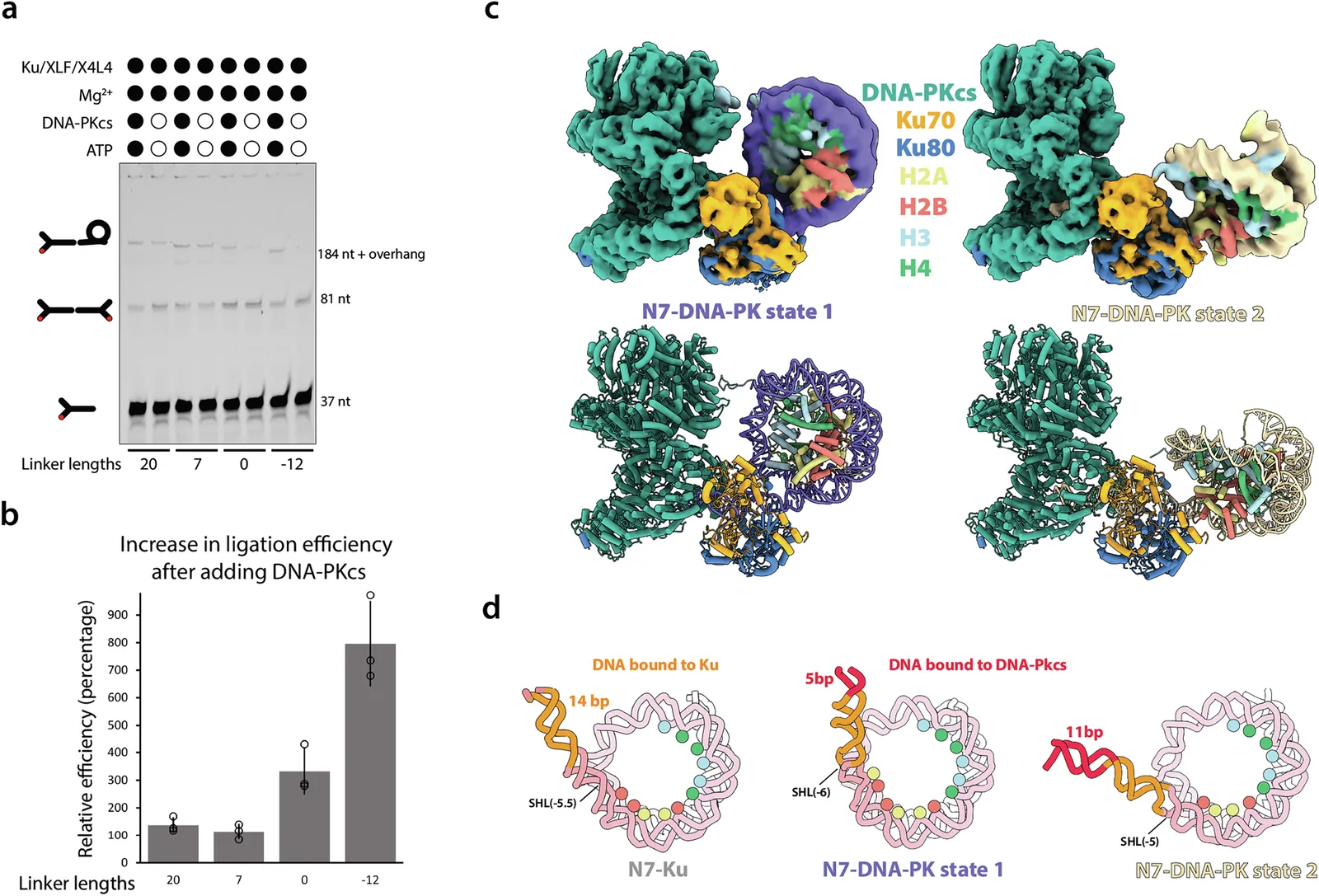
DNA-PKcs promotes increased DNA unwrapping and inward Ku translocation on the N7 substrate. **a** Denaturing TBE-urea gel analysis showing end joining of blunt-end DNA and nucleosomal substrates by components of the LR complex. Source data are provided as a Source Data file. **b** Quantification of the ligation reactions shown in (**a**) from three replicates. The increase in ligation efficiency was calculated as the ratio of ligation efficiency in the presence versus absence of DNA-PKcs for each nucleosome substrate. Individual replicate values are shown for each condition. Error bars are presented as mean values ±SD. *P* value of each condition from left to right: 0.00274, 0.0136, 0.535, 0.105. Source data are provided as a Source Data file. **c** Composite maps (top) and structural models (bottom) of two states resolved for the N7-DNA-PK complex. **d** Cartoon representations of Ku-bound N7 (left), DNA-PK-bound N7 state 1 (middle), and DNA-PK-bound N7 state 2 (right). Histones are represented by colored circles indicating DNA-contact interfaces.

Previous in vitro studies involving ligation activity assays demonstrated that Ku, XLF, and X4L4 are able to mediate end joining in the absence of DNA-PKcs^14,41^. Structural and single-molecule studies further confirmed that DNA-PKcs is not essential for forming NHEJ synaptic complexes under certain conditions^14,26^. These findings suggest that DNA-PKcs plays a dispensable role on naked DNA in vitro. This conclusion is partially true as Ku itself is only able to mediate ligation on nucleosomes with short linkers. To further examine the role of DNA-PKcs, we incorporated it into the ligation assays described above. Addition of DNA-PKcs increased the ligation efficiency of the N-12 substrate by approximately sevenfold and that of the N0 substrate by approximately threefold, whereas the ligation efficiencies of N7 and N20 were only minimally affected (Fig. 3a, b). These results indicate that DNA-PKcs specifically stimulates NHEJ-mediated ligation on nucleosomal substrates lacking accessible free DNA linkers, as Ku itself fails to mediate NHEJ ligation on these substrates.

To elucidate how DNA-PKcs overcomes nucleosomal barriers, we sought to determine the cryo-EM structures of the N0-DNA-PK and the N7-DNA-PK complexes. A cryo-EM dataset was collected on the assembled N0-DNA-PK complex (Supplementary Figs. 6 and 7a). Due to the limited local resolution of the nucleosome density, the nucleosome could only be modeled by rigid-body fitting, whereas the higher-resolution DNA-PK region allowed more detailed model building, particularly at the DNA end within the DNA-PKcs cradle (Supplementary Fig. 7b). Density at the DNA terminus revealed that the DNA-PKcs cradle was not fully occupied. Only 5 bp of DNA were engaged by DNA-PKcs, indicating a partially assembled DNA-PK complex on the N0 substrate. A similar state was reported by Hall et al. in the recent study of DNA-PK assembled on an N3 substrate^53^, where DNA-PKcs likewise exhibited only a 5 bp footprint at the DNA end (Supplementary Fig. 7c, d). To visualize DNA-PK assembly on the N7 substrate, we collected two cryo-EM datasets on the N7-DNA-PK complex at different reaction times (1 min and 15 min) (Fig. 3c, and Supplementary Figs. 8 and 9). Two major states of the complex were resolved from these datasets. In state 1, around 1.5 turns of nucleosomal DNA are unwrapped to SHL (−6), whereas in state 2, an additional turn of nucleosomal DNA is unwrapped to SHL (−5) (Fig. 3d). Similar to N0-DNA-PK from this study and N3-DNA-PK from the other study, state 1 of N7-DNA-PK only exhibits a 5 bp DNA-PKcs footprint. In contrast, state 2 shows a fully assembled DNA-PK with an 11 bp DNA-PKcs footprint. Comparison of the N7-DNA-PK structures with the N7-Ku complex shows additional DNA unwrapping resulted from DNA-PKcs binding (Fig. 3d), suggesting that DNA-PKcs assists Ku to overcome additional nucleosomal barriers by further promoting displacement of nucleosomal DNA.

### DNA-PK captures spontaneously exposed nucleosomal DNA

**Fig. 4:**
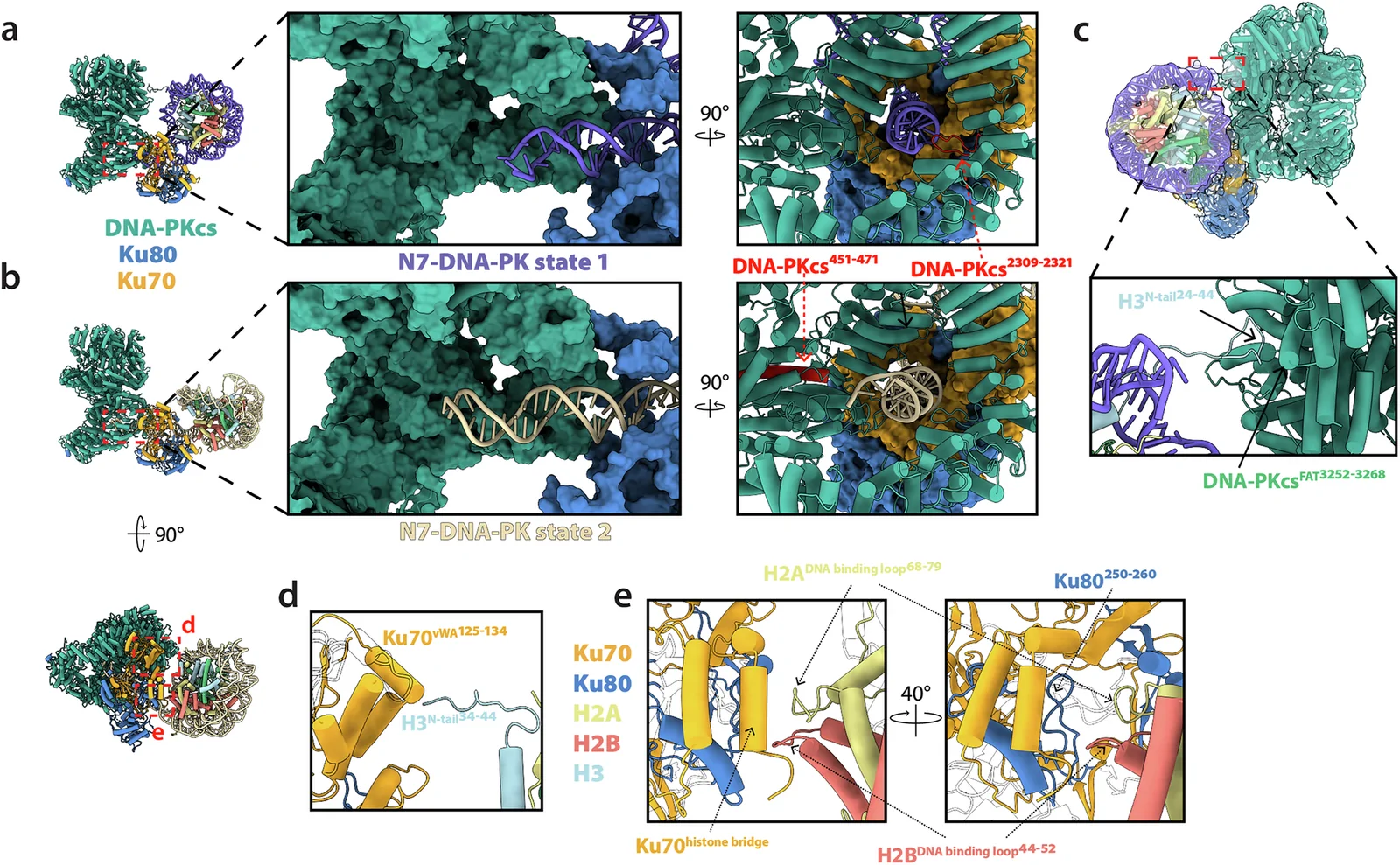
Histone-DNA-PK interactions drive DNA-PK progression on nucleosomes. **a** Close up view of the DNA end of N7-DNA-PK state 1 in the DNA-PKcs cradle. The front portion of the structure is omitted for clarity. **b** Close-up view of the DNA end of N7-DNA-PK state 2 in the DNA-PKcs cradle. **c** Close-up view of the DNA-PKcs FAT domain and the H3 N-tail in N7-DNA-PK state 1. **d**, **e** Close-up views of the interactions between Ku and histones in N7-DNA-PK state 2.

Upon binding of DNA-PKcs, Ku is pushed inward along the DNA and the additional length captured by DNA-PKcs is proposed to facilitate subsequent end-joining^6,14,49^. Using a nucleosomal substrate, we captured an intermediate translocation state of DNA-PK. In the N7-DNA-PK reconstruction, State 1 corresponds to a partially assembled DNA-PK (Fig. 4a). DNA-PKcs in this state engages 5 bp of DNA, filling less than half of the DNA-binding cradle. The DNA end extends only to the MH3 region of the M-HEAT domain and stops near loop 2309-2321. In state 2, by contrast, DNA-PKcs binds 11 bp of DNA and the DNA end reaches the helix 451-471, fully occupying the cradle (Fig. 4b). Comparison to N7-Ku alone reveals that, despite DNA-PKcs binding and more occupation of nucleosomal DNA, less DNA is peeled from the histone surface in state 1 (Fig. 3c, and Supplementary Fig. 10, top). Side-view comparisons show that in N7-Ku and N7-DNA-PK state 2, DNA-PK unwraps more nucleosomal DNA than it requires for binding because the Ku70 vWA domain sterically hinders the unwrapped histones (Supplementary Fig. 10, bottom). The counterclockwise rotational movement of DNA-PK relative to the nucleosome, coupled to its lateral movement, disengages the Ku70 vWA domain from the histones and allows ~5 bp of nucleosomal DNA to rewrap. Together, these observations indicate an ongoing competition between DNA-PK and histone-DNA contacts, with histones rewrapping wherever geometry permits.

In all structures determined here, nucleosomal DNA unwrapping is accompanied by exposure of histone surfaces. In the partially assembled N7-DNA-PK state 1, nucleosomal DNA is unwrapped to SHL (−5.5), exposing only the H3-DNA interface on the entry side (Fig. 3c, mid). Interestingly, no contacts were observed between the exposed H3 and DNA-PK on the proximal side, whereas on the distal side, the H3 N-terminal tail extends directly to the FAT domain of DNA-PKcs (Fig. 4c). The density of the FAT domain of DNA-PKcs merges with the H3 N-terminal tail under a lower contour threshold, suggesting a potential interaction. A similar contact site has been reported for Artemis, which binds the same region of the FAT domain^60^. Additional unassigned density appears near the C-terminus of Ku70. Studies have suggested that the SAP domain participates in Ku binding to DNA by engaging the adjacent DNA and potentially limiting Ku’s lateral movement^53,61–64^ (Supplementary Fig. 11). The position of this extra density appears at the first major groove of the exiting DNA from Ku70/80, consistent with the Ku70 SAP resolved in the previous structure^53^. Together, these observations suggest potential interactions that might trap DNA-PK in its intermediate state on the N7 substrate.

Compared with the N7-Ku structure, state 2 of the N7-DNA-PK complex reveals additional interfaces between Ku and histones. In the fully assembled DNA-PK, ~2.5 turns of nucleosomal DNA are unwrapped, fully exposing the H3 and the H2A/B interface around SHL (−5) (Fig. 3c, right). The proximal exposed H3 N-tail is located near the Ku70 vWA domain (helix 125-134) (Fig. 4d, and Supplementary Fig. 12). Moreover, in addition to the previously described K70 histone bridge, Ku80 also contributes to a potential histone-binding interface: residues 250-260 of Ku80, located in the linker between the Ku80 vWA domain and core domain, and Ku70 helix 521-528 in the histone bridge region, engage H2A loop 68-79 and H2B loop 44-52, respectively, which include residues known to directly interact with nucleosomal DNA^51^ (Fig. 4e, and Supplementary Fig. 12). We also conducted ligation assays comparing Ku^WT^ and Ku^Δ521-528^ with the addition of DNA-PKcs (Supplementary Fig. 13a). Strikingly, the addition of DNA-PKcs increases ligation efficiency in the Ku70^Δ521-528^-containing reaction to a level comparable to that observed with Ku^WT^. This further highlights the ability of DNA-PKcs to rescue ligation defect on nucleosomal substrates.

Besides the two major states, 3D classification identified additional minor states with nucleosomes resolved at lower resolutions (Supplementary Figs. 9 and 14). Comparison of the DNA-PK densities revealed that the same portion of DNA is occupied in both N7-DNA-PK minor states and in state 2 (Supplementary Fig. 14a). Alignment of the DNA-PK densities from these three states showed that the nucleosomal DNA is unwrapped to a similar extent, but in the two minor states fewer DNA-PK-histone interfaces are present, indicating conformations in which the nucleosome has not yet reached its most stable position. Because of their relative flexibility, the resolutions of the two minor states were lower than those of N7-DNA-PK state 2, only allowing for observation of gross structural features. A trajectory of N7 rotating relative to DNA-PK can be reconstructed based on the positions of the nucleosome in all N7-DNA-PK states (Supplementary Fig. 14b).

Comparison of the Ku/DNA-PK-nucleosome structures determined in this study with those reported by Hall et al. reveals a notable shared feature: in all structures, nucleosome unwrapping does not extend beyond approximately SHL (−5) (Supplementary Fig. 15). Studies have shown that nucleosomes are susceptible to spontaneous breathing especially at the entry/exit site^65–67^ and SHL (−5) has been proposed as a major pause site for proteins that encounter the histone barrier, as observed for RNA polymerase II^68^ and the replisome^69^. In these systems, ATP hydrolysis is coupled to active translocation through the nucleosome. By contrast, ATP-dependent DNA translocation has not been reported for the DNA-PK complex. Therefore, DNA-PK assembly on nucleosomal DNA ends likely depends on spontaneous DNA end breathing at the nucleosome entry/exit site. To further examine the contribution of spontaneous DNA breathing, we replaced the Widom 601 nucleosome-positioning sequence with the 5S rRNA nucleosome-positioning sequence, a native sequence that is reported to have a stronger DNA breathing effect and more closely reflects physiological histone-DNA affinity. Using an in vitro ligation assay, we observed a similar pattern in ligation efficiency between Widom 601 and 5S rRNA nucleosomes for either N0 or N7 substrates (Supplementary Fig. 13b). In conclusion, the structural and biochemical results we present here suggest that the interaction between nucleosome and Ku/DNA-PK is very dynamic. Nucleosomal DNA is captured by Ku/DNA-PK through spontaneous end breathing and allowed to rewrap around the histone octamer whenever histones are exposed without contacting DNA-PK.

To assess whether DNA-PK can form long-range synapsis on a chromatinized DNA end, we reconstituted an LR complex on the N7 substrate and analyzed its architecture by negative-stain EM (Supplementary Fig. 16). Rigid-body docking of the LR model (PDB: 7lt3) together with the N7-DNA-PK structure into the negative-stain EM density map revealed that the synaptic configuration is maintained on the nucleosome-containing substrate. However, the nucleosome in the N7-DNA-PK structure sterically clashes with XRCC4 in the LR model (Supplementary Fig. 16). Notably, the density map indicates a slight repositioning of the nucleosome compared to the nucleosome in the N7-DNA-PK structure, suggesting that DNA-PK accommodates the histone core rather than remodeling it during synapsis. These structural findings with the ligation assays demonstrate that DNA-PK can bridge broken DNA ends even in a nucleosomal context, highlighting its capacity to initiate NHEJ without fully displacing the nucleosome.

## Discussion

**Fig. 5:**
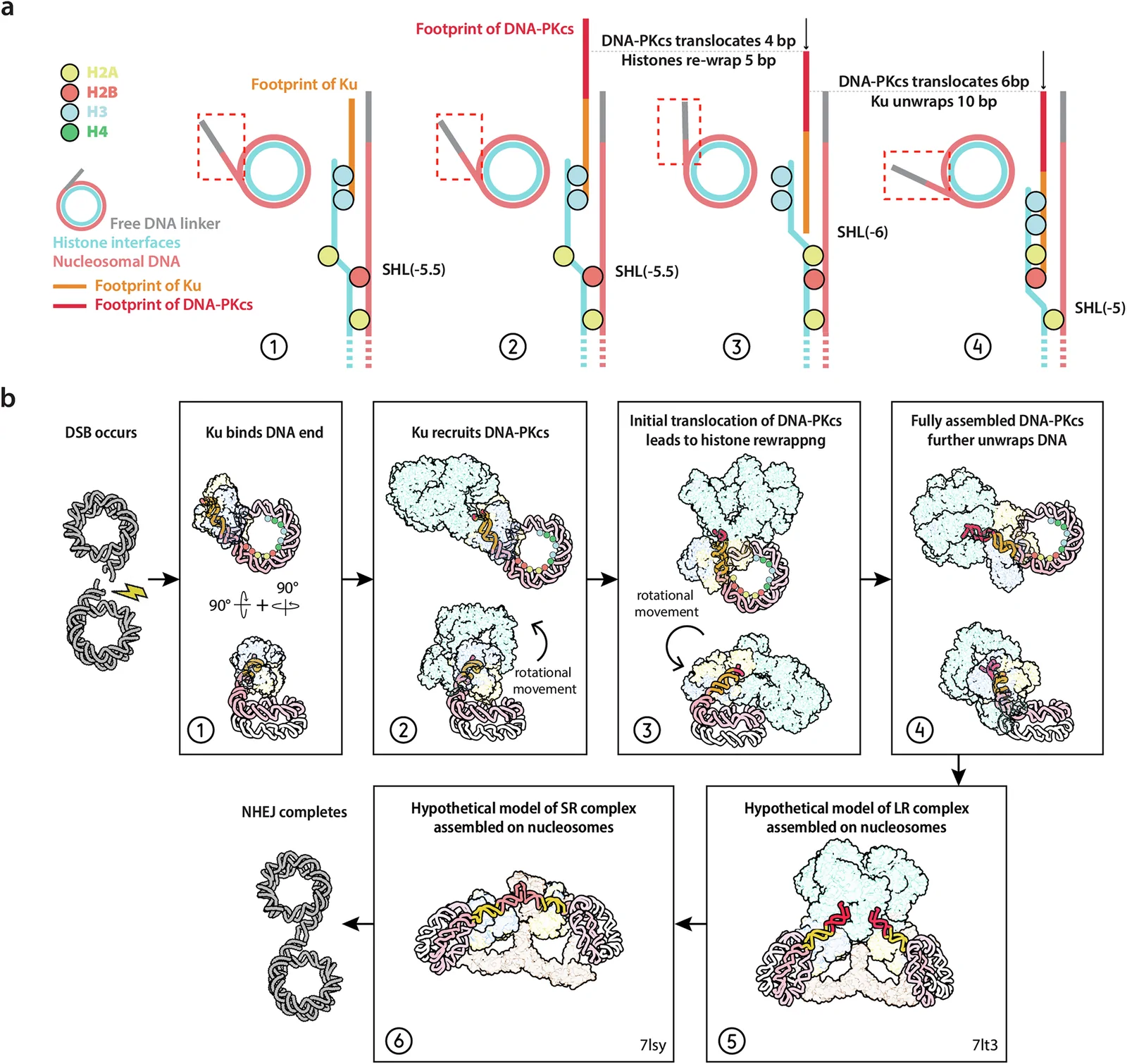
Model of NHEJ initiation on a nucleosome end. **a** Schematic representation of the DNA end illustrating four steps of DNA-PK assembly on N7. DNA-contact interfaces of histones are represented by colored circles. **b** Cartoon representations of the six steps generated from two orientations of each complex. Steps 5 and 6 are hypothetical models generated from fitting the N7-DNA-PK state 2 structure into previously solved LR and SR complex (PDB: 7lt3 and 7lsy). Histones are omitted for clarity.

By integrating the structural snapshots, we propose a stepwise model for DNA-PK assembly on the N7 substrate (Supplementary Video 1). Step 1: Upon the DSB occurring, Ku loads onto the DNA linker, unwrapping ~20 bp of nucleosomal DNA. This exposes H3 and H2A DNA-binding interfaces, which are engaged by the Ku70 histone-bridge region upon Ku binding (Fig. 5, step 1). Step 2: DNA-PKcs is then recruited by Ku. Because Ku occupies the same position relative to the nucleosome in the N7-Ku and the N20-DNA-PK structures, we modified the N20-DNA-PK model to represent a stage in which DNA-PKcs first contacts Ku. DNA-PKcs binding opens the Ku70 vWA domain, preparing Ku for additional NHEJ factors (Fig. 5, step 2). Step 3: To engage its full footprint, DNA-PKcs will translocate along the nucleosomal DNA, pushing Ku inward. DNA-PKcs first translocates Ku helically along the DNA by 4 bp. This initial helical advance relieves the steric hindrance of the Ku70 vWA, allowing H2A that was previously exposed to re-wrap the nucleosomal DNA. Therefore, although DNA-PKcs occupies a longer DNA linker in this state, less nucleosomal DNA is unwrapped than in the first step (Fig. 5, step 3). This partially assembled DNA-PK may be stabilized by Ku70 SAP engagements with adjacent DNA and by a contact between the distal H3 tail and the DNA-PKcs FAT domain. Step 4: DNA-PKcs advances another 6 bp, pushing Ku forward and unwrapping an additional ~10 bp while accommodating the full DNA-PK footprint (Fig. 5, step 4). The fully assembled DNA-PK releases the nucleosomal DNA from the first H2A/H2B interface, which is captured by the Ku70 histone bridge region and the Ku80 linker between its vWA and core domains. Steps 5 and 6: hypothetical models of DNA-PK achieving LR and SR synapsis and permitting NHEJ to carry out repair (Fig. 5, steps 5 and 6).

Although this model is based on the specific trajectory of DNA-PK assembly on the N7 substrate, the underlying mechanism may be generalizable to other nucleosomal substrates. Similar to other chromatin-associated processes, such as RNA polymerase II transcription through the nucleosome, DNA-PK progression along nucleosomal DNA may be constrained by the positions and strengths of histone-DNA contacts. Structural studies reveal that during transcription through a nucleosome, Pol II pauses at specific SHLs, including sites near SHL (−6), (−5), (−2), and (−1). Similarly, the replisome has been reported to pause near SHL (−5) and (−4). In our DNA-PK model, nucleosomal DNA is unwrapped to a similar region around SHL (−5) across multiple substrates, including N20, N7, and N0, as well as the N3 substrate reported by Hall et al. This convergence suggests that SHL (−5) may represent a major barrier to DNA-PK-mediated nucleosome unwrapping. Consistent with this interpretation, DNA near the entry/exit region, particularly from SHL (−7) to (−6), is more prone to spontaneous breathing, whereas histone-DNA interactions become stronger beginning around SHL (−5), where H2A/H2B contribute substantially to DNA binding. This agrees with our structural observations: Ku and DNA-PK appear capable of capturing and stabilizing spontaneously unwrapped entry/exit DNA, but they do not outcompete histone-DNA interactions beyond SHL (−5). Therefore, SHL (−5) likely represents a major stopping point for DNA-PK assembly on various nucleosomal DNA ends.

In summary, our study reveals how DNA-PK assembles in a chromatin context. Through in vitro ligation activity assays, we demonstrate that DNA-PKcs plays an increasingly important role in NHEJ on nucleosomal substrates as DNA linkers shorten. Consistent with this finding, a recent study by Liu et al. also showed that DNA-PKcs enhances NHEJ activity across DNA ends with different end modifications^70^. The positive role of DNA-PKcs in end-bridging and gap-filling under DNA ends that are not directly ligatable was also highlighted by an in vitro ligation assay in that study. Together, these newly uncovered roles of DNA-PKcs in DNA ends with limited accessibility from either chromatinized or modified DNA ends may help reconcile the discrepancies between previous in vivo*/*ex vivo and in vitro observations. Using cryo-EM, we identify the ability of DNA-PK to unwrap nucleosomes during assembly and uncover potential interfaces between histones and DNA-PK. Future studies investigating subsequent NHEJ steps, particularly the formation of synaptic complexes on nucleosomal substrates, will provide a more complete picture of NHEJ within chromatin. Moreover, exploring the coordination between chromatin remodelers and NHEJ factors may further illustrate how NHEJ machinery dynamically engages chromatin during DSB repair.

## Methods

### Purification of protein factors

Nuclear cell pellets from 50, 100 or 300 L batches of logarithmically-growing HeLa cells were purchased from the National Cell Culture Center/Biovest International, Wisconsin. Ku and DNA-PKcs were purified from the nuclear pellet of HeLa cells as previously described^71^^,^^72^. The final concentration of DNA-PKcs was 0.3 mg ml^−1^ and Ku70/80 1.53 mg ml^−1^. The LigIV-XRCC4 complex was purified from baculovirus-infected insect cells, and the final concentration was 7.9 mg ml^−1^^72^. XLF was purified as previously described, and the final concentration was 2.2 mg ml^−1^^73^.

### Ku mutagenesis

Primers designed to delete Ku70 residues 521-528 were obtained from IDT. The Ku70/80 plasmid was mutated by PCR, and recombinant bacmid DNA was generated and transfected into Sf9 cells. Cells were lysed by sonication and centrifuged to obtain the nuclear extract. The nuclear extract was purified using anti-His resin and washed with high-salt buffer containing 1 M NaCl. Bound protein was eluted using an imidazole gradient from 5 mM to 250 mM. Elution fractions containing mutant Ku70/80 were pooled and concentrated using Amicon Ultra 30 kDa MWCO concentrators. To further purify mutant Ku70/80, the partially purified sample was centrifuged through a 10–30% glycerol gradient at 20,000 rpm for 20 h. The gradient was fractionated, and fractions were analyzed by 4-12% SDS-PAGE. Fractions containing Ku70/80 were pooled, concentrated, flash-frozen, and stored at −80 °C until use.

### Nucleosome reconstitution

Mono-nucleosomes were reconstituted with Xenopus histones and the 601 DNA^45^ or 5 s RNA DNA using the Mini Prep Cell (Bio-rad) as described previously^74^. The Xenopus histones were obtained from the Histone Source - the Protein Expression and Purification (PEP) Facility at Colorado State University. Nucleosomal DNA was PCR amplified from a plasmid containing the 601 DNA sequence of 147 bp. Primers containing different 5′ modifications with various lengths of linkers were designed and used for DNA amplification and nucleosome assembly. After reconstitution, the concentration of the nucleosome is measured by Nanodrop and is in the range of 0.4 μM to 1 μM.

### In vitro ligation assay

All oligonucleotide sequences used in this study are available in the Supplementary Data 1 file. Cy5-labeled Y-shaped DNA used in ligation assay was designed and prepared as previously described^14^. For ligation assay without DNA-PKcs, 0.1 μL of 2 μM Y30 DNA and an equimolar amount of nucleosome were each incubated separately with Ku at a 1:2 molar ratio for 5 min in assembly buffer (50 mM Tris, 50 mM KCl, 5 mM EDTA, 5% Glycerol, 1 mM DTT, and 0.001% NP-40). The Y30 and nucleosome halves were then combined with X4L4 and XLF at a molar ratio of 1:1:1 to Ku and incubated for 15 min. Ligation was induced by adding 15 mM MgCl_2_ and continuing the reaction for 1 h. For ligation assays with DNA-PKcs, DNA-PKcs was incubated with the reaction mixture for 5 min before the addition of X4L4 and XLF. The reaction was then induced with 10 mM ATP and 15 mM MgCl2 and incubated for 1 h. Reaction products were quenched by adding 1 mM proteinase K for 15 min at 37 °C, followed by a 5 min boiling step in 50% formamide and 50 mM EDTA. Reaction products were then analyzed on a 10% UREA-TBE gel and imaged using an Azure 600 Imaging System.

### Assembly of the Ku-bound and DNA-PK-bound nucleosomes

For N7-Ku and N0-Ku complexes, 2 μL of each nucleosomal substrate were first incubated with T1 Streptavidin Magnetic Beads for 15 min in 10 μL assembly buffer (50 mM HEPES, 50 mM KCl, 5 mM EDTA, 5% glycerol, 1 mM DTT, and 0.001% NP-40). Ku was then added at a 1:1 molar ratio to the substrate and incubated for 5 min. Following three washes with assembly buffer, the bound complex was eluted with 3 μL assembly buffer containing 2% EcoRI for 30 min. Elution was then cross-linked with 0.0625% glutaraldehyde for 5 min on ice before EM analysis.

For N7-DNA-PK (state 1), 2 μL of each nucleosomal substrate was similarly incubated with T1 Streptavidin Magnetic Beads for 15 min in 10 μL assembly buffer, followed by addition of Ku at a 1:1 molar ratio and incubation for 5 min. After three washes, complexes were eluted with 3 μL assembly buffer containing 2% EcoRI. The eluted Ku-nucleosome complexes were then incubated with DNA-PKcs for 1 min before cross-linking, using a concentration determined by negative-stain EM to achieve an approximate 1:1 molar ratio between Ku-bound nucleosome and free DNA-PKcs.

For N0-DNA-PK, N20-DNA-PK, N7-DNA-PK (state 2) complexes, 2 μL of the desthio-biotinylated nucleosomal substrate was incubated with T1 Streptavidin Magnetic Beads for 15 min in 10 μL assembly buffer, followed by sequential addition of Ku and DNA-PKcs at 1:1 molar ratios, for 5 min and 15 min, respectively. After three washes, the assembled complex was eluted using 3 μL assembly buffer supplemented with 1 mM biotin. Elution was then cross-linked with 0.0625% glutaraldehyde for 5 min on ice before EM analysis.

### **Assembly of N7-LR complex**

30 picolemoles of N7 nucleosome were incubated with Ku, DNA-PKcs, X4L4, XLF, and PAXX in a 1.5:1:1:1:1:1 molar ratio for 20 min in the assembly buffer (50 mM NaCl, 10 mM MgCl_2_, 5 mM EDTA, 5% Glycerol, 1 mM DTT, and 0.001% NP-40). Then, GraFix^75^ was used to purify the complex. The sample was added to the top of a 2 mL 10–30% glycerol gradient, prepared using a BioComp Gradient Master 108. After centrifugation at 24,000 rpm for 16 h at 4 °C in a TLS-55 rotor using an Optima MAX-TL Ultracentrifuge (Beckman Coulter), the sample was split into 100 µL fractions and the crosslinking reaction was quenched with 50 mM Tris-HCl. Non-denaturing PAGE revealed a peak corresponding to the N7-LR complex, which was subsequently pooled, cross-linked with 0.0625% glutaraldehyde for 15 min on ice, quenched with 50 mM Tris, and concentrated to a final volume of 20 µL using Amicon Ultra 0.5 mL centrifugal filters with a 30,000 MW cutoff (Milipore).

### Electron microscopy

NS-EM was performed as previously described^14^. Samples were imaged on a FEI Talos 200C FEG transmission electron microscope operated at 200 kV, equipped with Ceta and Falcon II direct detection CMOS cameras. For N0-Ku, N7-Ku, and N7-LR samples, 78, 81, and 571 micrographs were collected, respectively, at a nominal magnification of 45,000×, corresponding to a pixel size of 3.7 Å, using EPU software with a defocus of approximately −2.0 µm.

For cryo-EM, graphene oxide (GO) grids were prepared in-house^76^ by applying GO to glow-discharged Quantifoil R2/1, 300-mesh Cu grids. Grids were glow-discharged using a Tergeo EM plasma cleaner (Gatan) at 5 mA for 10 s before GO application. Grids were vitrified in a Vitrobot Mark IV (Thermo Fisher Scientific) operating at 4 °C and 100% humidity. For each complex, 3 µL sample were applied and incubated on the grid for 7 min, followed by blotting with a force of 10 for 3 s and plunge-freezing into liquid ethane. Frozen grids were stored in liquid nitrogen until imaging.

The N20-DNA-PK dataset was collected at the Pacific Northwest Center for Cryo-EM (PNCC). A total of 9296 movies were recorded on a Titan Krios G3 transmission electron microscope (Thermo Fisher Scientific) operated at 300 kV, equipped with a Gatan K3 direct electron detector and a BioContinuum energy filter (10 eV slit width). Movies were acquired at a nominal magnification of 81,000× (corresponding to 0.4135 Å/pixel in super-resolution mode) using SerialEM^77^ with a total exposure of 40 e^-^/Å² over 50 frames in 3.162 s, and a defocus range of −2.0 µm to −4.0 µm.

Cryo-EM datasets for N7-Ku, N7-DNA-PK (state 1), N7-DNA-PK (state 2), and N0-DNA-PK were collected at the Johns Hopkins Beckman Center for CryoEM. A total of 7095, 8993, 7209, and 20,977 movies were recorded, respectively, on a Titan Krios G3 microscope (Thermo Fisher Scientific) operating at 300 kV, equipped with a Falcon 4i direct electron detector and a Selectris energy filter. Data were collected at a nominal magnification of 120,000× (0.93 Å/pixel) using EPU, with a total exposure of 60 e^-^/Å² and a defocus range of −2.0 µm to −4.0 µm.

### Image processing and 3D reconstruction

All NS-EM and cryo-EM data were processed using CryoSPARC v4.6^78^. For the N0-Ku and N7-Ku negative-stain datasets, micrographs were imported with a constant CTF value and subjected directly to blob picking. A total of 30,745 and 50,833 particles, respectively, were picked, extracted, and 2D-classified using box sizes of 164 pix and 188 pix. For the N7-LR dataset, 42,679 particles were selected after iterative 2D classification and used for an initial homo-refinement to obtain a 3D reconstruction. Subsequent 3D classification yielded a subset of 14,232 particles that were used for the final homo-refinement.

For the N20-DNA-PK cryo-EM dataset, movies were first motion-corrected and CTF-estimated using CryoSPARC’s patch-based algorithms. Blob picking yielded 1,548,937 particles, which were extracted with a box size of 448 pixels (binned by 4) and subjected to multiple rounds of 2D classification to remove junk particles, resulting in 688,836 particles for downstream processing. A previously resolved DNA-PK map (EMD-11217^79^) was used as the initial reference for homogeneous refinement, followed by a global 3D classification. A subset of 116,244 particles exhibiting clear nucleosome density was re-extracted (binned by 2) and further refined. ChimeraX^80^ was used for volume segmentation and subsequent mask generation. Local 3D classification using a mask around the nucleosome density yielded 68,925 particles with well-defined nucleosome features, which were unbinned and subjected to local refinement with a focused mask on DNA-PK, producing a 3.93 Å reconstruction. Subsequent local 3D classification on the nucleosome density yielded 25,710 particles used to obtain a 6.19 Å nucleosome reconstruction. The two locally refined maps were finally merged in ChimeraX using the *volume maximum* function.

Similar initial procedures were used to process cryo-EM datasets for N7-Ku, N7-DNA-PK (state 1), N7-DNA-PK (state 2), and N0-DNA-PK. Movies were first motion-corrected and CTF-estimated using CryoSPARC’s patch-based algorithms. Blob-picked particles (256 pix box size for N7-Ku dataset and 480 pix box size for the rest of the nucleosome-bound DNA-PK datasets) were subjected to non-uniform refinement against previously determined reference maps (EMD-4704^81^ for N7-Ku and EMD-11217^79^ for nucleosome-bound DNA-PK). The resulting reconstructions were used to perform 3D classification with a 15 Å resolution limit to remove junk particles. Particles from well-defined classes were subsequently used to train a Topaz model^82^, which was then applied to pick an expanded particle set for each dataset. These particles were used for downstream processing, and a composite map was generated using locally refined maps in ChimeraX.

For the N7-Ku dataset, a total of 2,949,075 particles were initially picked and extracted with a box size of 256 pixels (binned by 4). After the initial 3D classification, 180,318 particles were retained and used to train a Topaz model, which yielded 689,691 particles upon re-picking for ab initio reconstruction. A subsequent global 3D classification produced 229,124 particles, which were further subjected to masked 3D classification focused on the Ku density. The remaining 81,691 particles were re-extracted without binning and used for masked local refinement on the nucleosome, resulting in a 3.89 Å reconstruction of the nucleosome. A final 3D classification focused on the Ku density yielded 25,211 particles, which were used to obtain the final Ku reconstruction at 5.97 Å.

For the N7-DNA-PK (state 1) dataset, 1,654,220 particles were initially picked by blob picking. After 3D classification, 247,592 particles were retained and used to train a Topaz model, which yielded 605,248 particles upon re-picking. A global 3D classification produced a subset of 221,770 particles, which were used to reconstruct the DNA-PK density at 3.88 Å resolution. Subsequent masked 3D classification and local refinement focusing on the nucleosome density resulted in a reconstruction at 7.92 Å using 68,908 particles.

For the N7-DNA-PK (state 2) dataset, blob picking identified 2,882,079 particles, of which 211,600 particles remained after initial 3D classification. These particles were used to train a Topaz model, which subsequently yielded 631,461 particles. A global 3D classification retained 186,786 particles, which were further subjected to a masked 3D classification focused on the nucleosome density, resulting in three well-defined classes (134,390 particles in total) corresponding to nucleosomes in slightly different orientations (43,041, 44,539, and 46,810 particles per class). As the DNA-PK density was consistent among these three classes, the combined 134,390 particles were used to obtain a 3.83 Å reconstruction of DNA-PK. Two of the three classes were not able to be further improved and subsequent local refinement of the other class resulted in a 6.65 Å nucleosome reconstruction.

For the N0-DNA-PK dataset, a total of 3,638,917 particles were initially blob-picked and extracted using a box size of 480 pixels (binned by 4). 3D classification with a low-resolution limit identified 247,592 particles exhibiting clear DNA-PK density, which were used to train a Topaz model, yielding 417,828 particles upon re-picking. A subsequent global 3D classification retained 108,426 particles with well-defined DNA-PK density, which were re-extracted with 2× binning. Masked local refinement of these particles produced a map with 3.8 Å resolution for the DNA-PK region. Further 3D classification on these particles yielded a class containing 23,661 particles with well-resolved nucleosome density, which, after local refinement, resulted in a 9.98 Å nucleosome reconstruction.

### Model building

Previously published cryo-EM structures of the nucleosome (PDB: 1KX5^51^), Ku (PDB: 1JEY^52^), and DNA-PK (PDB: 6ZHA^79^) were used as initial templates for model building. These models were rigid-body docked into the newly reconstructed cryo-EM maps using ChimeraX. The DNA linkers of the nucleosomes were generated using AlphaFold3^83^ and manually connected to the docked model in ChimeraX. The combined model was then manually rebuilt in ISOLDE^84^, mutated in Coot^85^, and refined using Phenix real-space refinement^86^.

### Reporting summary

Further information on research design is available in the Nature Portfolio Reporting Summary linked to this article.

## Supplementary information

Supplementary Information

Description of Additional Supplementary Files

Supplementary Data 1

Supplementary Video 1

Reporting Summary

Transparent Peer Review file

## Source data

Source data

## Data Availability

Cryo-EM density maps and model coordinates have been deposited in the Electron Microscopy Data bank (EMDB) and Protein Data Bank (PDB): EMDB-74422 and pdb_00009zmm (N7-Ku); EMDB-74444 and pdb_00009znc (N20-DNA-PK); EMDB-74512 and pdb_00009zp0 (N0-DNA-PK); EMDB-74524 and pdb_00009zp9 (N7-DNA-PK state 1); EMDB-74481 and pdb_00009zob (N7-DNA-PK state 2). Previously deposited PDBs used in this study include: 1KX5, 1JEY, 6ZHA, 9IGW, 7Z87, 8EZA, 9QCR, 7LT3, 7LTY. All remaining data needed to evaluate the conclusions in the paper are present in the paper and its Supplementary Materials. Source Data are provided with this paper.
