# Supplementary material for: DNA-PK driven nucleosome unwrapping enables NHEJ in chromatin
Source: Nat Commun. 2026 Sep 4;17:10545. doi: 10.1038/s41467-026-77534-9 (PMC13642939; doi:10.1038/s41467-026-77534-9)
Supplement: Supplementary file 1 [file 41467_2026_77534_MOESM1_ESM.pdf]

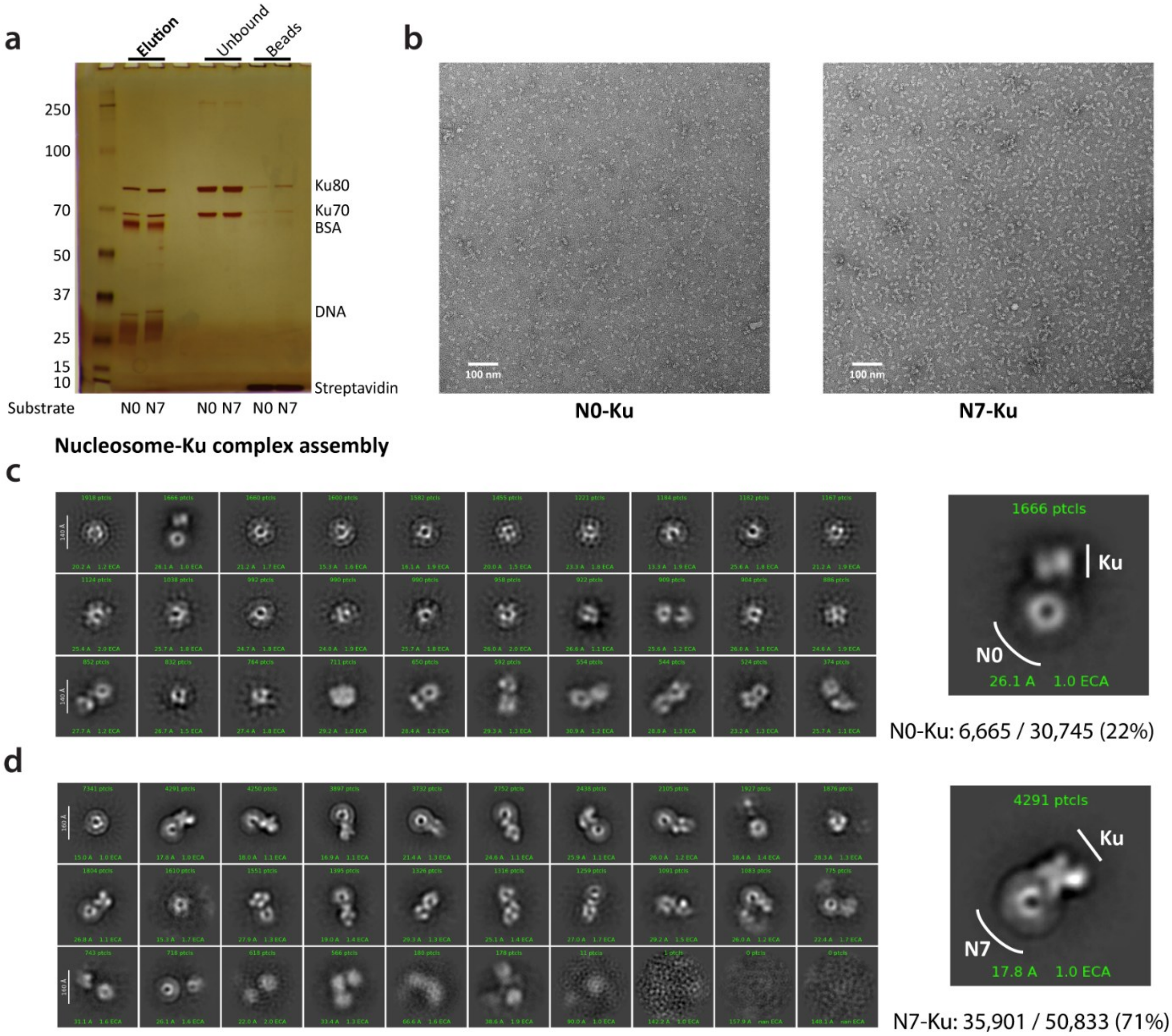

e

```
Human        VEAMNKRLGSLVDEFKELVYPPDY   534
Chimpanzee   VEAMNKRLGSLVDEFKELVYPPDY   534
Mouse        VEAIKKRLGSLADEFKELVYPPGY   532
Chick        TEEMSRRLGNLVEEFKQLVYPPDY   556
             .*  :.:***.*.:***:*****.*
```

Supplementary Figure 1. Comparison of NS-EM data between N0-Ku and N7-Ku.

(a) 4-12% Bis-Tris SDS-PAGE analysis of N0-Ku and N7-Ku complex assembly. Elution, unbound protein, and remaining bead lanes are labeled. Bands corresponding to Ku, BSA, DNA, and streptavidin are indicated. Source data are provided as a Source Data file. (b) Representative micrographs of the N0-Ku sample (left) and N7-Ku sample (right). Dataset information, including the number of micrographs, pixel size, and total particles picked, is shown below each image. (c, d) 2D classification results of the N0-Ku (c) and N7-Ku (d) datasets. The percentage of particles corresponding to nucleosome-bound Ku is indicated on the right. (e) Sequence alignment of human Ku70 residues 511-534 with the corresponding region of Ku from three other species, generated using the EMBL-EBI database. Sequence similarity is indicated below by standard symbols.

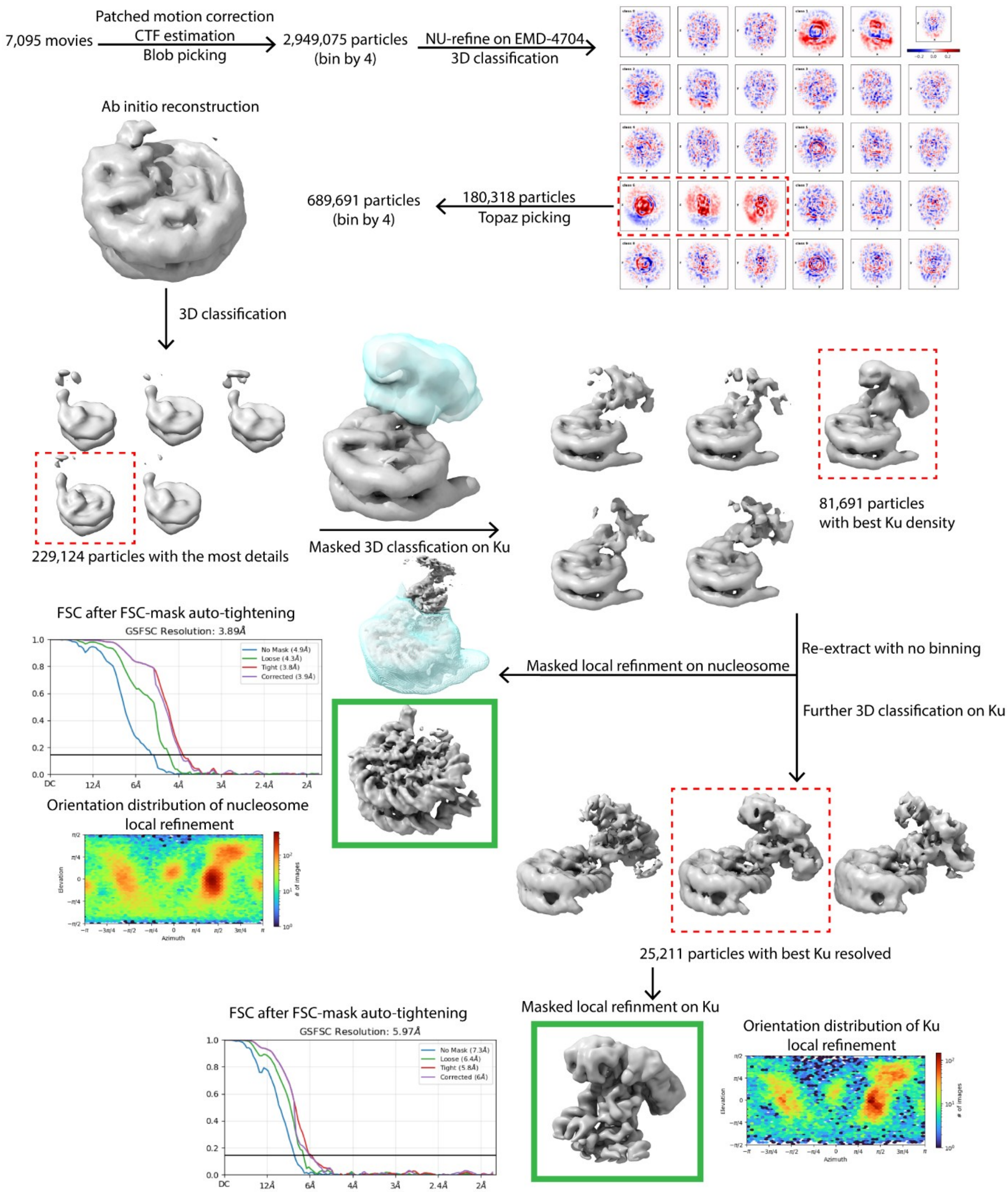

7,095 movies
Patched motion correction
CTF estimation
Blob picking
2,949,075 particles
(bin by 4)
NU-refine on EMD-4704
3D classification
Ab initio reconstruction
689,691 particles
(bin by 4)
180,318 particles
Topaz picking
3D classification
229,124 particles with the most details
Masked 3D classfication on Ku
81,691 particles
with best Ku density
FSC after FSC-mask auto-tightening
GSFSC Resolution: 3.89Å
No Mask (4.9Å)
Loose (4.3Å)
Tight (3.8Å)
Corrected (3.9Å)
Masked local refinment on nucleosome
Re-extract with no binning
Further 3D classification on Ku
Orientation distribution of nucleosome
local refinement
Elevation
Azimuth
# of images
25,211 particles with best Ku resolved
Masked local refinment on Ku
FSC after FSC-mask auto-tightening
GSFSC Resolution: 5.97Å
No Mask (7.3Å)
Loose (6.4Å)
Tight (5.8Å)
Corrected (6Å)
Orientation distribution of Ku
local refinement

Supplementary Figure 2. Data-processing scheme of the N7-Ku complex sample.

Flow chart of the cryo-EM data processing procedure. The gold-standard Fourier shell correlation curves (0.143 cut-off) show the final resolutions of the nucleosome and the Ku.

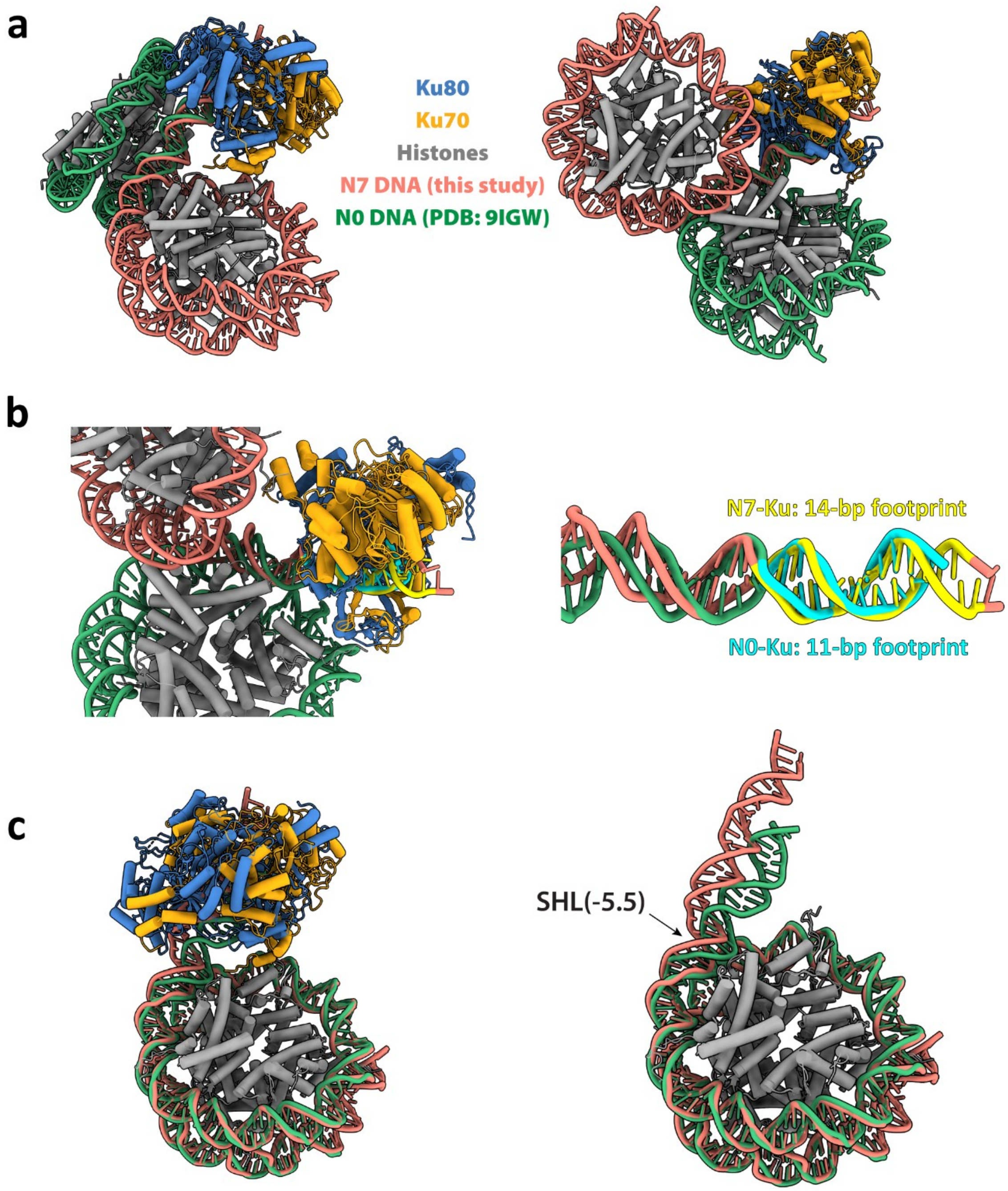

a
Ku80
Ku70
Histones
N7 DNA (this study)
N0 DNA (PDB: 9IGW)
b
N7-Ku: 14-bp footprint
N0-Ku: 11-bp footprint
c
SHL(-5.5)

Supplementary Figure 3. Comparison between N7-Ku structure (this study) and N0-Ku structure (9IGW).

(a) Two models aligned on Ku70/80. Left: front view of N7-Ku (same view as Figure 1c). Right: Front view of N0-Ku at a similar angle to the left. (b) Close-up views of DNA footprint of Ku. The structure of Ku is hidden on the right to show base pair alignment of two DNA footprints. (c) Two models aligned on nucleosome. The structure of Ku is hidden on the right for clarity. Both nucleosomes are unwrapped until SHL (-5.5).

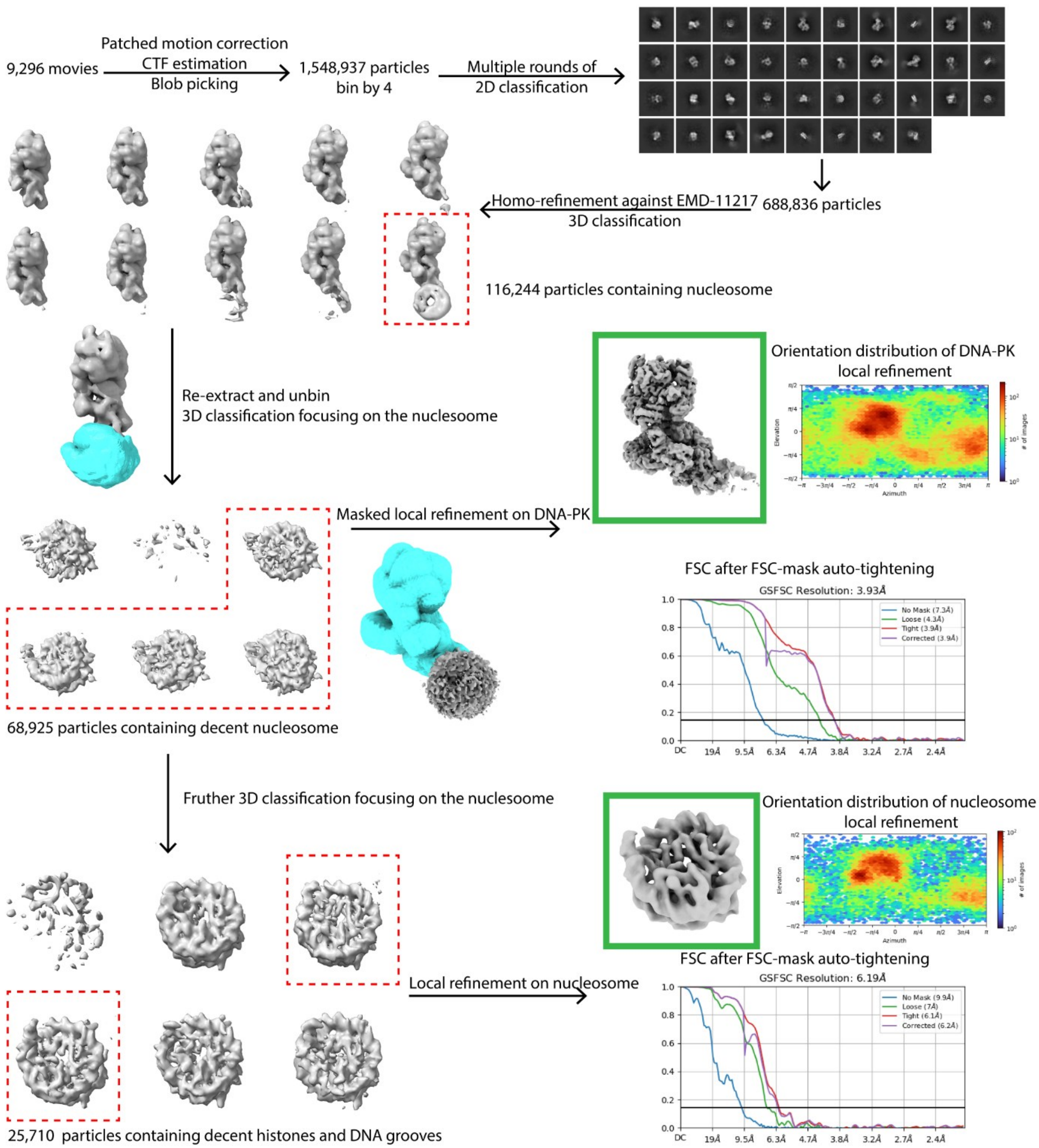

Patched motion correction
CTF estimation
Blob picking
9,296 movies
1,548,937 particles
bin by 4
Multiple rounds of
2D classification
688,836 particles
Homo-refinement against EMD-11217
3D classification
116,244 particles containing nucleosome
Re-extract and unbin
3D classification focusing on the nuclesoome
Orientation distribution of DNA-PK
local refinement
Masked local refinement on DNA-PK
FSC after FSC-mask auto-tightening
GSFSC Resolution: 3.93Å
No Mask (7.3Å)
Loose (4.3Å)
Tight (3.9Å)
Corrected (3.9Å)
68,925 particles containing decent nucleosome
Fruther 3D classification focusing on the nuclesoome
Orientation distribution of nucleosome
local refinement
FSC after FSC-mask auto-tightening
GSFSC Resolution: 6.19Å
No Mask (9.9Å)
Loose (7Å)
Tight (6.1Å)
Corrected (6.2Å)
Local refinement on nucleosome
25,710 particles containing decent histones and DNA grooves

Supplementary Figure 4. Data-processing scheme of the N20-DNA-PK complex sample.

Flow chart of the cryo-EM data processing procedure. The gold-standard Fourier shell correlation curves (0.143 cut-off) show the final resolutions of the nucleosome and the DNA-PK.

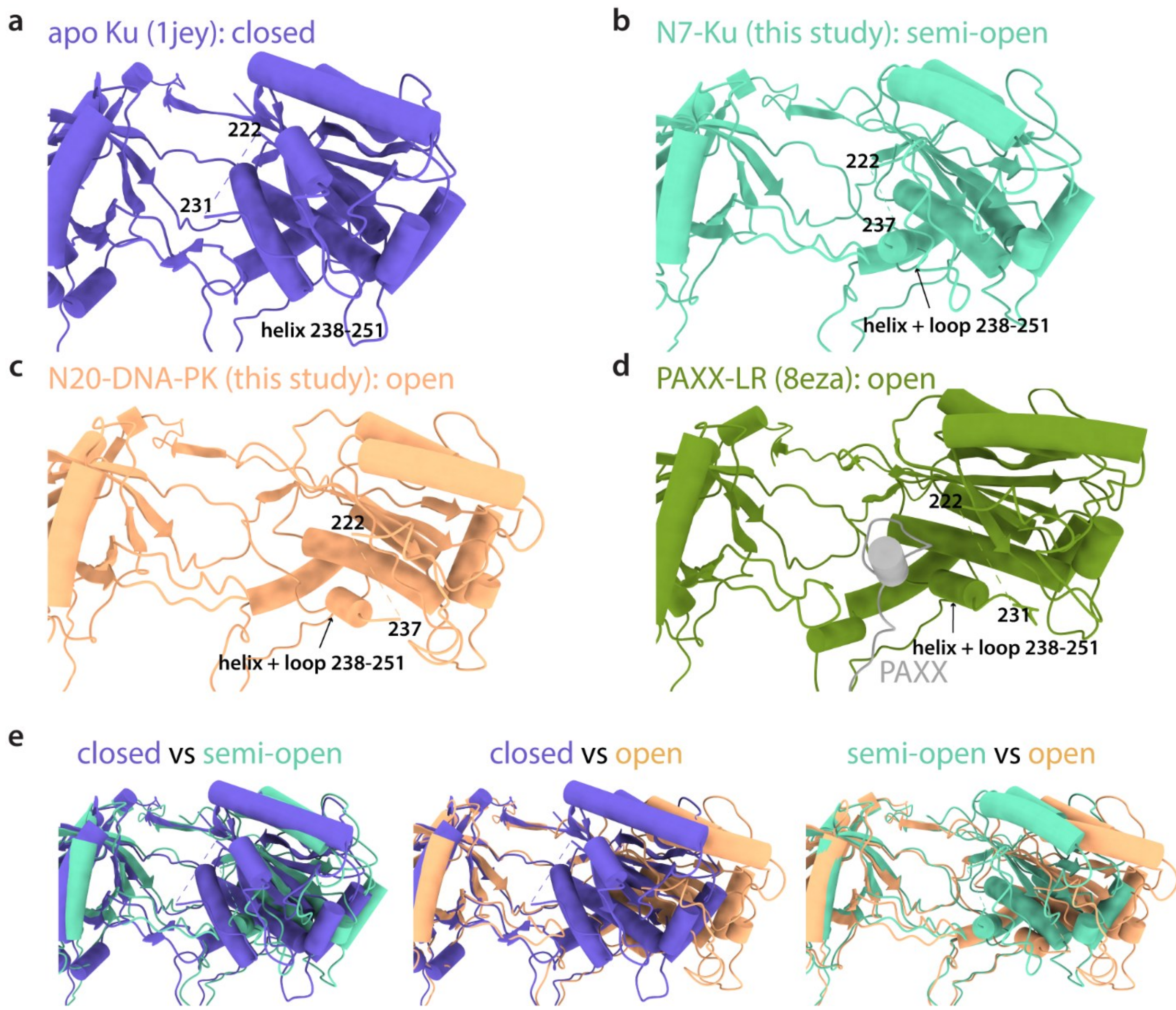
a
apo Ku (1jey): closed
222
231
helix 238-251
b
N7-Ku (this study): semi-open
222
237
helix + loop 238-251
c
N20-DNA-PK (this study): open
222
237
helix + loop 238-251
d
PAXX-LR (8eza): open
222
231
helix + loop 238-251
PAXX
e
closed vs semi-open
closed vs open
semi-open vs open

Supplementary Figure 5. Comparison of Ku70 vWA domains in different states.

(a-d) Structures of Ku70/80 bound to (a) naked DNA duplex, (b) N7-Ku, (c) N20-DNA-PK, and (d) the LR complex with PAXX (shown in grey). All components except Ku70 are omitted for clarity. Missing residues in each structure are labeled. Residues involved in PAXX binding are indicated to highlight the conformational changes of the binding pocket. (e) Comparisons between different states.

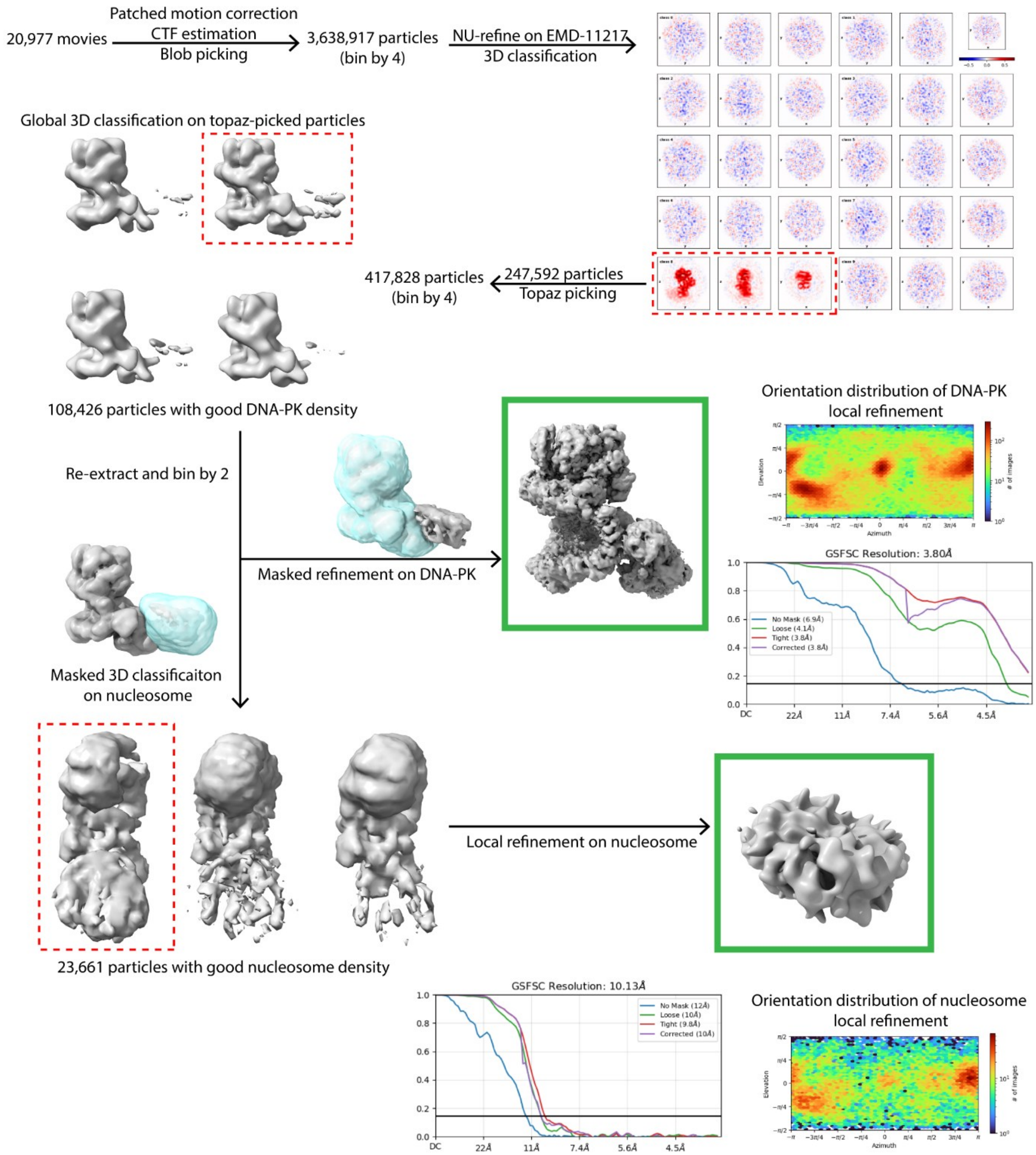

20,977 movies
Patched motion correction
CTF estimation
Blob picking
3,638,917 particles
(bin by 4)
NU-refine on EMD-11217
3D classification
Global 3D classification on topaz-picked particles
417,828 particles
(bin by 4)
247,592 particles
Topaz picking
108,426 particles with good DNA-PK density
Orientation distribution of DNA-PK
local refinement
Re-extract and bin by 2
Masked refinement on DNA-PK
GSFSC Resolution: 3.80Å
No Mask (6.9Å)
Loose (4.1Å)
Tight (3.8Å)
Corrected (3.8Å)
Masked 3D classificaiton
on nucleosome
Local refinement on nucleosome
23,661 particles with good nucleosome density
GSFSC Resolution: 10.13Å
No Mask (12Å)
Loose (10Å)
Tight (9.8Å)
Corrected (10Å)
Orientation distribution of nucleosome
local refinement

Supplementary Figure 6. Data-processing scheme of the N0-DNA-PK complex sample.

Flow chart of the cryo-EM data processing procedure. The gold-standard Fourier shell correlation curves (0.143 cut-off) show the final resolutions of the nucleosome and the DNA-PK.

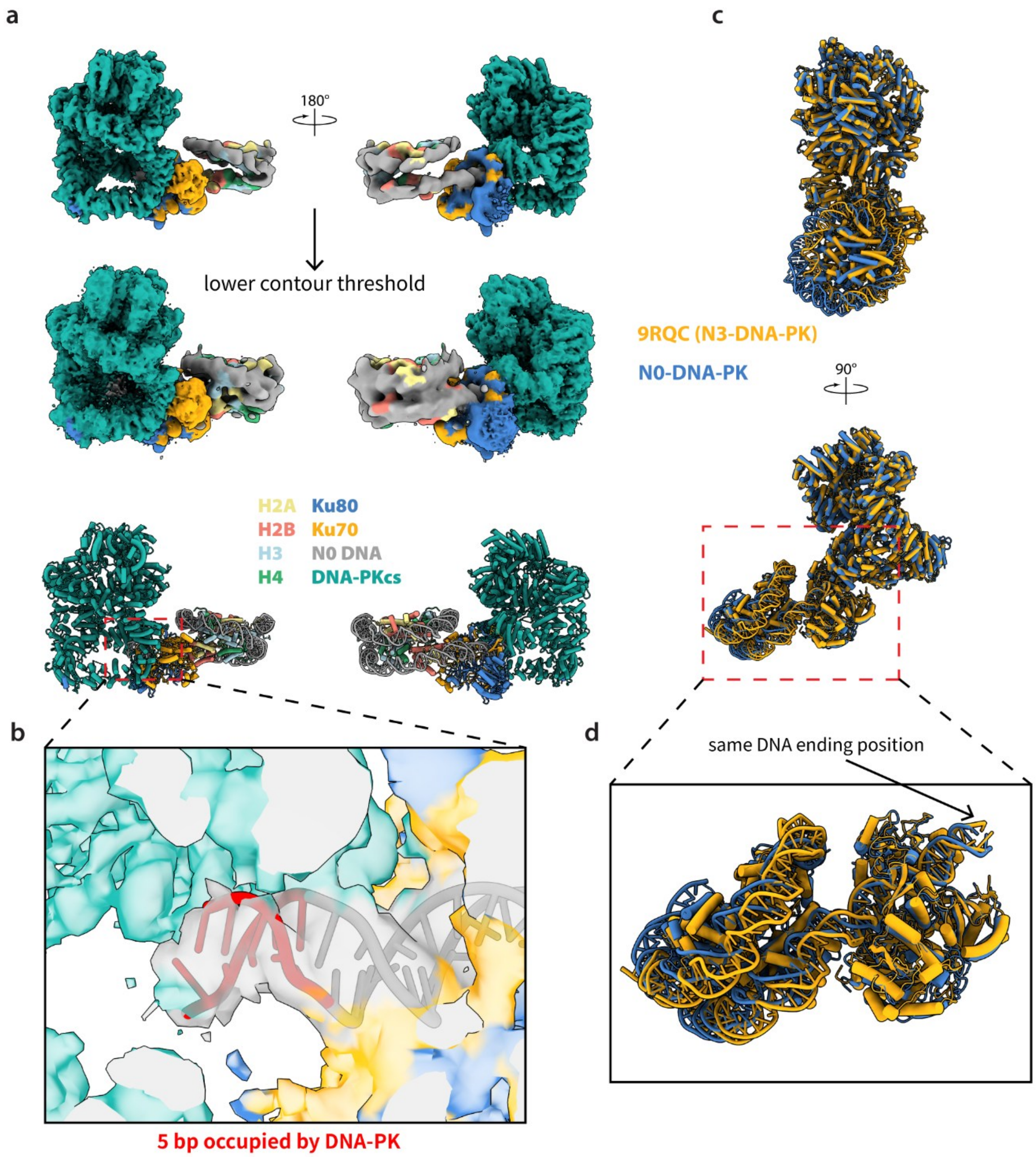
a
180°
lower contour threshold
H2A
H2B
H3
H4
Ku80
Ku70
N0 DNA
DNA-PKcs
b
5 bp occupied by DNA-PK
c
9RQC (N3-DNA-PK)
N0-DNA-PK
90°
d
same DNA ending position

Supplementary Figure 7. Comparison of structures between N0-DNA-PK (this study) and N3-DNA-PK (PDB: 9QRC).

(a) Density map of N0-DNA-PK. Two angles and two different contour threshold levels are shown. (b) N0-DNA-PK model built from the density map and the zoomed-in view of the DNA termini in the DNA-PKcs cradle. DNA is fitted to show the quality of the map at the DNA termini. The 5 bp occupied by DNA-PK is marked by red color. (c) Superimposed models of N0-DNA-PK (blue, this study) and N3-DNA-PK (yellow, 9QRC). (d) Close-up view of overlapping nucleosome from N0-DNA-PK and N3-DNA-PK. The same position where the DNA linkers end in both structures is labeled.

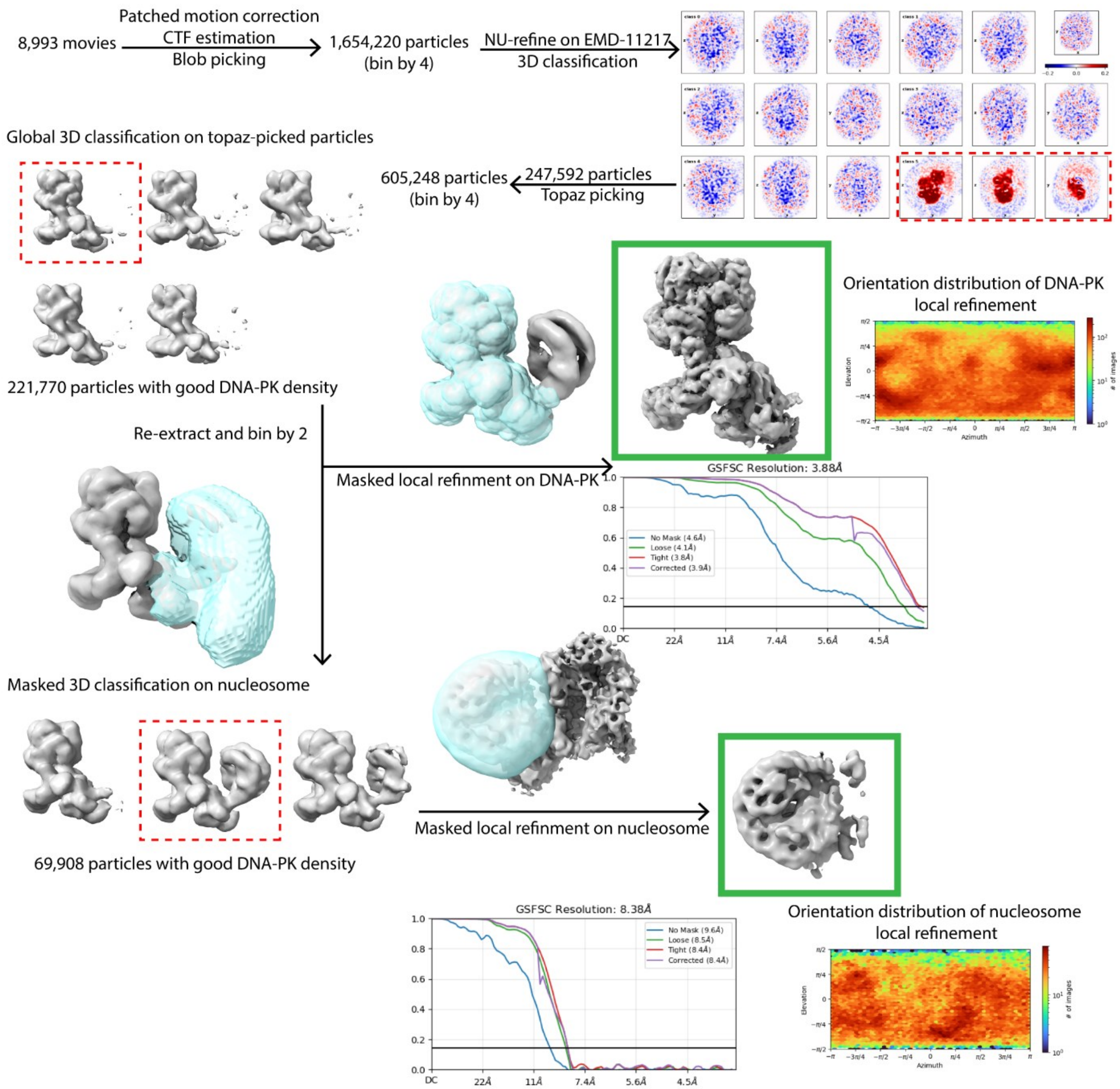

8,993 movies
Patched motion correction
CTF estimation
Blob picking
1,654,220 particles
(bin by 4)
NU-refine on EMD-11217
3D classification
Global 3D classification on topaz-picked particles
605,248 particles
(bin by 4)
247,592 particles
Topaz picking
221,770 particles with good DNA-PK density
Orientation distribution of DNA-PK local refinement
Re-extract and bin by 2
Masked local refinement on DNA-PK
GSFSC Resolution: 3.88Å
No Mask (4.6Å)
Loose (4.1Å)
Tight (3.8Å)
Corrected (3.9Å)
Masked 3D classification on nucleosome
Masked local refinement on nucleosome
69,908 particles with good DNA-PK density
GSFSC Resolution: 8.38Å
No Mask (9.6Å)
Loose (8.5Å)
Tight (8.4Å)
Corrected (8.4Å)
Orientation distribution of nucleosome local refinement

Supplementary Figure 8. Data-processing scheme of the N7-DNA-PK state 1 complex sample.

Flow chart of the cryo-EM data processing procedure. The gold-standard Fourier shell correlation curves (0.143 cut-off) show the final resolutions of the nucleosome and the DNA-PK.

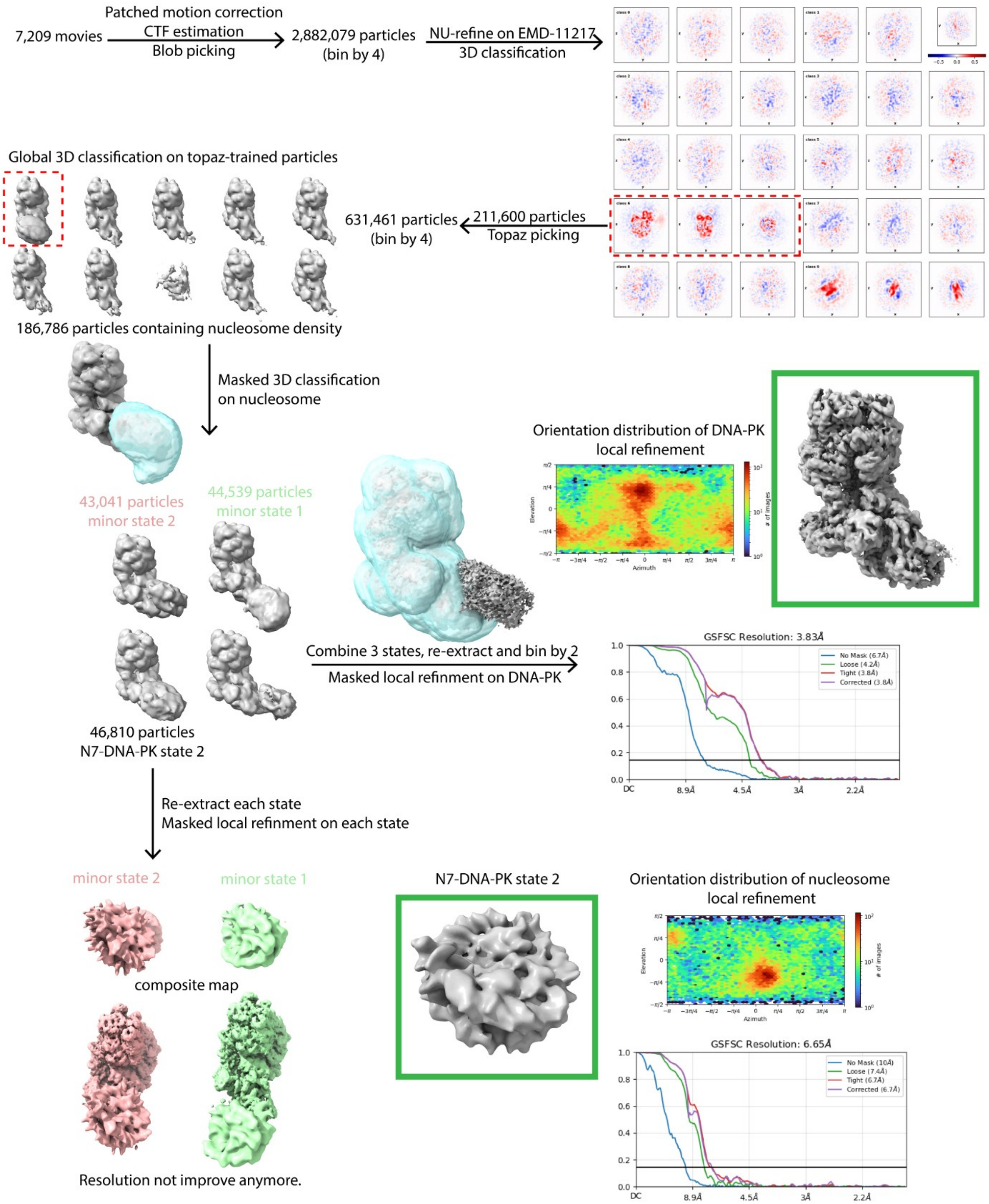

7,209 movies
Patched motion correction
CTF estimation
Blob picking
2,882,079 particles
(bin by 4)
NU-refine on EMD-11217
3D classification
Global 3D classification on topaz-trained particles
631,461 particles
(bin by 4)
211,600 particles
Topaz picking
186,786 particles containing nucleosome density
Masked 3D classification
on nucleosome
43,041 particles
minor state 2
44,539 particles
minor state 1
Orientation distribution of DNA-PK
local refinement
46,810 particles
N7-DNA-PK state 2
Combine 3 states, re-extract and bin by 2
Masked local refinment on DNA-PK
GSFSC Resolution: 3.83Å
No Mask (6.7Å)
Loose (4.2Å)
Tight (3.8Å)
Corrected (3.8Å)
Re-extract each state
Masked local refinment on each state
minor state 2
minor state 1
composite map
Resolution not improve anymore.
N7-DNA-PK state 2
Orientation distribution of nucleosome
local refinement
GSFSC Resolution: 6.65Å
No Mask (10Å)
Loose (7.4Å)
Tight (6.7Å)
Corrected (6.7Å)

Supplementary Figure 9. Data-processing scheme of the N7-DNA-PK state 2 complex sample.

Flow chart of the cryo-EM data processing procedure. The gold-standard Fourier shell correlation curves (0.143 cut-off) show the final resolutions of the nucleosome and the DNA-PK.

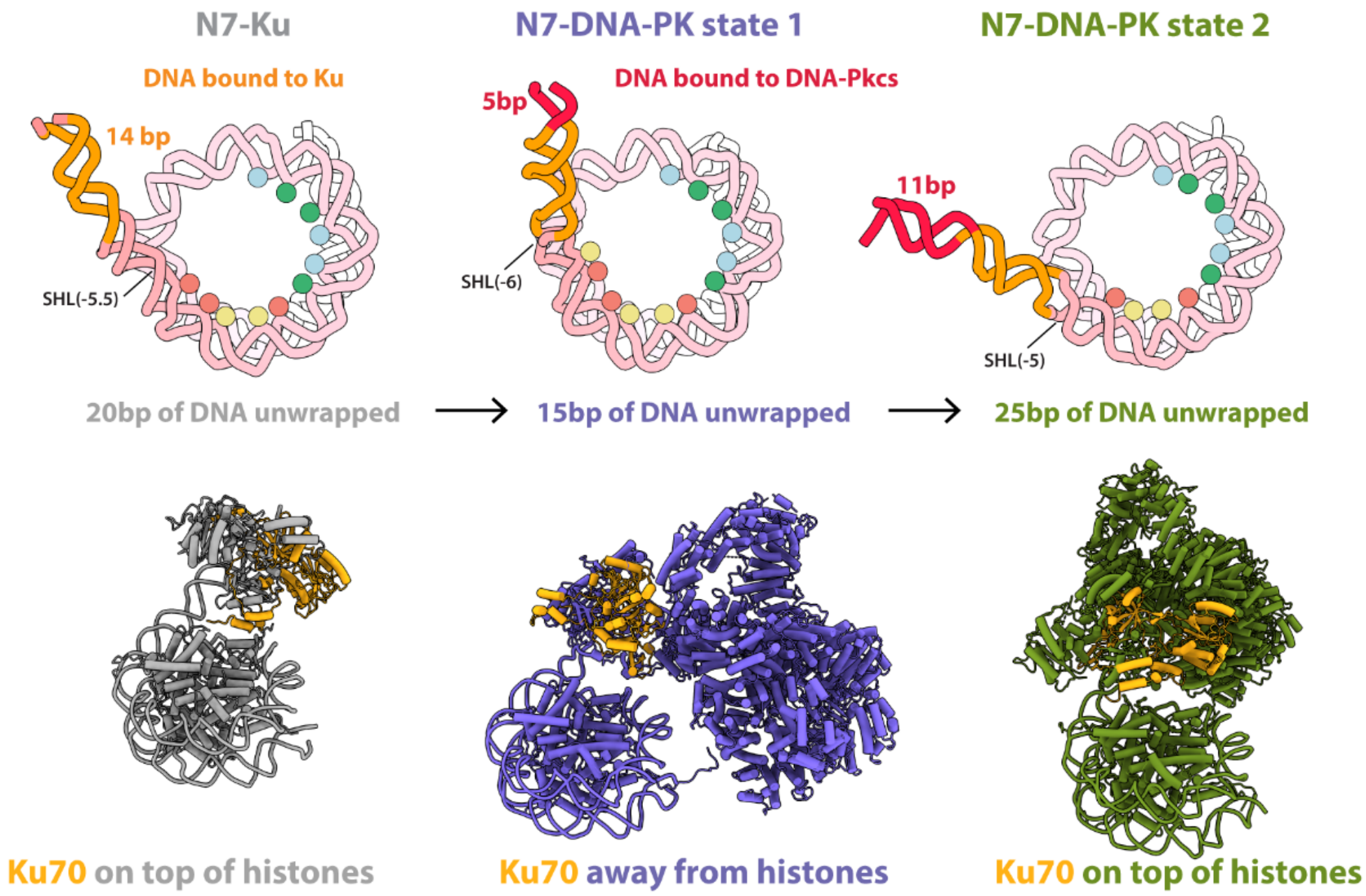

N7-Ku
DNA bound to Ku
14 bp
SHL(-5.5)
20bp of DNA unwrapped
N7-DNA-PK state 1
5bp
DNA bound to DNA-Pkcs
SHL(-6)
15bp of DNA unwrapped
N7-DNA-PK state 2
11bp
SHL(-5)
25bp of DNA unwrapped
Ku70 on top of histones
Ku70 away from histones
Ku70 on top of histones

Supplementary Figure 10. Comparison of Ku70's relative position to nucleosome

Top: Cartoon representations of Ku-bound N7 (left), DNA-PK-bound N7 state 1 (middle), and DNA-PK-bound N7 state 2 (right). Bottom: Structures of Ku-bound N7 (left), DNA-PK-bound N7 state 1 (middle), and DNA-PK-bound N7 state 2 (right). Ku70 is colored yellow to highlight its relative location to histone octamer.

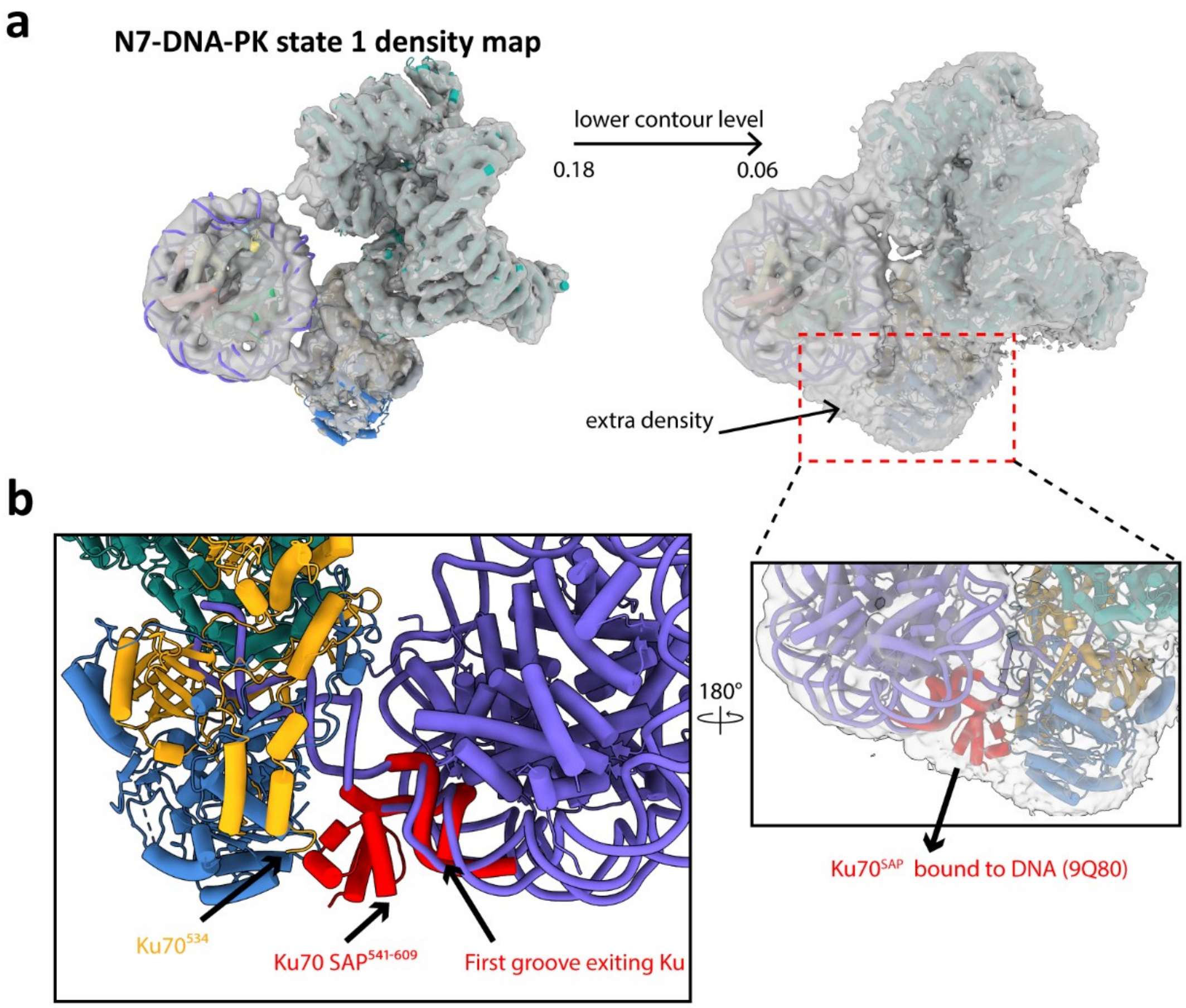

a
N7-DNA-PK state 1 density map
lower contour level
0.18
0.06
extra density
b
180°
Ku70534
Ku70 SAP541-609
First groove exiting Ku
Ku70SAP bound to DNA (9Q80)

Supplementary Figure 11. Close-up view of N7-DNA-PK state 1.

(a) Cryo-EM density map of N7-DNA-PK state 1 shown at high contour (left) and low contour (right) levels, with the atomic model docked into the map. (b) Zoom-in view of the extra density observed between Ku70/80 and the nucleosome. A Ku70 SAP domain (residues 541-609) bound to DNA (9Q80, red) fitted into the model. A different view of the fitted model without the density map are shown on the left, highlighting the position of the Ku70 SAP domain along the major groove of the DNA exiting the Ku channel.

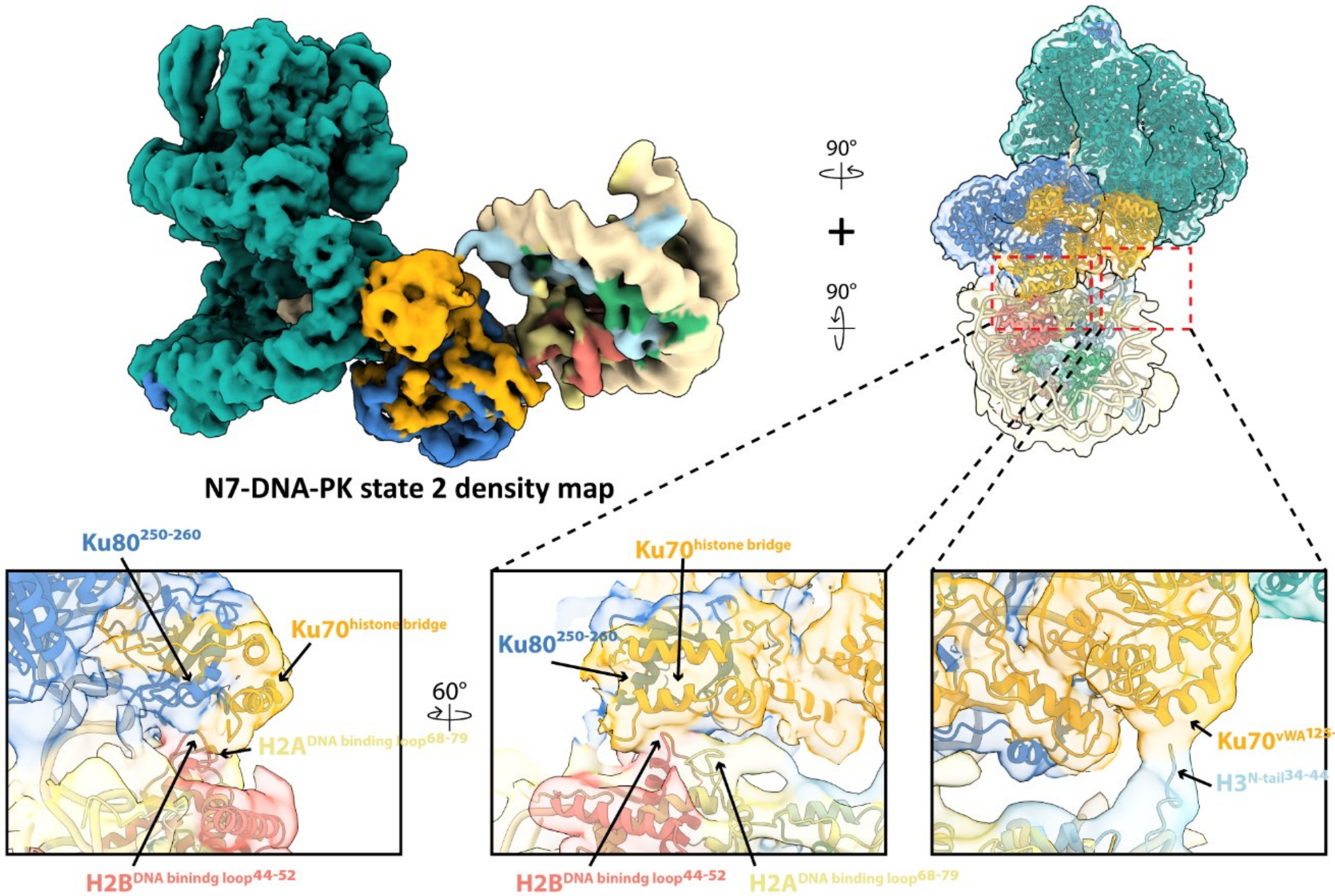

90°
+
90°
N7-DNA-PK state 2 density map
Ku80250-260
Ku70histone bridge
H2ADNA binding loop68-79
H2BDNA binindg loop44-52
60°
Ku70histone bridge
Ku80250-260
H2BDNA binindg loop44-52
H2ADNA binding loop68-79
Ku70vWA125-134
H3N-tail34-44

Supplementary Figure 12. Interactions between nucleosome and DNA-PK in N7-DNA-PK state 2.

Close-up views of the interactions are shown at the bottom. Interactions between Ku70/80 and H2A/H2B are shown on the left and in the middle. Interactions between Ku70 and H3 are shown on the right.

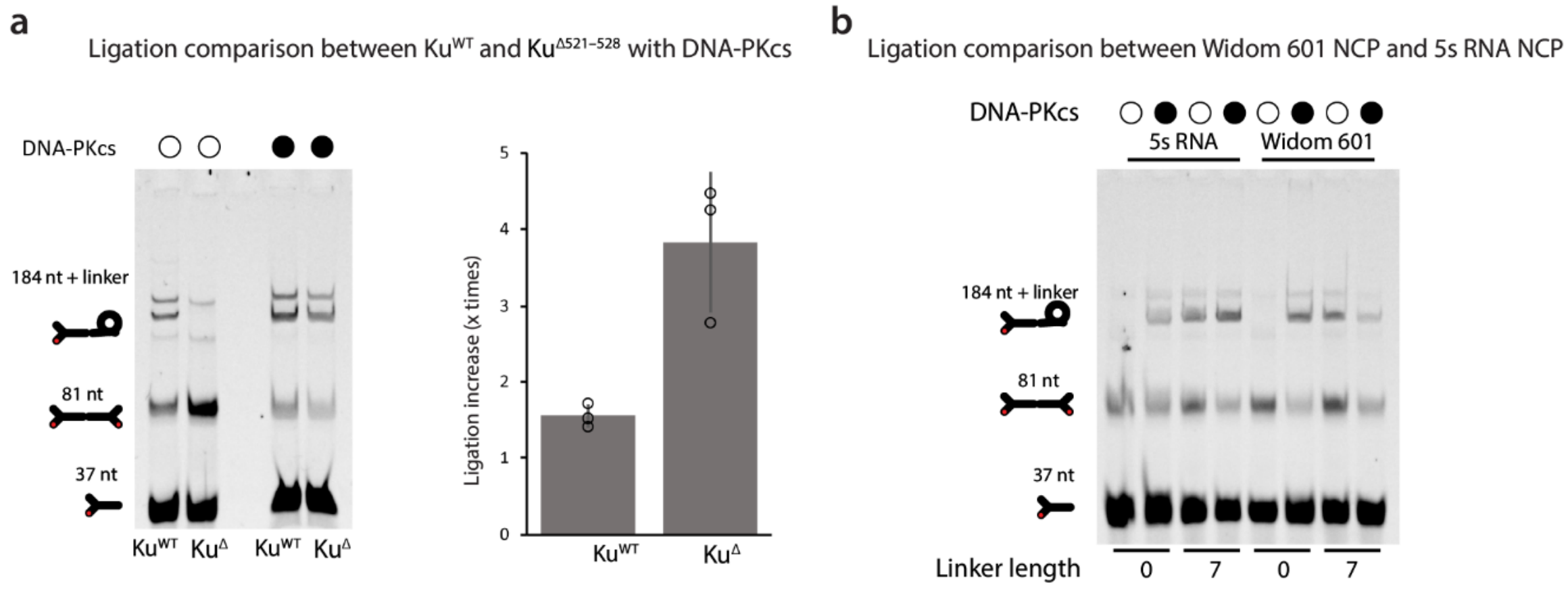

a
Ligation comparison between Ku^WT and Ku^Δ521–528 with DNA-PKcs
DNA-PKcs
184 nt + linker
81 nt
37 nt
Ku^WT
Ku^Δ
Ku^WT
Ku^Δ
Ligation increase (x times)
0
1
2
3
4
5
Ku^WT
Ku^Δ
b
Ligation comparison between Widom 601 NCP and 5s RNA NCP
DNA-PKcs
5s RNA
Widom 601
184 nt + linker
81 nt
37 nt
Linker length
0
7
0
7

Supplementary Figure 13. *In vitro* ligation assays of different conditions

(a) Denaturing TBE-urea gel analysis showing end joining of blunt-end DNA and nucleosomal substrates using KuWT and Ku$^{\Delta 521\text{-}528}$. The condition without DNA-PKcs is the same as Figure 1f. Quantification of the gel is shown on the right. Ligation increase in the condition with DNA-PKcs is calculated by dividing the band intensity of Y30-nucleosome ligation with DNA-PKcs by that of the Y30-nucleosome ligation without DNA-PKcs. All quantifications are measured with three replicates. Individual replicate values are shown. Error bars are presented as mean values +/- SD. P value of each condition from left to right: 0.0155, 0.0129. Source data are provided as a Source Data file. (b) Denaturing TBE-urea gel analysis showing end joining of blunt-end DNA and nucleosomal substrates using Widom 601 nucleosome and 5s RNA nucleosome with linkers of 0 and 7 bp. Source data are provided as a Source Data file.

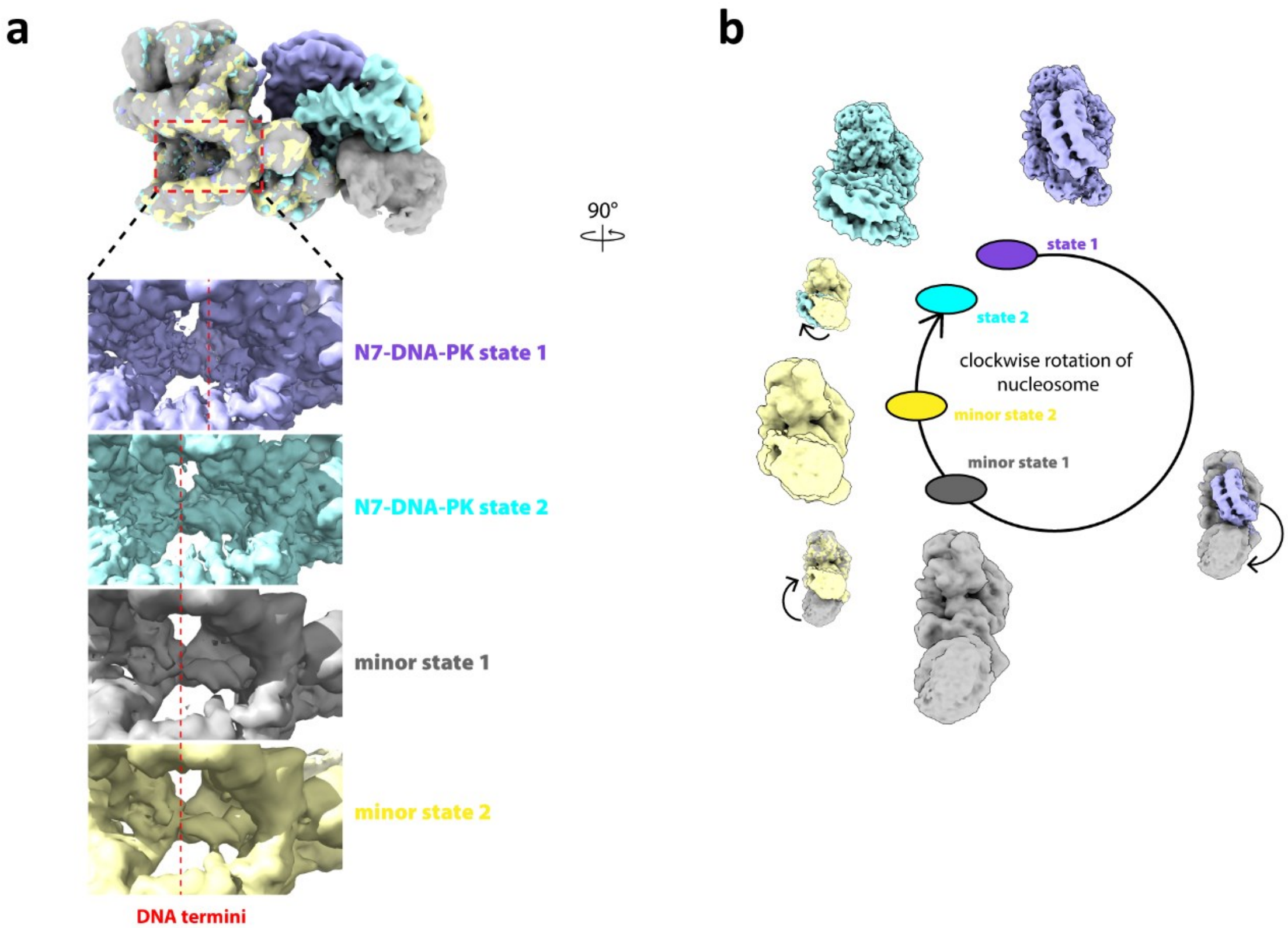

a
N7-DNA-PK state 1
N7-DNA-PK state 2
minor state 1
minor state 2
DNA termini
90°
b
state 1
state 2
clockwise rotation of nucleosome
minor state 2
minor state 1

Supplementary Figure 14. Rotation trajectory of nucleosome from N7-DNA-PK state 1 to state 2.

(a) Superimposition of the four N7-DNA-PK maps resolved in the datasets. The maps are aligned on DNA-PK to show the relative positions of the nucleosome. Close-up views of the DNA-PKcs cradle for each map is shown at the bottom with the DNA termini location marked to show the DNA engagement states of each map. (b) Clockwise rotation of the nucleosome in DNA-PK translocation. Four states are shown in their sequential order. The comparisons and the relative movement of the nucleosome between neighboring states are shown.

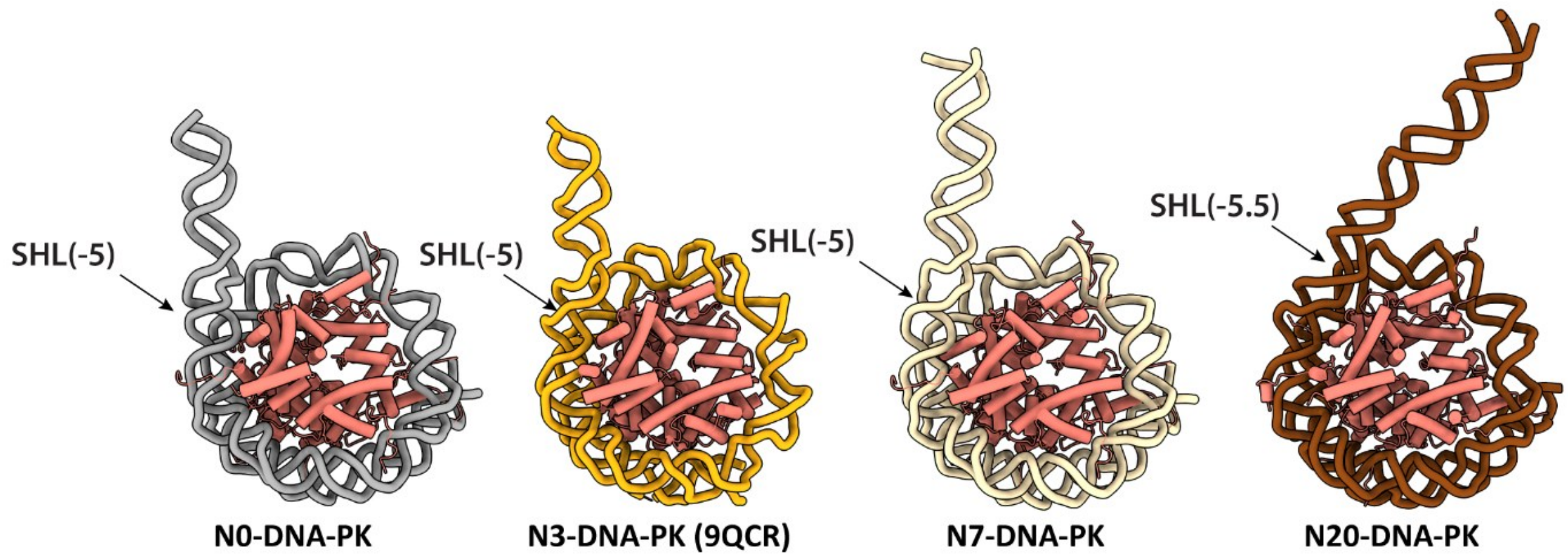
SHL(-5)
SHL(-5)
SHL(-5)
SHL(-5.5)
N0-DNA-PK
N3-DNA-PK (9QCR)
N7-DNA-PK
N20-DNA-PK

Supplementary Figure 15. Nucleosome models from nucleosome-DNA-PK structure of this study and Hall et al., 2025.

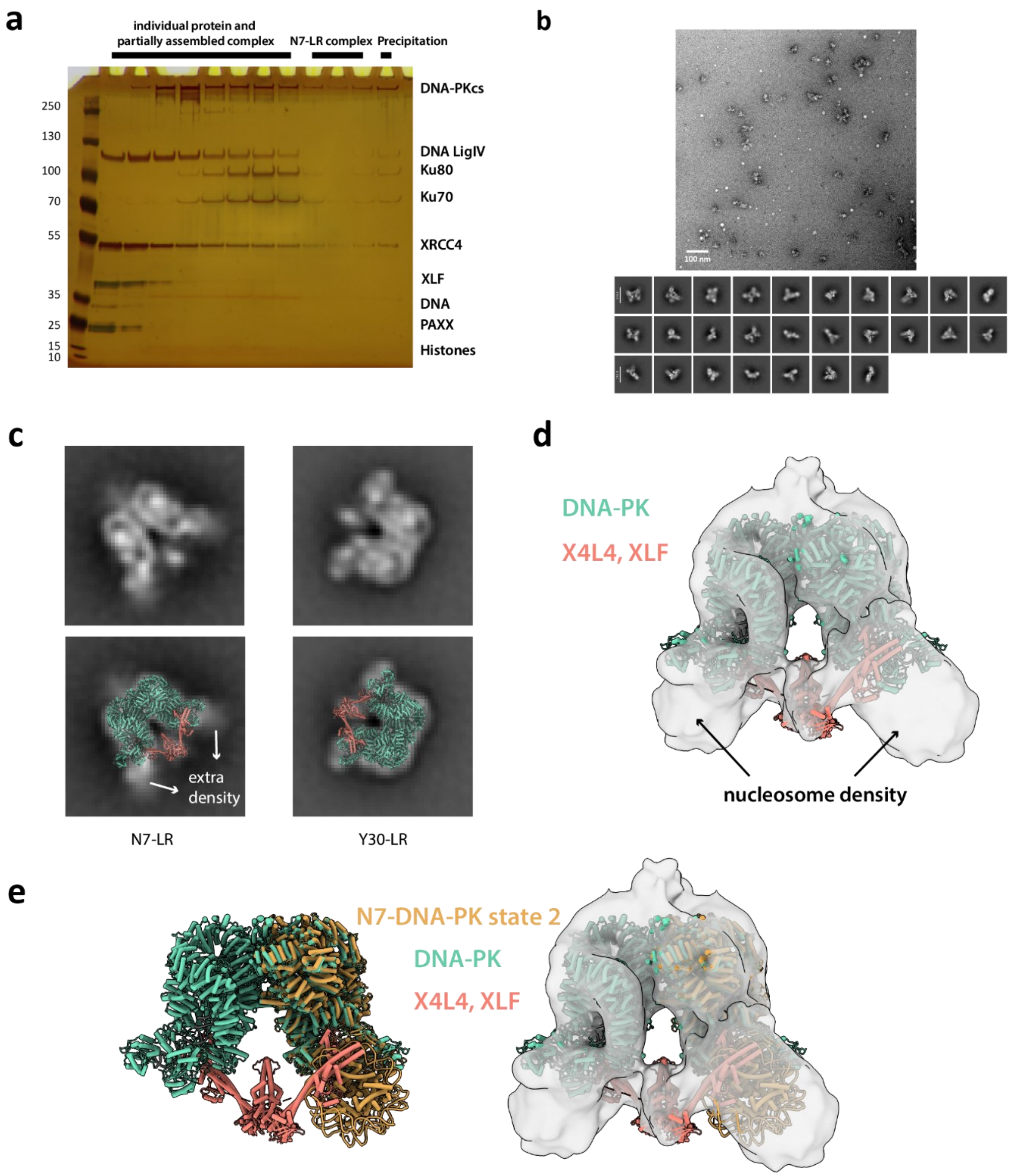
a
individual protein and partially assembled complex
N7-LR complex
Precipitation
250
130
100
70
55
35
25
15
10
DNA-PKcs
DNA LigIV
Ku80
Ku70
XRCC4
XLF
DNA
PAXX
Histones
b
100 nm
c
extra density
N7-LR
Y30-LR
d
DNA-PK
X4L4, XLF
nucleosome density
e
N7-DNA-PK state 2
DNA-PK
X4L4, XLF

Supplementary Figure 16. NS EM analysis of N7-LR complex.

(a) Non-denaturing PAGE analysis of glycerol gradient sample preparations of the N7-LR. Fractions corresponding to individual proteins and partially assembled complex, fully assembled N7-LR complex, and precipitation of the gradient, are labeled on top of the gel. Source data are provided as a Source Data file. (b) Representative negative-stain micrographs and 2D class averages of the N7-LR complex. (c) Front-view comparison of LR complexes assembled on the N7 and Y30 substrates. The previously resolved LR structure (PDB: 7LT3) is docked into 2D averages to highlight the additional density present in the N7-LR complex. (d) Structural model of the LR-Y30 complex docked into the 3D density reconstructed from the NS-EM dataset. (e) Comparison of nucleosome positioning in the N7-DNA-PK state 2 structural model and in the N7-LR density map. Left: the N7-DNA-PK state 2 model docked into one half of the 7LT3 LR model illustrates a steric clash between XRCC4 and the nucleosome. Right: docking the same model into the N7-LR density map reveals a repositioning of the nucleosome that alleviates this clash.

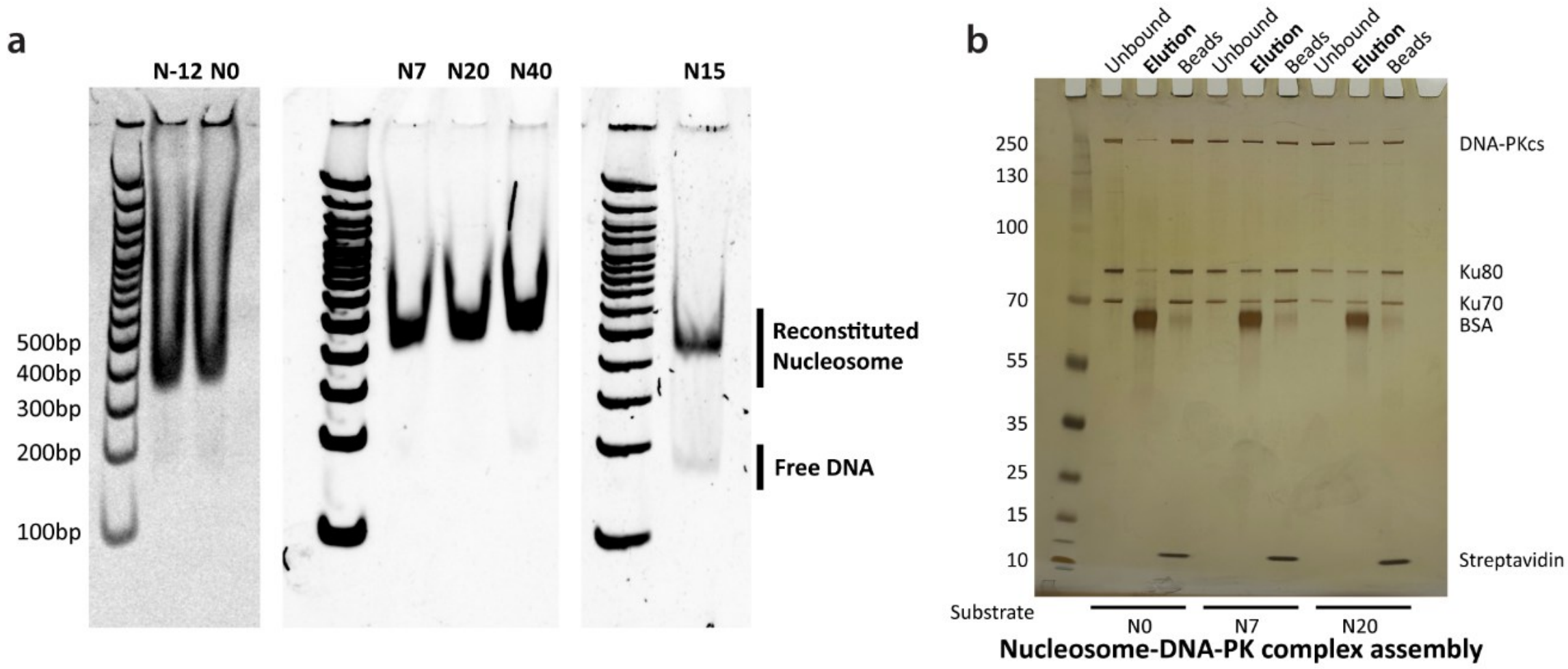

a
N-12 N0
N7 N20 N40
N15
500bp
400bp
300bp
200bp
100bp
Reconstituted Nucleosome
Free DNA
b
Unbound
Elution
Beads
Unbound
Elution
Beads
Unbound
Elution
Beads
250
130
100
70
55
35
25
15
10
DNA-PKcs
Ku80
Ku70
BSA
Streptavidin
Substrate
N0
N7
N20
Nucleosome-DNA-PK complex assembly

Supplementary Figure 17. Gel analysis of complex assembly quality

(a) 6% 0.2× TBE native PAGE gel showing the quality of the nucleosome substrates. Bands corresponding to nucleosome and free DNA are indicated. All samples contain <3% free DNA. Source data are provided as a Source Data file. (b) 4-12% Bis-Tris SDS-PAGE analysis of N0-DNA-PK, N7-DNA-PK, and N20-DNA-PK complex assembly. Elution, unbound protein, and remaining bead lanes are labeled for each substrate. Bands corresponding to the proteins are labeled. Source data are provided as a Source Data file.

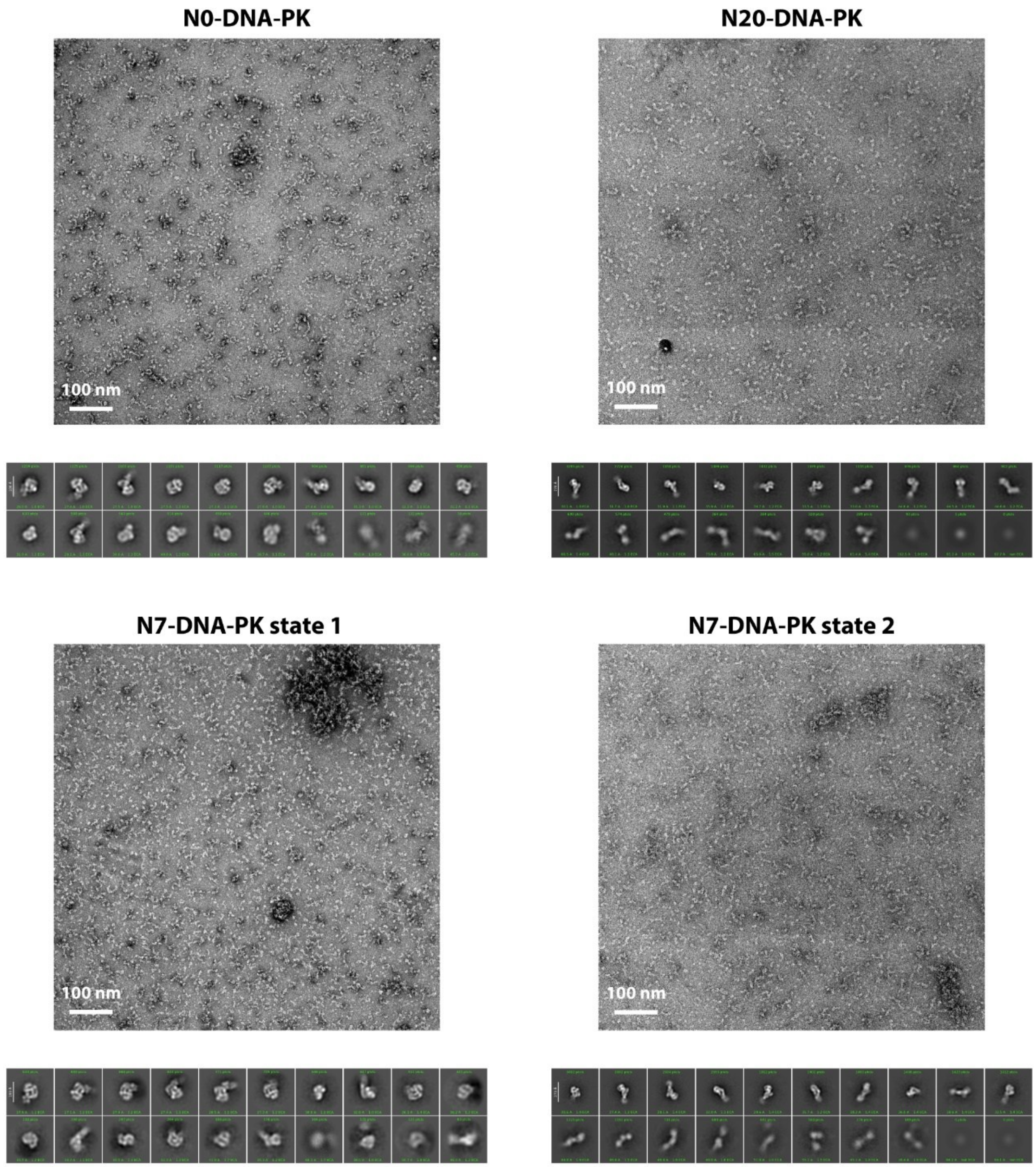
N0-DNA-PK
100 nm
N20-DNA-PK
100 nm
N7-DNA-PK state 1
100 nm
N7-DNA-PK state 2
100 nm

Supplementary Figure 18. NS-EM analysis of complex assembly quality

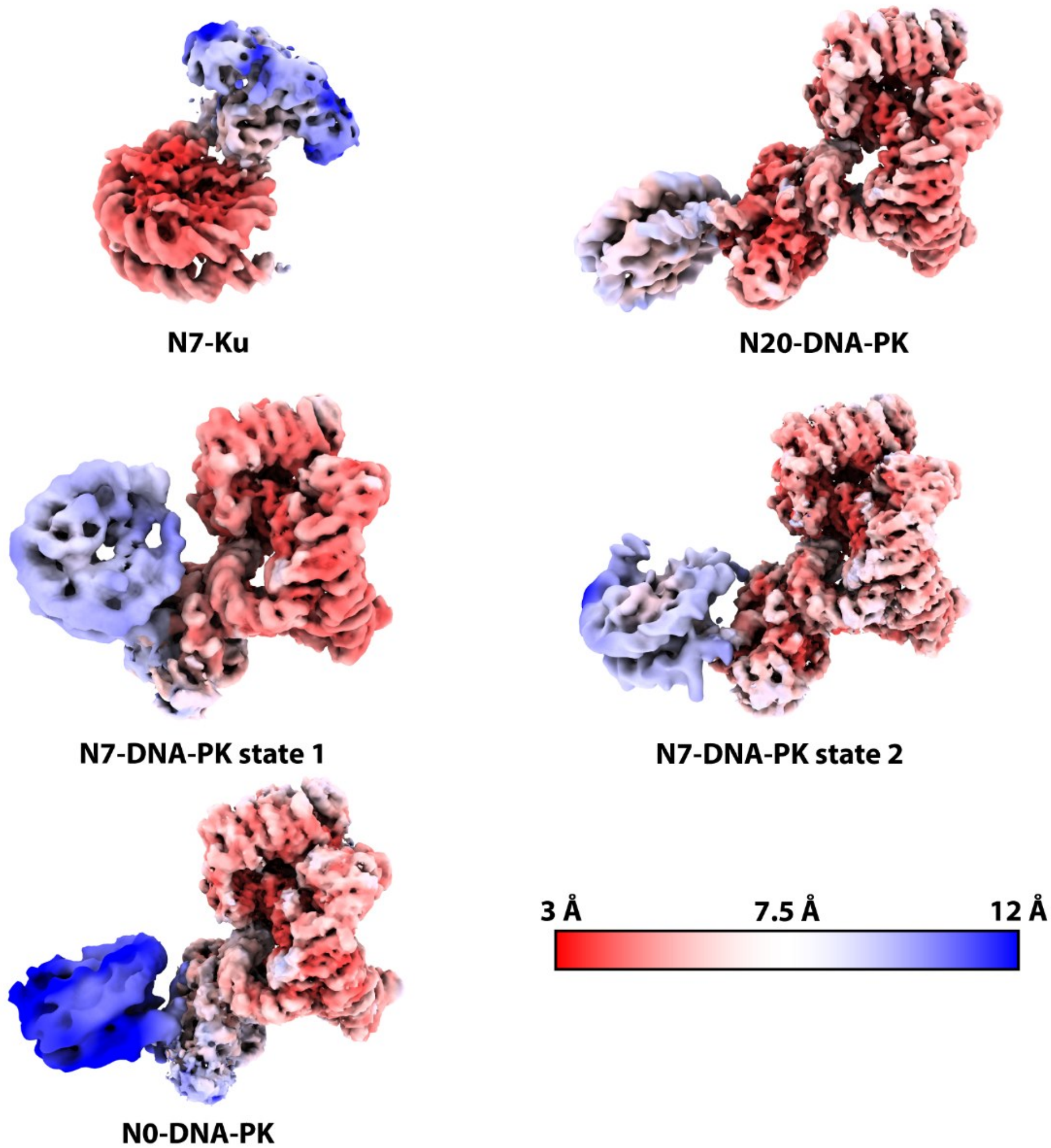
N7-Ku
N20-DNA-PK
N7-DNA-PK state 1
N7-DNA-PK state 2
N0-DNA-PK
3 Å
7.5 Å
12 Å

Supplementary Figure 19. Local resolution maps of all the cryo-EM structures resolved in this study

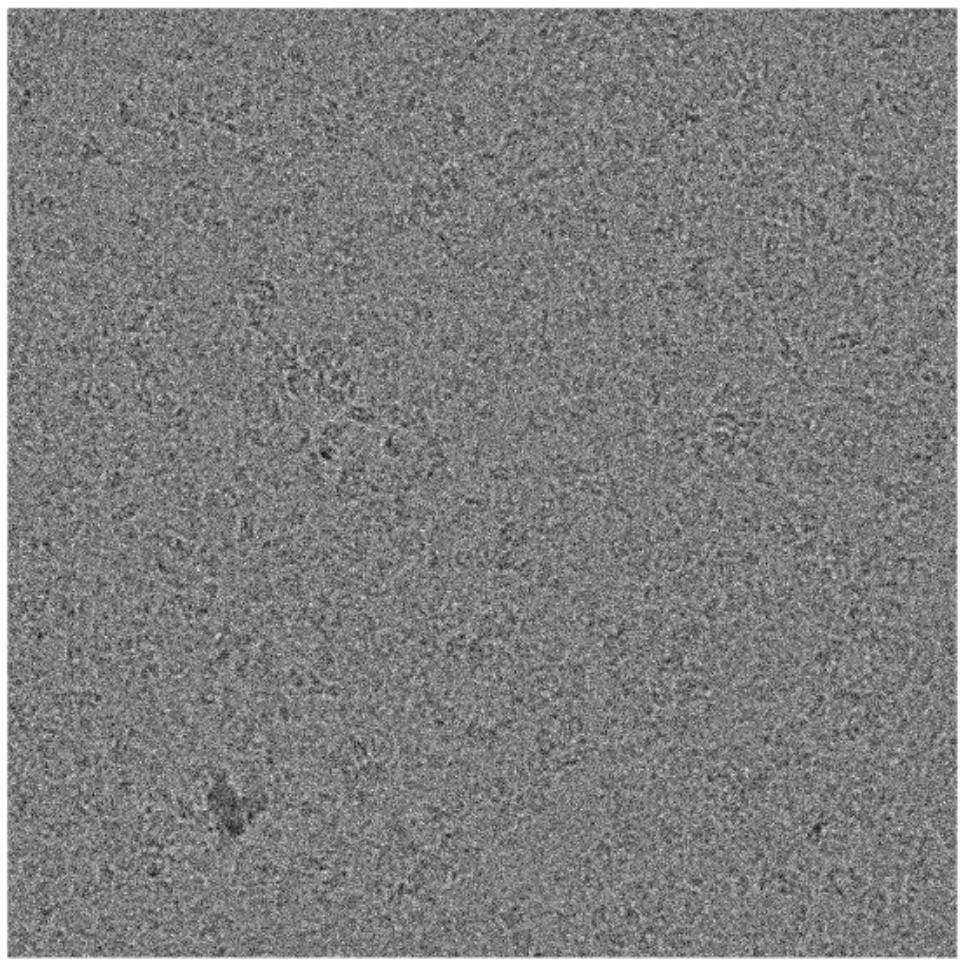

N7-Ku

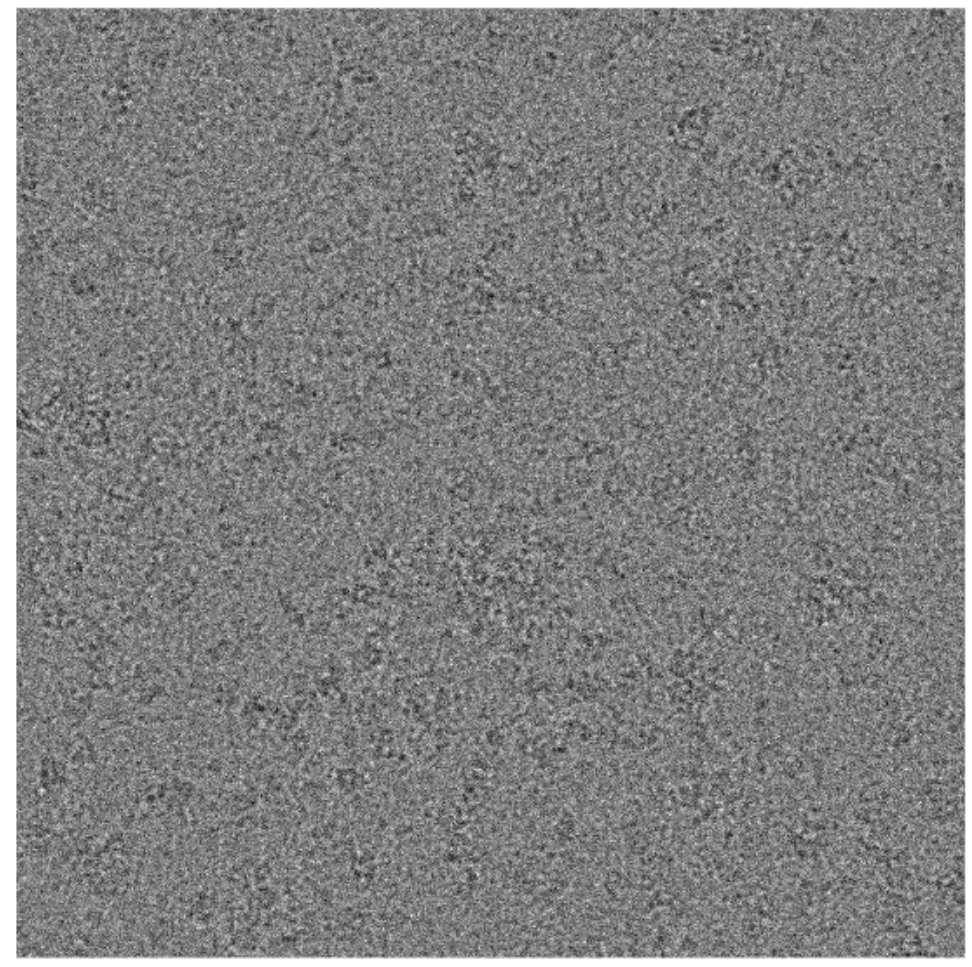

N7-DNA-PK state 1

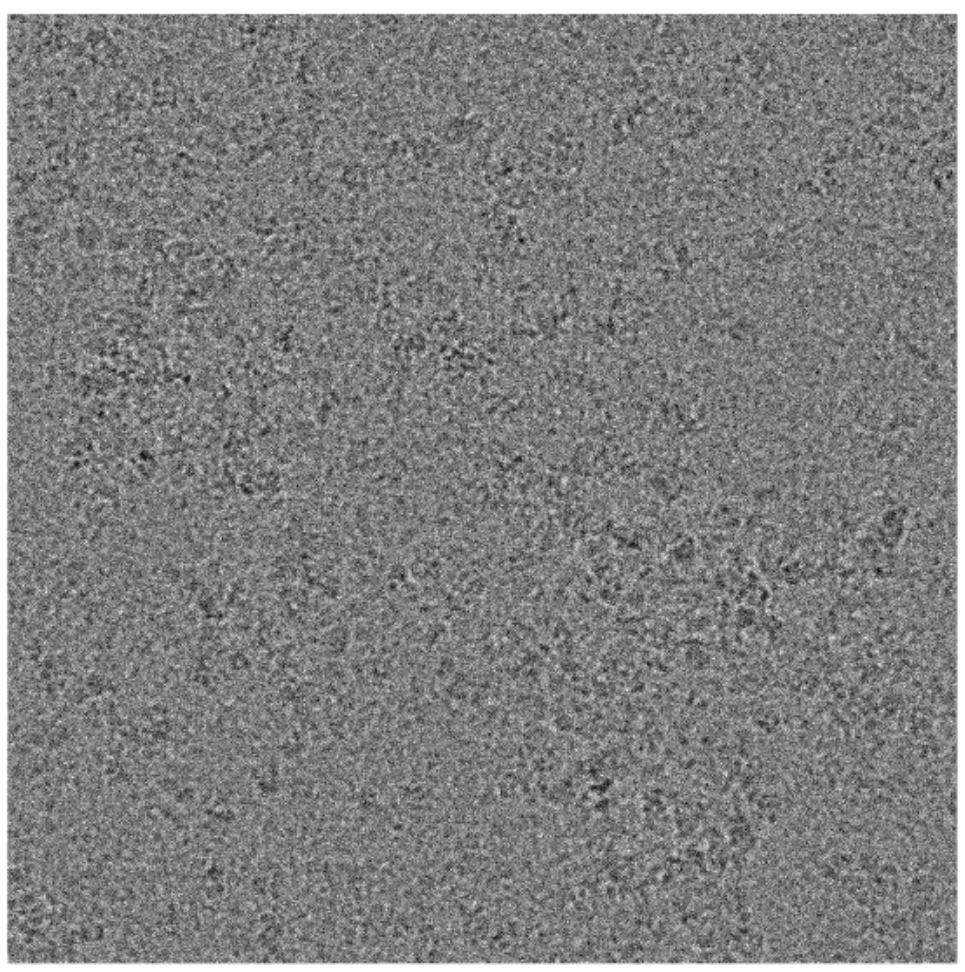

N7-DNA-PK state 2

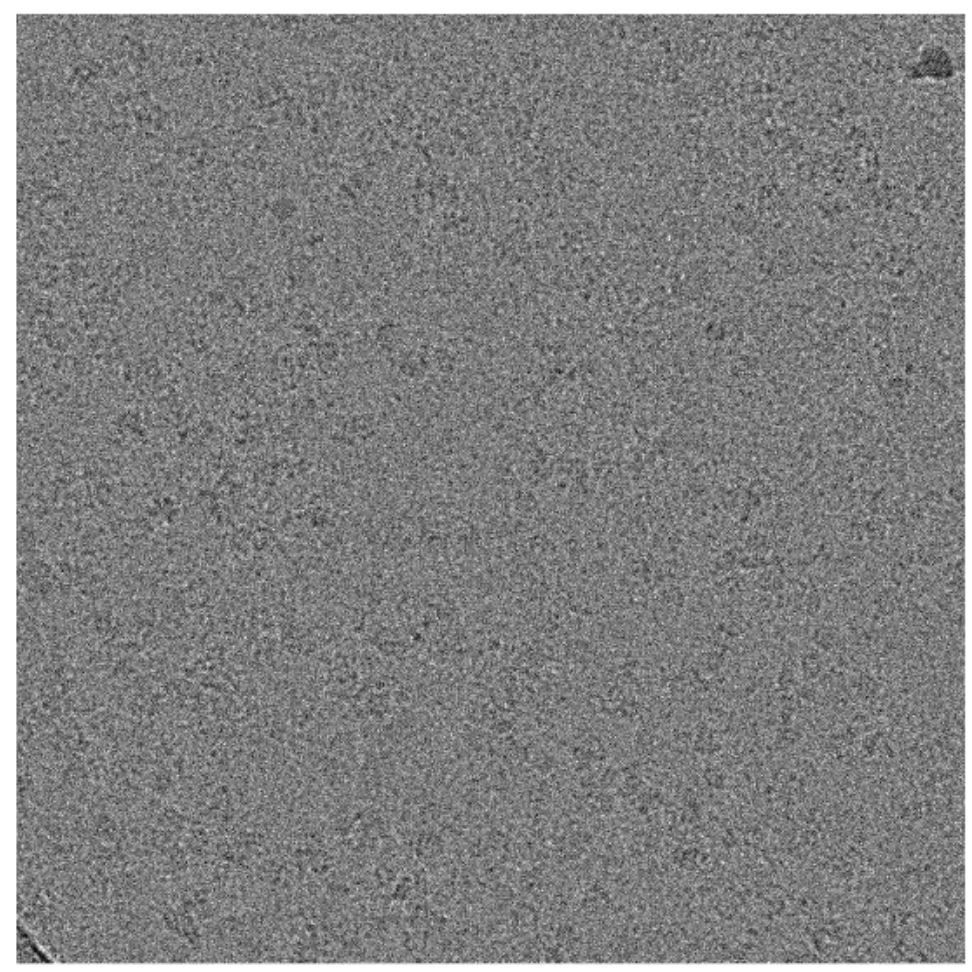

N0-DNA-PK

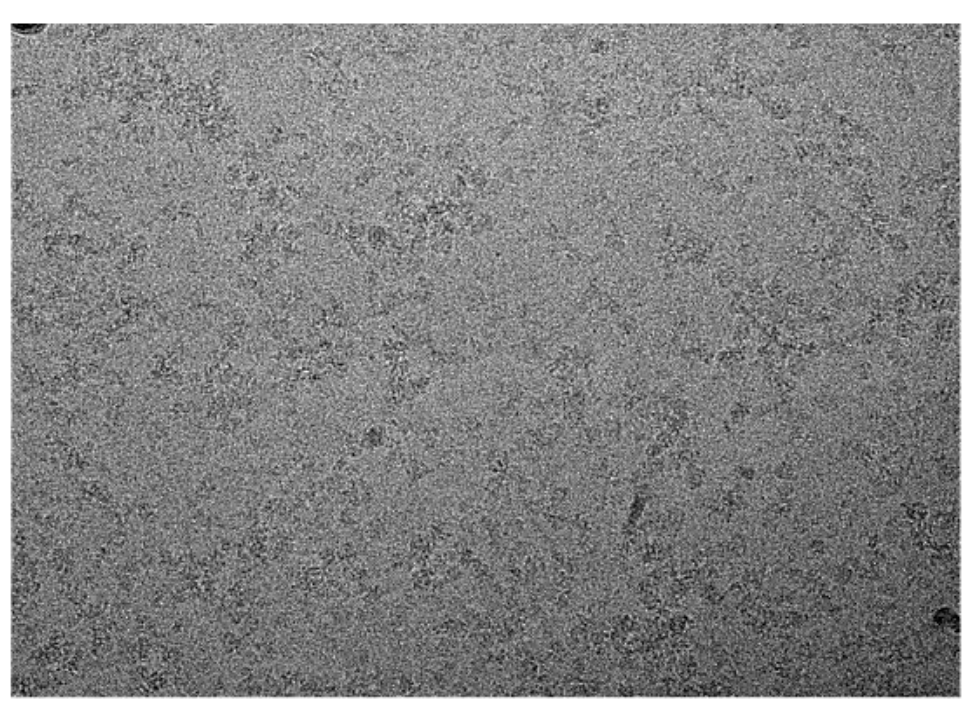

N20-DNA-PK

Supplementary Figure 20. Representative micrographs of each cryo-EM dataset collected in this study

Supplementary Table 1. Cryo-EM data collection and model statistics

	#1 N7-Ku (EMDB-74422) (PDB 9zmm)	#2 N20-DNA-PK (EMDB-74444) (PDB 9znc)	#3 N0-DNA-PK (EMDB-74512) (PDB 9zp0)	#4 N7-DNA-PK state 1 (EMDB-74524) (PDB 9zp9)	#5 N7-DNA-PK state 2 (EMDB-74481) (PDB 9zob)
**Data collection and processing**					
Magnification	120k	30k	120k	120k	120k
Voltage (kV)	300	300	300	300	300
Electron exposure (e–/Å^2)	60	60	60	60	60
Defocus range (μm)	-2.0 to -4.0	-2.0 to -4.0	-2.0 to -4.0	-2.0 to -4.0	-2.0 to -4.0
Pixel size (Å)	0.93	1.0592	0.93	0.93	0.93
Symmetry imposed	C1	C1	C1	C1	C1
Initial particle images (no.)	2,949,075	1,548,937	3,638,917	1,654,220	2,882,079
Final particle images (no.)	25,211	25,710	23,661	69,908	46,810
Map resolution (Å)<br>FSC threshold = 0.5 (Å)	4.42 (nucleosome) 7.27 (Ku70/80)	7.51 (nucleosome), 4.46 (DNA-PK)	11.81 (nucleosome), 4.18 (DNA-PK)	9.73 (nucleosome), 4.33 (DNA-PK)	8.01 (nucleosome), 4.37 (DNA-PK)
Map resolution range (Å)	4.42 to >7	4.46 to >7	4.18 to >10	4.33 to >9	4.37 to >7
**Refinement**					
Initial model used (PDB code)	1JEY, 6R1T	6ZHA, 6R1T	6ZHA, 6R1T	6ZHA, 6R1T	6ZHA, 6R1T
Model resolution (Å)<br>FSC threshold = 0.5 (Å)	4.42 (nucleosome) 7.27 (Ku70/80)	7.51 (nucleosome), 4.46 (DNA-PK)	11.81 (nucleosome), 4.18 (DNA-PK)	9.73 (nucleosome), 4.33 (DNA-PK)	8.01 (nucleosome), 4.37 (DNA-PK)
Model resolution range (Å)	n/a	n/a	n/a	n/a	n/a
Map sharpening *B* factor (Å^2)	-164 (nucleosome), -408 (Ku)	-397 (nucleosome), -85 (DNA-PK)	-63 (DNA-PK)	-1058 (nucleosome), -126 (DNA-PK)	-539 (nucleosome), -35 (DNA-PK)
Model composition					
Non-hydrogen atoms<br>Protein residues<br>Ligands	20,764<br>1,806 (Nucleotide: 308)<br>0	50,638<br>5,477(Nucleotide: 328)<br>0	49,847<br>5,464(Nucleotide: 294)<br>0	50,124<br>5,464(Nucleotide: 308)<br>0	50,144<br>5,467(Nucleotide: 308)<br>0
B factors (Å^2)<br>Protein<br>Ligand	Not estimated	Not estimated	Not estimated	Not estimated	Not estimated
R.m.s. deviations					
Bond lengths (Å)	0.005	0.005	0.004	0.008	0.004
Bond angles (°)	0.931	1.032	1.004	1.032	0.982
Validation					

MolProbity score	2.06	2.10	2.22	2.14	2.14
Clashscore	11.34	9.67	13.48	11.21	11.20
Poor rotamers (%)	0.00	0.39	0.00	0.00	0.00
Ramachandran plot					
Favored (%)	91.86	92.82	88.66	89.22	89.19
Allowed (%)	7.91	7.18	10.82	10.30	10.28
Disallowed (%)	0.22	0.00	0.52	0.48	0.54
