# Supplementary material for: DNA-PK driven nucleosome unwrapping enables NHEJ in chromatin
Source: Nat Commun. 2026 Sep 4;17:10545. doi: 10.1038/s41467-026-77534-9 (PMC13642939; doi:10.1038/s41467-026-77534-9)
Supplement: Supplementary file 2 [file 41467_2026_77534_MOESM2_ESM.pdf]

Description of Additional Supplementary Files

Supplementary Data 1. All oligonucleotides used in the study

Supplementary Video 1. Stepwise model of DNA-PK unwrapping and translocating on N7 nucleosomal DNA
