# Supplementary material for: DNA-PK driven nucleosome unwrapping enables NHEJ in chromatin
Source: Nat Commun. 2026 Sep 4;17:10545. doi: 10.1038/s41467-026-77534-9 (PMC13642939; doi:10.1038/s41467-026-77534-9)
Supplement: Supplementary file 3 [file 41467_2026_77534_MOESM5_ESM.pdf]

nature portfolio

Corresponding author(s): Yuan He

Last updated by author(s): Aug 2, 2026



# Reporting Summary

Nature Portfolio wishes to improve the reproducibility of the work that we publish. This form provides structure for consistency and transparency in reporting. For further information on Nature Portfolio policies, see our Editorial Policies and the Editorial Policy Checklist.

## Statistics

For all statistical analyses, confirm that the following items are present in the figure legend, table legend, main text, or Methods section.

| n/a | Confirmed | |
|---|---|---|
| ☐ | ☒ | The exact sample size (*n*) for each experimental group/condition, given as a discrete number and unit of measurement |
| ☐ | ☒ | A statement on whether measurements were taken from distinct samples or whether the same sample was measured repeatedly |
| ☐ | ☒ | The statistical test(s) used AND whether they are one- or two-sided<br>*Only common tests should be described solely by name; describe more complex techniques in the Methods section.* |
| ☒ | ☐ | A description of all covariates tested |
| ☒ | ☐ | A description of any assumptions or corrections, such as tests of normality and adjustment for multiple comparisons |
| ☐ | ☒ | A full description of the statistical parameters including central tendency (e.g. means) or other basic estimates (e.g. regression coefficient) AND variation (e.g. standard deviation) or associated estimates of uncertainty (e.g. confidence intervals) |
| ☐ | ☒ | For null hypothesis testing, the test statistic (e.g. *F*, *t*, *r*) with confidence intervals, effect sizes, degrees of freedom and *P* value noted<br>*Give P values as exact values whenever suitable.* |
| ☒ | ☐ | For Bayesian analysis, information on the choice of priors and Markov chain Monte Carlo settings |
| ☒ | ☐ | For hierarchical and complex designs, identification of the appropriate level for tests and full reporting of outcomes |
| ☒ | ☐ | Estimates of effect sizes (e.g. Cohen's *d*, Pearson's *r*), indicating how they were calculated |

*Our web collection on statistics for biologists contains articles on many of the points above.*

## Software and code

Policy information about availability of computer code

| | |
|---|---|
| Data collection | Serial EM v3.5 and EPU v3.0 |
| Data analysis | The EM softwares used:cryoSPARC v4.6, Topaz. Structures were built and visualized using WinCoot, Phenix, Chimera v1.13.1, and ChimeraX v1.6. |

For manuscripts utilizing custom algorithms or software that are central to the research but not yet described in published literature, software must be made available to editors and reviewers. We strongly encourage code deposition in a community repository (e.g. GitHub). See the Nature Portfolio guidelines for submitting code & software for further information.

## Data

Policy information about availability of data

All manuscripts must include a data availability statement. This statement should provide the following information, where applicable:

- Accession codes, unique identifiers, or web links for publicly available datasets
- A description of any restrictions on data availability
- For clinical datasets or third party data, please ensure that the statement adheres to our policy

Cryo-EM density maps and model coordinates have been deposited in the Electron Microscopy Data bank (EMDB) and PDB: N7-Ku (EMDB-74422, pdb_00009zmm), N20-DNA-PK (EMDB-74444, pdb_00009znc), N0-DNA-PK (EMDB-74512, pdb_00009zp0), N7-DNA-PK state 1 (EMDB-74524, pdb_00009zp9), N7-DNA-PK state 2

(EMDB-74481, pdb_00009zob). All data needed to evaluate the conclusions in the paper are present in the paper, Supplementary Materials, and/or Source Data. Previously deposited PDBs used in this study include: 1KX5, 1JEY, 6ZHA, 9IGW, 7Z87, 8EZA, 9QCR, 7LT3, 7LTY.

## Research involving human participants, their data, or biological material

Policy information about studies with human participants or human data. See also policy information about sex, gender (identity/presentation), and sexual orientation and race, ethnicity and racism.

| | |
|---|---|
| Reporting on sex and gender | n/a |
| Reporting on race, ethnicity, or other socially relevant groupings | n/a |
| Population characteristics | n/a |
| Recruitment | n/a |
| Ethics oversight | n/a |

Note that full information on the approval of the study protocol must also be provided in the manuscript.

# Field-specific reporting

Please select the one below that is the best fit for your research. If you are not sure, read the appropriate sections before making your selection.

☒ Life sciences ☐ Behavioural & social sciences ☐ Ecological, evolutionary & environmental sciences

For a reference copy of the document with all sections, see nature.com/documents/nr-reporting-summary-flat.pdf

# Life sciences study design

All studies must disclose on these points even when the disclosure is negative.

| | |
|---|---|
| Sample size | No statistical methods were used to predetermine sample size. Sample sizes were chosen based on standard practice in the field and prior experience with similar biochemical and/or structural assays. For structural experiments, sufficient data were collected to achieve convergence and meet established quality criteria (e.g., resolution, map quality, and validation metrics). For biochemical assays, experiments were performed with independent replicates sufficient to ensure reproducibility of the observed effects. |
| Data exclusions | Data in Figure 1a was excluded for the analysis in Figure 1b because Figure 1a was only replicated for the purpose of a clear presentation of the control group and no experimental group was added, therefore it was included for figure illustration but not in quantification analysis. |
| Replication | 3 replicates are performed for all experiments that were quantified |
| Randomization | Samples were not randomly allocated to experimental groups. Experimental conditions were predefined based on the study design. All samples were prepared, processed, and analyzed using standardized protocols, minimizing potential confounding effects. As such, randomization and control of covariates through allocation were not relevant to this study. |
| Blinding | Blinding was not applicable to this study, as the experiments did not involve subjective outcome assessment or group allocation in the conventional sense. All analyses were performed using standardized and reproducible computational and biochemical methods. |

# Reporting for specific materials, systems and methods

We require information from authors about some types of materials, experimental systems and methods used in many studies. Here, indicate whether each material, system or method listed is relevant to your study. If you are not sure if a list item applies to your research, read the appropriate section before selecting a response.

## Materials & experimental systems

| n/a | Involved in the study |
|---|---|
| ☒ | ☐ Antibodies |
| ☐ | ☒ Eukaryotic cell lines |
| ☒ | ☐ Palaeontology and archaeology |
| ☒ | ☐ Animals and other organisms |
| ☒ | ☐ Clinical data |
| ☒ | ☐ Dual use research of concern |
| ☒ | ☐ Plants |

## Methods

| n/a | Involved in the study |
|---|---|
| ☒ | ☐ ChIP-seq |
| ☒ | ☐ Flow cytometry |
| ☒ | ☐ MRI-based neuroimaging |

# Eukaryotic cell lines

Policy information about cell lines and Sex and Gender in Research

| | |
|---|---|
| Cell line source(s) | Human Hela cell and insect cell were obtained from National Cell Culture Center/Biovest International, Wisconsin |
| Authentication | None of the cell lines used were authenticated. |
| Mycoplasma contamination | Human Hela cell and insect cell were not tested for mycoplasma contamination. |
| Commonly misidentified lines (See ICLAC register) | No commonly misidentified lines were used. |

# Plants

| | |
|---|---|
| Seed stocks | n/a |
| Novel plant genotypes | n/a |
| Authentication | n/a |
