# Supplementary material for: DNA-PK driven nucleosome unwrapping enables NHEJ in chromatin
Source: Nat Commun. 2026 Sep 4;17:10545. doi: 10.1038/s41467-026-77534-9 (PMC13642939; doi:10.1038/s41467-026-77534-9)
Supplement: Supplementary file 4 [file 41467_2026_77534_MOESM6_ESM.pdf]

nature portfolio

Peer Review File

DNA-PK driven nucleosome unwrapping enables NHEJ in chromatin

Corresponding Author: Professor Yuan He

This file contains all reviewer reports in order by version, followed by all author rebuttals in order by version.

Version 0:

Reviewer comments:

Reviewer #1

(Remarks to the Author)
Major advances in the structural biology of NHEJ over the last 5-10 years have led to a good understanding of how DNA ends are recognised and bound by the DNA-PK complex and synapsed to enable their joining by LIG4. However, these studies have almost exclusively used naked DNA templates and therefore lack the context of nucleosomes. How the NHEJ machinery interfaces with nucleosomes and chromatinised templates is a fascinating question. While this study is not alone in trying to examine this context (see Hall et al, Nat Comms 2025), it provides a range of important new insights. The manuscript is well written, the experimental data is of high quality and if the following points can be addressed, it is entirely suitable for publication in Nat Comms in my opinion.

Points to address:

1. The study uses the 601 nucleosome positioning sequence throughout. While I completely understand why this approach was used, 601 is a particularly stable sequence for nucleosomes assembly and it is not a given that the conclusions drawn are applicable to nucleosomes assembled on other sequences. It would therefore be good to test the impact of a nucleosome assembled on a random DNA sequence in the ligation assay setup - how does ligation efficiency and the role of DNA-PKcs change in response to different DNA lengths with a random sequence?

2. The changes in ligation efficiency (Fig. 1a and Fig. 3a) are compelling but they should be quantified from triplicates and presented with statistics.

3. The histone:DNA-PK interactions shown in Fig. 4d and Fig. 4e should include the densities observed so that the reader can directly assess the fit of the model.

4. It does not seem possible to conclusively model the SAP domain based on the density in extended data figure 9c. I think parts of the text should be altered to reflect this. For example:

line 236 states 'combined with the putative SAP domain contact on the proximal side.' This should be changed to something like 'and may be further stabilised by the putative SAP domain contact on the proximal side.'

line 294 states 'This partially assembled DNA-PK is stabilised by Ku70 SAP engagements with adjacent DNA'. This should be changed to 'This partially assembled DNA-PK may be stabilised by Ku70 SAP...'

Also, please note that the structure of the SAP domain bound to DNA was shown by Eickhoff et al, Nature 2025. In contrast to the alpha fold model where the SAP binds the major groove, it was positioned over the top of the minor groove, as subsequently also found by Hall et al, Nat Comms 2025. Both of these studies should be referenced in the text around the SAP.

5. It might be worth finding an alternative to 'overhang' to describe the DNA that is not wrapped around the nucleosome (e.g. extension?). It is slightly confusing given that 'overhang' is used to describe the single-stranded DNA resulting from DSB resection.

6. The recent study from the Gellert and Yang labs (Liu et al, Nature 2025) shows an enhancement of NHEJ efficiency by DNA-PKcs on a number of different end structures. I think it would be worth mentioning this in the discussion to reflect that DNA-PKcs could enhance ligation efficiency in different contexts.

Reviewer #2

(Remarks to the Author)
This manuscript by Lu et al., determines the structural basis of early NHEJ in the context of the nucleosome. The authors determine a series of cryo-EM structures of Ku70/80, DNA PKcs, DNA PK, bound to nucleosomes that represent a terminal DNA double-strand break. These structures identify how these NHEJ assemblies "unwrap" nucleosomal DNA to engage the terminal double strand break within the nucleosome, and suggest that DNA PKcs may help partially overcome the nucleosome barrier to NHEJ. The authors also determine an initial low-resolution negative stain EM structure of the LR-complex bridging nucleosomes, providing an initial glimpse into how long-range synapse would occur within chromatin. Ultimately, the authors establish a model of NHEJ within the nucleosome that is generally supported by the structural data, though additional biochemical validation would be useful. These structural insights are timely, and compliments similar work recently published in Nature Communications (PMID: 41444611). While this work will assuredly be well-received in the DNA repair field, I do have several comments/concerns that should be considered and address prior to publication in Nature Communications.

1. From the enzymatic assays in Fig. 1a, the authors conclude that the nucleosome can impede NHEJ-mediated ligation by serving as a barrier for Ku which prevents complex assembly. While these data clearly show that the nucleosome impedes NHEJ-mediated ligation, these data do not directly show that this is due to impaired assembly of the rest of the NHEJ complex. In addition, it's unclear whether these experiments are representative of multiple independent replicate experiments? If so, quantification of the data in Fig. 1a would be helpful to compare the differences between the different reaction conditions.
2. The structures of Ku70 bound to the N7 nucleosome suggest that Ku binding results in unwrapping of the nucleosomal DNA. The authors speculate that the unwrapped nucleosomal DNA is then stabilized by direct interactions between Ku70 and the exposed H2A/H3. This finding is quite interesting. However, it's also possible that Ku70 does not outcompete the histone octamer for the nucleosomal DNA, but rather Ku70 captures the nucleosomal DNA upon spontaneous unwrapping from the histone octamer (PMID: 15580276). The authors may be able to address these two possibilities by determining whether any of the residues in the Ku70 histone binding interface are important for nucleosome binding. Alternatively, I would suggest the authors soften the language in Lines 123-125 and note to the reader the possibility that Ku70 may just capture the nucleosomal DNA upon spontaneous unwrapping.
3. The enzymatic assays in Fig. 3a do not indicate that DNA-PKcs overcomes the nucleosome barrier to Ku-mediated ligation. These experiments indicate that DNA-PKcs stimulates Ku-mediated ligation, but it does recover the activity back to the activity seen for Ku-mediated ligation of free DNA ends (Y30 self-ligation control), in particular for the N0 nucleosome. Similar to Fig. 1a, it's also unclear whether these experiments were performed in replicate and why the authors chose not to quantify the respective reactions to help with the comparisons.
4. Lines 189-191: Ku70 did not fail to assemble on the N0 nucleosome, it just formed a complex ~22% of the time (Extended Data Fig. 1b). Moreover, the number of N0 nucleosomes with DNA-PK bound in the cryo-EM processing workflow in Extended Data Fig. 5 is not all that different than the authors report for Ku70 in the negative stain data found in Extended Data Fig. 1b. It's unclear how the authors come to the stated conclusion.

5. Lines 192-199: It's unclear how the authors can distinguish between DNA-PK physically dissociating the nucleosomal DNA from the histone octamer in these structures and DNA-Pk simply capturing the nucleosomal DNA that spontaneously unwraps from the histone octamer, as noted above for Ku70. This alternative possibility really should be discussed in the absence of additional experiments proving which of these two possibilities is correct. This is a common issue throughout the entire manuscript.

6. The section entitled "DNA-PK progressively unwraps nucleosome in an ATP-independent way" is extremely difficult to follow as a reader, even for a structural biologist. I would encourage the authors to attempt to clarity this section for readers.

7. The authors thoroughly describe different aspects of the DNA-PK-nucleosome structures, pointing out several novel contacts between different components of DNA-PK and the nucleosome. However, it's difficult to interpret whether any of these novel interactions observed are important for NHEJ to occur within the nucleosome without some biochemical validation. Examples include:

• SAP domain - nucleosomal DNA interaction
• FAT domain - H3 interaction
• Ku70 vWA domain – H3 interaction
• Ku80 - H2A loop 68-79 interaction
• Ku70 - H2B loop 44-52 interaction

Given the lack of biochemical validation showing these interactions are important for NHEJ to occur within the nucleosome, the authors really should tone down the interpretation in lines 257-268.

8. The manuscript ends with an attempt to generate an LR-complex bound to the nucleosome to describe how synapsis may occur within chromatin. However, the authors only assess the complex by negative stain EM, showing that DNA-PK can can bridge broken nucleosomal DNA ends. Can the authors comment on why they decided to forego collecting cryo-EM datasets

on the LR-complex to obtain higher resolution insight? This structure could add some interesting insight to the manuscript, though I do appreciate the work involved in collecting additional structures.
9. I strongly encourage the authors to reference work from Amanda Chaplin's Lab on NHEJ within nucleosomes that was recently published in Nature Communications. It seems as if the structures within this manuscript and the structures from the Chaplin Lab have some notable similarities and differences. A few sentences pointing these out in the discussion, and how they fit into the authors model of NHEJ within chromatin would be very helpful for readers.

Minor Comments
1. Lines 67-69: While this is generally a true statement, some chromatin remodeling enzymes involved in the DNA damage response have also been shown to remodel nucleosomes away from DNA breaks (PMID: 38307862).

2. Lines 95-99: This comparison is valid. However, nucleosome-mediated reduction in the enzymatic activities of multiple DNA repair enzymes within the BER and NER pathways have also been described, suggesting this is not an NHEJ-specific phenomenon. This may be worth pointing out.

3. Lines 103-104: The N7 nucleosome was not previously introduced before this point in the manuscript, only the N0 and N-12. It would be nice to introduce that this nucleosome has a 7 bp linker so that the reader doesn't need to go to the oligonucleotide table to find that information.

4. Line 126: The idea of Ku70 as an ATP-independent translocase comes out of nowhere. I'm unsure what this is attempting to convey, but I would suggest the authors try to rephrase this in a way that better described this part of the conclusion.

5. Lines 138-140: This description is a bit confusing as currently written. The authors may want to use the number of bps and the SHL locations to denote where Ku70 is bound vs. where DNA-PKc is bound in relation to the nucleosome to improve clarity for the reader.

6. Lines 146-147: remove the second "binding" from this sentence.

7. Line 324: chromatin instead of chromatins

8. Lines 504-509: If the cryo-EM maps and models have already been deposited into the EMDB and PDB, respectively, the authors should ensure they edit in the correct EMDB and PDB accession numbers within the "Data and Materials Availability" section of the manuscript.

9. Figure 4: Histone H2B DNA binding loop is misspelled.

Reviewer #3

(Remarks to the Author)
Research on the NHEJ pathway, an essential DNA damage repair mechanism, has been conducted primarily using naked DNA. However, because eukaryotic DNA is packaged into chromatin, understanding how NHEJ proceeds on chromatin substrates is highly significant. In this manuscript, the authors use cryo-EM structural analysis to investigate how the key NHEJ factors Ku70/80 and DNA-PKcs interact with the nucleosome. Notably, they focus on how much DNA is peeled away from the nucleosome and propose that the presence of DNA-PKcs promotes this peeling to secure the DNA substrate required for its activity. Their identification of multiple intermediate states during DNA peeling, and the suggestion that DNA-PK rotates around the nucleosome while peeling the DNA, represent novel aspects of the study. On the other hand, structures of the nucleosome–Ku70/80–DNA-PK complex have already been reported, including work showing DNA-PK–dependent changes in nucleosome structure. The manuscript does not cite and compare with the prior work (Hall et al., Nat Commun., 2025).
Further concerns are detailed below.

Major Comments
1. The authors should further discuss the physiological significance of the reported structures. In vivo, chromatin remodelers are activated at DSB sites, and several studies suggest that chromatin disassembly occurs prior to Ku70/80 recruitment (Tripuraneni et al., Genome Res, 2021; Li & Tyler, Elife, 2016). While the authors cite work showing nucleosomes sliding toward DNA ends, those same studies observe subsequent ejection. Thus, it remains unclear whether a nucleosome is stably positioned immediately adjacent to a DSB during NHEJ. Clarifying the conditions under which these nucleosome-bound intermediates might exist in vivo would help readers better understand the physiological context of these structures.
2. It would also be helpful to clarify whether the nucleosome "unwrapping" seen in the structures is actively induced by DNA-PK, or if it is a passive outcome of DNA-PK's high DNA affinity. However, nucleosomal DNA ends are inherently prone to spontaneous breathing and partial unwrapping. The complexes might simply capture transiently exposed DNA, rather than actively peeling DNA from the histone core, as suggested in reference (40). Biochemical experiments comparing the state of the nucleosome before and after DNA-PK binding would help clarify this point and would further strengthen the conclusions of the study.

3. The authors propose direct interactions between the nucleosome, Ku70/80, and DNA-PKcs based on a composite map. However, these contact regions lie at the boundaries of separately refined maps where the resolution gap is big.

Consequently, the evidence for these direct contacts is weak, and the map in several areas appears insufficient to define specific amino acid interactions confidently. To substantiate these claims, the authors should provide further evidence, such as ligation assays using mutant proteins designed to disrupt these interfaces.
4. Further details regarding sample quality are required. Since the state of the DNA ends is central to this study, the authors must provide data (e.g., gel images) showing the purity of the nucleosome preparations and the proportion of naked DNA and hexasomes. In addition, it is unclear how "state 1" and "state 2" samples were prepared for structural analysis, making it difficult to determine the origin of the structural differences observed between the states. The intermediate steps of the GraFix procedure, or any other preparative steps, should also be presented to allow the reader to assess the sample heterogeneity and the validity of the structural conclusions.
5. The authors state that they did not build a model for the N0–DNA-PK complex due to limited resolution (L187–189), yet a model appears to be shown in the figures. Given the limited resolution, as the authors themselves mention, it may be challenging to confidently determine the nucleosome orientation.

6. The authors show that DNA-PK induces nucleosome unwrapping in an ATP-independent manner. The authors should clarify precisely where the required energy originates. I also encourage the authors to estimate the energy required for nucleosome unwrapping and to compare it quantitatively with the proposed energy source.

7. The authors use the 601 sequence for nucleosome assembly. However, the 601 sequence forms relatively rigid nucleosomes, and the total stability of these nucleosomes is higher than that of native genomic sequences. I recommend using nucleosomes assembled with native DNA sequences (e.g., -satellite or LIN28) to investigate how DNA-PK and Ku overcome these nucleosomal barriers.

8. In Figure 5, steps 5 and 6 appear to be speculative, as no direct evidence supporting this model is presented. Although LR complex assembly has been demonstrated using naked DNA in previous studies, the authors have not provided data supporting the proposed model in the nucleosomal context. Additional experimental evidence would be necessary to substantiate this model.

Minor Comments
1. In all structural analyses, information on local resolution and angular distribution is not provided.
2. In Extended Data Fig. 1A, 11A, scale bars are missing.
3. In Fig. 4a and 4b, the red arrows are hard to distinguish, making it unclear which regions are highlighted. Using clearer colors or zoomed-in views would improve visibility.
4. In Figure 4c, the current H3 tail model appears to be placed in density corresponding to the DNA end–DNA-PK interface. H3 tail density should instead emerge from between the two DNA gyres.
5. In Figure 4e, although the map shows interactions between Ku70–Ku80 and Ku80–H2A/H2B, the direct interaction between Ku70 and H2A/H2B is not visible even at higher contour levels. Clearer map evidence should be provided to support this interaction.
6. In Extended Data Fig. 9a, the threshold values should be indicated for reproducibility. In addition, some density is also present in state 2 at lower contour levels; therefore, the authors should compare map with map rather than map with model. It is also unclear where the model shown originates, as it is not included among the provided PDB files.
7. In Extended Data Fig. 10b, the panel is difficult to interpret. The enlarged region should be clearly marked on the full map, and DNA density should be color-coded for clarity. In addition, because the composite map was generated by combining three states, the matching DNA length is expected and should be acknowledged.
8. In Supplementary Table 2, the model resolution is reported using the FSC 0.143 threshold, but it should be estimated using the 0.5 criterion.

Version 1:

Reviewer comments:

Reviewer #1

(Remarks to the Author)
The authors have addressed my major concerns - after some remaining minor points are addressed, the manuscript is suitable for publication in my opinion.

Minor points:

1. including the 5S RNA sequence is a good addition. However, writing in the text that there is 'no significant difference' between 601 and 5S nucleosomes is not justified without a statistical test for significance, which has not been performed as far as I can see.

2. The quantifications are an improvement. However, as above no stats have been performed to test for statistically significant differences between the relevant samples. These should be included prior to publication

Other than that, I commend the authors on a nice study.

Reviewer #2

(Remarks to the Author)
The authors have thoroughly addressed my original comments/concerns. This manuscript will be extremely valuable to the DNA repair field, and I completely support the publication of this manuscript in Nature Communications.

Minor Comment
1. Lines 569-574: It appears the PDB and EMDB accession codes are still missing from the revised manuscript.

Reviewer #3

(Remarks to the Author)
The authors have addressed the previous comments well, and the manuscript has been substantially improved. In particular, the comparison with the recent study by Hall et al. is well presented, and the identification of multiple intermediate states during DNA-PK assembly on the nucleosome further highlights the novelty of this work. The additional biochemical experiments also strengthen the authors' conclusions.
The remaining comments are minor:
1. Referring to the Ku70 Δ521–528 mutant as "KuMT" is confusing because it is easily mistaken for "KuWT" and does not indicate that the mutation is in Ku70. A more descriptive name, such as "Ku70 Δ521–528", would improve clarity.
2. Several expressions, including "Advancement of DNA-PKcs" in Fig. 5b and terms such as "DNA-PK unwraps," "DNA-PKcs advances," and "translocates" throughout the manuscript, still imply that DNA-PK actively peels DNA away from the histone octamer. The wording should be revised to better reflect the current model.
3. The statement "DNA-PKcs can compensate for weakened Ku-histone interactions" (lines 276–277) appears stronger than the data support and should be replaced with a more conservative statement, such as "DNA-PKcs rescues the ligation defect."

Open Access This Peer Review File is licensed under a Creative Commons Attribution 4.0 International License, which permits use, sharing, adaptation, distribution and reproduction in any medium or format, as long as you give appropriate credit to the original author(s) and the source, provide a link to the Creative Commons license, and indicate if changes were made.
In cases where reviewers are anonymous, credit should be given to 'Anonymous Referee' and the source.
The images or other third party material in this Peer Review File are included in the article’s Creative Commons license, unless indicated otherwise in a credit line to the material. If material is not included in the article’s Creative Commons license and your intended use is not permitted by statutory regulation or exceeds the permitted use, you will need to obtain permission directly from the copyright holder.
To view a copy of this license, visit https://creativecommons.org/licenses/by/4.0/

Authors' Response

We thank all reviewers for their thorough reading of our manuscript and detailed feedback. In response to their comments, we have performed 3 new experiments in addition to new analysis of our data which we include in Figures 2b, 2f, 3b, Supplementary Fig. 1a, 3, 7, 10, 12, 13, 15, 16a, 17, 18, and 19

We have also made substantial changes to the body of the manuscript to incorporate reviewers' suggestions as well as incorporate findings published contemporaneously with our submission. Manuscript line numbers are referenced throughout the responses below.

**Figure**	**Changes**
Fig. 1	b – quantification of 1a over three replicates<br>f – ligation assay with KuMT and its quantification
Fig. 2	f – the correct activated DNA-PK (7z87) model used
Fig. 3	b – quantification of 3a over three replicates
Fig. 4	a, b – DNA-PKcs fragments are re-labeled for clarity<br>c – corrected PDB model are now used
Fig. 5	No change
Supplementary Fig. 1	a – silver-stain gel showing N0-Ku and N7-Ku complex assembly<br>b – scale bar is added
Supplementary Fig. 2, 4, 6, 8, 9	Orientation distribution added for each local refinement
Supplementary Fig. 3	New figure to show comparison between nucleosome-Ku structures of this study and Hall et al.
Supplementary Fig. 5	No change
Supplementary Fig. 7	c, d – new panels to show comparison between nucleosome-DNA-PK structures of this study and Hall et al.
Supplementary Fig. 10	New figure to show Ku70's relative position to nucleosome in different structures
Supplementary Fig. 11	The original panel a is removed.<br>b – updated model of Ku70 SAP bound to DNA is used to replace Ku70 SAP AF3 prediction
Supplementary Fig. 12	New figure to show interactions in N7-DNA-PK state 2
Supplementary Fig. 13	New figure to show ligation assay results of:<br>a – ligation assay comparing KuMT and KuWT, and its quantification<br>b – ligation assay comparing 5s RNA and Widom 601 nucleosome
Supplementary Fig. 14	Panels are rearranged for clarity
Supplementary Fig. 15	New figure to show comparison between structures of nucleosomes in all nucleosome-DNA-PK complex resolved in this study and Hall et al.

Supplementary Fig. 16	a – silver stain showing the complex assembly quality from glycerol gradient<br>b – scale bar is added
Supplementary Fig. 17	New figure to show gel assessment of complex assembly
Supplementary Fig. 18	New figure to show NS-EM assessment of complex assembly
Supplementary Fig. 19	New figure to show local resolution of cryo-EM maps

Reviewer #1 (Remarks to the Author):

Major advances in the structural biology of NHEJ over the last 5-10 years have led to a good understanding of how DNA ends are recognised and bound by the DNA-PK complex and synapsed to enable their joining by LIG4. However, these studies have almost exclusively used naked DNA templates and therefore lack the context of nucleosomes. How the NHEJ machinery interfaces with nucleosomes and chromatinised templates is a fascinating question. While this study is not alone in trying to examine this context (see Hall et al, Nat Comms 2025), it provides a range of important new insights. The manuscript is well written, the experimental data is of high quality and if the following points can be addressed, it is entirely suitable for publication in Nat Comms in my opinion.

We thank the reviewer for their positive comments regarding our manuscript. We made substantial additions to our manuscript to incorporate the results from Hall, et al 2025, which was published after our submission. We have also included quantification for the ligation assays and clarified the confusing text in our manuscript as the reviewer suggested.

Points to address:

1. The study uses the 601 nucleosome positioning sequence throughout. While I completely understand why this approach was used, 601 is a particularly stable sequence for nucleosomes assembly and it is not a given that the conclusions drawn are applicable to nucleosomes assembled on other sequences. It would therefore be good to test the impact of a nucleosome assembled on a random DNA sequence in the ligation assay setup - how does ligation efficiency and the role of DNA-PKcs change in response to different DNA lengths with a random sequence?

We appreciate the reviewer for suggesting the replacement of Widom 601 nucleosome positioning sequence to a more native nucleosome positioning sequence. As

suggested, we replace the Widom 601 sequence with the 5S RNA nucleosome. 5S RNA nucleosome contains a native endogenous sequence and has a lower affinity toward histone octamer than the artificial Widom 601 sequence. It has been widely used in different chromatin-related *in vitro* studies.

To test the ligation efficiency on 5S RNA nucleosomes, we reconstituted the N0 and N7 nucleosome using 5S RNA sequence, as they appear to have the most drastic ligation difference on Widom 601 sequence. The ligation result is very similar compared to Widom 601 nucleosomes.

The ligation result is shown in Supplementary Fig. 13b and the text corresponding to the results is at line 298-307:

To further examine the contribution of spontaneous DNA breathing, we replaced the Widom 601 nucleosome-positioning sequence with the 5S rRNA nucleosome-positioning sequence, a native sequence that is reported to have stronger DNA breathing effect and more closely reflects physiological histone-DNA affinity. Using an in vitro ligation assay, we observed no significant difference in ligation efficiency between Widom 601 and 5S rRNA nucleosomes for either N0 or N7 substrates (Supplementary Fig. 13b). In conclusion, the structural and biochemical results we present here suggest that the interaction between nucleosome and Ku/DNA-PK is very dynamic. Nucleosomal DNA is captured by Ku/DNA-PK through spontaneous end breathing and allowed to rewrap around histone octamer whenever histones are exposed without contacting DNA-PK.

2. The changes in ligation efficiency (Fig. 1a and Fig. 3a) are compelling but they should be quantified from triplicates and presented with statistics.

We thank the reviewer for the suggestion to include quantification on the ligation assays. We have made the following quantifications from triplicates and include them in Fig. 1b and 3b.

In brief, the ligation efficiency in each condition is calculated by dividing the intensity of the bands corresponding to the nucleosomal ligation to the intensity of the bands corresponding to internal Y30 DNA ligation. For comparisons between the presence and absence of DNA-PKcs, the effect of DNA-PKcs is calculated by dividing the ligation efficiency without DNA-PKcs to the efficiency with DNA-PKcs.

3. The histone:DNA-PK interactions shown in Fig. 4d and Fig. 4e should include the densities observed so that the reader can directly assess the fit of the model.

We thank the reviewer for raising this point. To address this, we added a new Supplementary Fig. 12 to show the densities with the fitted model.

4. It does not seem possible to conclusively model the SAP domain based on the density in extended data figure 9c. I think parts of the text should be altered to reflect this. For example:

line 236 states 'combined with the putative SAP domain contact on the proximal side.' This should be changed to something like 'and may be further stabilised by the putative SAP domain contact on the proximal side.'

line 294 states 'This partially assembled DNA-PK is stabilised by Ku70 SAP engagements with adjacent DNA'. This should be changed to 'This partially assembled DNA-PK may be stabilised by Ku70 SAP...'

Also, please note that the structure of the SAP domain bound to DNA was shown by Eickhoff et al, Nature 2025. In contrast to the alpha fold model where the SAP binds the major groove, it was positioned over the top of the minor groove, as subsequently also found by Hall et al, Nat Comms 2025. Both of these studies should be referenced in the text around the SAP.

We thank the reviewer for raising this point. We agree that our language in the original manuscript was too strong regarding the Ku70 SAP domain and tuned down the tone at line 256-262:

Additional unassigned density appears near the C-terminus of the Ku70. Studies have suggested that SAP domain participates in Ku binding to DNA by engaging the adjacent DNA and potentially limiting Ku's lateral movement (Supplementary Fig. 11). The position of this extra density appears at the first major groove of the exiting DNA from Ku70/80, consistent with the Ku70 SAP resolved in the previous structure. Together, these observations suggest potential interactions that might trap DNA-PK in its intermediate state on the N7 substrate.

And line 334~335:

This partially assembled DNA-PK may be stabilized by Ku70 SAP engagements with adjacent DNA and by a contact between the distal H3 tail and the DNA-PKcs FAT domain.

We made additional changes in the Supplementary Fig. 11 by replacing the model from AF with the SAP domain bound to nucleosomal DNA from structure resolved in Hall et

al. (PDB: 9Q80). The position of the density aligns with the position of the SAP domain when the two models are superimposed on Ku structure, supporting that the extra density might belong to SAP.

We also included references for Eickhoff et al, Nature 2025 and Hall et al, Nat Comms 2025 in the text around Ku70 SAP.

5. It might be worth finding an alternative to 'overhang' to describe the DNA that is not wrapped around the nucleosome (e.g. extension?). It is slightly confusing given that 'overhang' is used to describe the single-stranded DNA resulting from DSB resection.

We agree with the reviewer that overhang can cause confusion as it is also used to describes ssDNA resection. Therefore, we change ‘overhang’ to ‘free DNA linker’, or ‘linker’ for short.

6. The recent study from the Gellert and Yang labs (Liu et al, Nature 2025) shows an enhancement of NHEJ efficiency by DNA-PKcs on a number of different end structures. I think it would be worth mentioning this in the discussion to reflect that DNA-PKcs could enhance ligation efficiency in different contexts.

We thank the reviewer for the suggestion to mention Liu et al, Nature 2025 in the Discussion. We believe that the conclusions from this study further support our findings and strengthen the view that DNA-PKcs plays a more critical role in NHEJ than suggested by previous studies. To address this, we have added the following discussion at line 362~368:
Consistent with this finding, a recent study by Liu et al. also showed that DNA-PKcs enhances NHEJ activity across DNA ends with different end modifications. The positive role of DNA-PKcs in end-bridging and gap-filling under DNA ends that are not directly ligatable was also highlighted by in vitro ligation assay in that study. Together, these newly uncovered roles of DNA-PKcs in DNA ends with limited accessibility from either chromatinized or modified DNA ends may help reconcile the discrepancies between previous in vivo/ex vivo and in vitro observations.

Reviewer #2 (Remarks to the Author):

This manuscript by Lu et al., determines the structural basis of early NHEJ in the context of the nucleosome. The authors determine a series of cryo-EM structures of Ku70/80, DNA PKcs, DNA PK, bound to nucleosomes that represent a terminal DNA double-strand break. These structures identify how these NHEJ assemblies “unwrap” nucleosomal DNA to engage the terminal double strand break within the nucleosome, and suggest that DNA PKcs may help partially overcome the nucleosome barrier to NHEJ. The authors also determine an initial low-resolution negative stain EM structure of the LR-complex bridging nucleosomes, providing an initial glimpse into how long-range synapse would occur within chromatin. Ultimately, the authors establish a model of NHEJ within the nucleosome that is generally supported by the structural data, though additional biochemical validation would be useful. These structural insights are timely, and compliments similar work recently published in Nature Communications (PMID: 41444611). While this work will assuredly be well-received in the DNA repair field, I do have several comments/concerns that should be considered and address prior to publication in Nature Communications.

We thank the reviewer for their candid assessment of our work. We have carefully read their concerns and have used our revisions to clarify the issues they raise regarding our interpretations and conclusions, which has substantially strengthened our manuscript. We note that Hall et al., 2025 was published after our manuscript was submitted, and in our revisions, we have incorporated comparisons of findings between our study and Hall et al. We have made changes to the text to highlight the complementarity of all of these findings. We appreciate the reviewer’s input and believe our work has improved in addressing their concerns.

1. From the enzymatic assays in Fig. 1a, the authors conclude that the nucleosome can impede NHEJ-mediated ligation by serving as a barrier for Ku which prevents complex assembly. While these data clearly show that the nucleosome impedes NHEJ-mediated ligation, these data do not directly show that this is due to impaired assembly of the rest of the NHEJ complex. In addition, it’s unclear whether these experiments are representative of multiple independent replicate experiments? If so, quantification of the data in Fig. 1a would be helpful to compare the differences between the different reaction conditions.

We thank the reviewer for raising this point. We agree that the enzymatic assays itself cannot rule out the possibility that impaired assembly of the rest of the NHEJ complex impedes ligation on nucleosomal substrate. To validate that nucleosome presents as a barrier for Ku, we used negative staining EM to demonstrate that Ku has a much lower accessibility to a N0 nucleosome than to a N7 nucleosome (Supplementary Fig. 1).

In addition, we added the quantification of triplicates to the enzymatic assays. In Ku-mediated ligation, we quantified the ligation activity by calculating nucleosome-Y30 ligation relative to the Y30 internal ligation. The quantification is included in Fig. 1b.

2. The structures of Ku70 bound to the N7 nucleosome suggest that Ku binding results in unwrapping of the nucleosomal DNA. The authors speculate that the unwrapped nucleosomal DNA is then stabilized by direct interactions between Ku70 and the exposed H2A/H3. This finding is quite interesting. However, it's also possible that Ku70 does not outcompete the histone octamer for the nucleosomal DNA, but rather Ku70 captures the nucleosomal DNA upon spontaneous unwrapping from the histone octamer (PMID: 15580276). The authors may be able to address these two possibilities by determining whether any of the residues in the Ku70 histone binding interface are important for nucleosome binding. Alternatively, I would suggest the authors soften the language in Lines 123-125 and note to the reader the possibility that Ku70 may just capture the nucleosomal DNA upon spontaneous unwrapping.

We agree with the reviewer on the comment that Ku70 may just capture the nucleosomal DNA upon spontaneous unwrapping. To further study the interaction between Ku70 and histone, we mutated Ku by deleting the helix 521-528 for two reasons: 1) In the N7-Ku, N7-DNA-PK, and N20-DNA-PK structures, Ku70 helix 521-528 is observed with the most contact to histone; 2) Ku70 helix 521-528 is located outside of the dimerization domain so that the mutation involving it should not affect the proper folding of the Ku70/80 heterodimer. Shown in Fig. 1f, KuMT shows much lower ligation activity than KuWT on N7 substrate. As a control, KuMT mediates Y30 internal ligation on a similar level to KuWT. The following text corresponding to the experiments is added at line 135-142:

To further examine the role of Ku70-histone interactions in nucleosomal DNA engagement, we performed mutagenesis analysis. We generated a Ku70 mutant lacking residues 521-528, which correspond to one of the helices within the Ku70 histone-bridge region. We then compared the enzymatic activity of NHEJ reactions containing wild-type Ku70/80 (KuWT) or the Ku70 Δ521-528 mutant (KuMT). The KuMT-containing reaction displayed only approximately one-seventh of the ligation efficiency observed with KuWT (Fig. 1f), further suggesting that the Ku70 histone-bridge region plays an important role in supporting NHEJ-mediated ligation on nucleosomal substrates.

We thank the reviewer for bringing up the possibility that Ku70 may just capture the nucleosomal DNA upon spontaneous unwrapping. To address this, we added more analysis in the later section at line 296-307:

Therefore, DNA-PK assembly on nucleosomal DNA ends likely depends on spontaneous DNA end breathing at the nucleosome entry/exit site. To further examine the contribution of spontaneous DNA breathing, we replaced the Widom 601 nucleosome-positioning sequence with the 5S rRNA nucleosome-positioning sequence, a native sequence that is reported to have stronger DNA breathing effect and more closely reflects physiological histone-DNA affinity. Using an in vitro ligation assay, we observed no significant difference in ligation efficiency between Widom 601 and 5S rRNA nucleosomes for either N0 or N7 substrates (Supplementary Fig. 13b). In conclusion, the structural and biochemical results we present here suggest that the interaction between nucleosome and Ku/DNA-PK is very dynamic. Nucleosomal DNA is captured by Ku/DNA-PK through spontaneous end breathing and allowed to rewrap around histone octamer whenever histones are exposed without contacting DNA-PK.

We agree that Ku (and DNA-PK) is just capturing spontaneously unwrapped nucleosomal DNA, and the additional data we present here shows that the histone-DNA interaction is able to counter Ku binding without Ku70's interaction with the exposed histones.

3. The enzymatic assays in Fig. 3a do not indicate that DNA-PKcs overcomes the nucleosome barrier to Ku-mediated ligation. These experiments indicate that DNA-PKcs stimulates Ku-mediated ligation, but it does recover the activity back to the activity seen for Ku-mediated ligation of free DNA ends (Y30 self-ligation control), in particular for the N0 nucleosome. Similar to Fig. 1a, it's also unclear whether these experiments were performed in replicate and why the authors chose not to quantify the respective reactions to help with the comparisons.

We agree with the reviewer that the ligation itself is not enough to conclude that DNA-PKcs overcomes the nucleosome barrier to Ku-mediated ligation. Therefore we made the following changes at line 207~209:

These results indicate that DNA-PKcs specifically stimulates NHEJ-mediated ligation on nucleosomal substrates lacking accessible free DNA linkers as Ku itself fails to mediate NHEJ ligation on these substrates.

We also added the quantification of the experiment shown in Fig. 3a with triplicates. The quantification is now shown in Fig. 3b.

4. Lines 189-191: Ku70 did not fail to assemble on the N0 nucleosome, it just formed a complex ~22% of the time (Extended Data Fig. 1b). Moreover, the number of N0 nucleosomes with DNA-PK bound in the cryo-EM processing workflow in Extended Data Fig. 5 is not all that different than the authors report for Ku70 in the negative stain

data found in Extended Data Fig. 1b. It's unclear how the authors come to the stated conclusion.

We agree with the reviewer's concern that the conclusion drawn from the comparison is not accurate. To address this problem, we added the silver stain gel for the complex assembly of N0-Ku and N7-Ku in Supplementary Fig. 1a and made the following change in the text at line 110~117:

However, further examination by NS-EM revealed a clear difference in assembly efficiency between the two substrates. The N7-Ku sample contained 71% assembled complex, whereas the N0-Ku sample contained only 22% assembled complex (Supplementary Fig. 1b-d). Despite their similar SDS-PAGE profiles, the difference observed by NS-EM suggests that Ku does not stably engage the N0 substrate. As a result, N7 supports more stable Ku binding, resulting in a higher proportion of assembled N7-Ku particles in the NS-EM dataset. This difference in complex formation is consistent with the distinct ligation activities observed for the N0 and N7 substrates.

We think that the conclusion is more accurate as Ku does not form a stable complex on N0 substrate with our reconstitution method, which in part explains why Ku fails to mediate nucleosomal ligation on N0.

We also compared our results with the N0-Ku complex reconstituted by Hall et al. and explained why we were not able to reconstitute a stable N0-Ku complex at line 143-152:

A recent study from Hall et al. reported the structure of an N0-Ku complex, suggesting that Ku can bind nucleosomal substrates even in the absence of an available DNA linker (Supplementary Fig. 3b). Notably, Ku does not occupy in its full footprint in that, arguing that Ku does not reach full activity on the N0 substrate (Supplementary Fig. 3a). Given the different linker lengths used in the two studies, it is striking that both nucleosomes are unwrapped to the same position, SHL (-5.5), suggesting that Ku alone cannot outcompete histone-DNA interactions beyond SHL (-5.5) (Supplementary Fig. 3c). Moreover, that study used GraFix for complex reconstitution, in which crosslinking and gradient purification can stabilize transient or weakly associated complexes, explaining why they were able to assemble the complex for cryo-EM while we did not observe stable N0-Ku complex under NS-EM.

5. Lines 192-199: It's unclear how the authors can distinguish between DNA-PK physically dissociating the nucleosomal DNA from the histone octamer in these structures and DNA-Pk simply capturing the nucleosomal DNA that spontaneously unwraps from the histone octamer, as noted above for Ku70. This alternative possibility really should be discussed in the absence of additional experiments proving which of

these two possibilities is correct. This is a common issue throughout the entire manuscript.

We thank the reviewer for raising this point. We agree with the reviewer that evidence is needed to differentiate between DNA-PK physically dissociating DNA and capturing DNA spontaneously unwrapped from the histones. To address this problem, we reconstituted N0 and N7 substrates using the 5s RNA nucleosome positioning sequence, which is a native nucleosome positioning sequence and has a lower affinity than the artificial Widom 601 sequence. By conducting ligation assay, we found that the ligation profile is very similar between Widom 601 and 5s RNA nucleosomes (Supplementary Fig. 3b). In addition, comparison among all of the NHEJ-nucleosome structure (this study and Hall et al. 2025) shows that the nucleosome is only unwrapped to ~ SHL (-5) (Supplementary Fig. 15). This argues that DNA-PK is indeed only capturing the nucleosomal DNA through end breathing effect, as studies have shown that nucleosome breathing effect is strong in the region SHL (-7) ~ (-5). Due to the strong interaction between H2A/H2B and DNA, breathing effect is much weaker beyond SHL (-5). Also, the ligation profile is very similar even on a weaker 5sRNA nucleosome, further suggesting that both Ku and DNA-PK is only capturing the spontaneously breathing nucleosome end. The text corresponding to this part is at line 289-307:

Comparison of the Ku/DNA-PK-nucleosome structures determined in this study with those reported by Hall et al. reveals a notable shared feature: in all structures, nucleosome unwrapping does not extend beyond approximately SHL (-5) (Supplementary Fig. 15). Studies have shown that nucleosome are susceptible to spontaneous breathing especially at the entry/exit site and SHL (-5) has been proposed as a major pause site for proteins that encounter the histone barrier, as observed for RNA polymerase II and the replisome. In these systems, ATP hydrolysis is coupled to active translocation through the nucleosome. By contrast, ATP-dependent DNA translocation has not been reported for the DNA-PK complex. Therefore, DNA-PK assembly on nucleosomal DNA ends likely depends on spontaneous DNA end breathing at the nucleosome entry/exit site. To further examine the contribution of spontaneous DNA breathing, we replaced the Widom 601 nucleosome-positioning sequence with the 5S rRNA nucleosome-positioning sequence, a native sequence that is reported to have stronger DNA breathing effect and more closely reflects physiological histone-DNA affinity. Using an in vitro ligation assay, we observed no significant difference in ligation efficiency between Widom 601 and 5S rRNA nucleosomes for either N0 or N7 substrates (Supplementary Fig. 13b). In conclusion, the structural and biochemical results we present here suggest that the interaction between nucleosome and Ku/DNA-PK is very dynamic. Nucleosomal DNA is captured by Ku/DNA-PK through spontaneous end breathing and allowed to rewrap around histone octamer whenever histones are exposed without contacting DNA-PK.

6. The section entitled “DNA-PK progressively unwraps nucleosome in an ATP-independent way” is extremely difficult to follow as a reader, even for a structural biologist. I would encourage the authors to attempt to clarity this section for readers.

We thank the reviewer for pointing this out. We agree that this section is very confusing. To address this, we re-wrote this section and re-titled it to “DNA-PK captures spontaneously exposed nucleosomal DNA” (line). To sum up, the structural and biochemical results we present in this section suggest that the interaction between nucleosome and Ku/DNA-PK is very dynamic. Nucleosomal DNA is captured by Ku/DNA-PK through spontaneous end breathing and allowed to rewrap around histone octamer whenever histones are exposed without DNA-PK contact.

7. The authors thoroughly describe different aspects of the DNA-PK-nucleosome structures, pointing out several novel contacts between different components of DNA-PK and the nucleosome. However, it’s difficult to interpret whether any of these novel interactions observed are important for NHEJ to occur within the nucleosome without some biochemical validation. Examples include:

• SAP domain - nucleosomal DNA interaction
• FAT domain - H3 interaction
• Ku70 vWA domain – H3 interaction
• Ku80 - H2A loop 68-79 interaction
• Ku70 - H2B loop 44-52 interaction

Given the lack of biochemical validation showing these interactions are important for NHEJ to occur within the nucleosome, the authors really should tone down the interpretation in lines 257-268.

We agree the reviewer’s suggesting for a mutagenesis study. As described in comment #2, a Ku mutant was made based on the structural finding from the N7-Ku complex. By incorporating DNA-PKcs to the ligation assay with KuMT and KuWT, we found that DNA-PKcs has no problem stimulating NHEJ ligation on N7 substrate with both KuMT and KuWT (Supplementary Fig. 13). This indicates that DNA-PKcs can compensate for weakened Ku-histone interactions. We added text in the manuscript at line 272-277 to incorporate this analysis:

We also conducted ligation assays comparing KuWT and KuMT with the addition of DNA-PKcs (Supplementary Fig. 13a). Strikingly, the addition of DNA-PKcs increases ligation efficiency in the KuMT-containing reaction to a level comparable to that observed with KuWT. This rescue highlights the ability of DNA-PKcs to promote NHEJ-

mediated ligation on nucleosomal substrates and suggests that DNA-PKcs can compensate for weakened Ku-histone interactions.

For other potential interactions between DNA-PK and nucleosome, we did not conduct mutagenesis study because limited resolution at the interfaces specified precludes detailing them at a residue specific level, preventing us from confidently assigning targets for mutational studies. Therefore, we toned down the interpretations in the two paragraphs at line 249~277.

…… The density of the FAT domain of DNA-PKcs merges with the H3 N-terminal tail under lower contour threshold, suggesting a potential interaction. ……. The position of this extra density appears at the first major groove of the exiting DNA from Ku70/80, consistent with the Ku70 SAP resolved in the previous structure. Together, these observations suggest potential interactions that might trap DNA-PK in its intermediate state on the N7 substrate. ……. Moreover, in addition to the previously described K70 histone bridge, Ku80 also contributes to potential histone-binding interface ……

8. The manuscript ends with an attempt to generate an LR-complex bound to the nucleosome to describe how synapsis may occur within chromatin. However, the authors only assess the complex by negative stain EM, showing that DNA-PK can can bridge broken nucleosomal DNA ends. Can the authors comment on why they decided to forego collecting cryo-EM datasets on the LR-complex to obtain higher resolution insight? This structure could add some interesting insight to the manuscript, though I do appreciate the work involved in collecting additional structures.

We thank the reviewer for the suggestion to collect cryo-EM datasets of the LR–nucleosome complex. We agree that a cryo-EM structure could provide additional mechanistic insights and further enrich the manuscript. However, we encountered substantial difficulties in enriching and stabilizing the complex for high-resolution cryo-EM analysis using both bead-based pull-down and glycerol gradient approaches (Supplementary Fig. 16a). As a result, we were only able to assess the complex by negative-stain EM.

We believe that the negative-stain EM data, together with the ligation assays, already provide sufficient evidence to support DNA-PK's ability to stimulate NHEJ-mediated ligation on nucleosomal substrates. Based on the negative-stain EM analysis, a cryo-EM study would most likely provide additional structural details regarding nucleosome repositioning within the LR complex. While such findings would certainly be interesting, we feel they would extend beyond the main scope of the current manuscript, which is focused on establishing the functional and structural role of DNA-PK during the initiation of NHEJ on nucleosomal DNA.

9. I strongly encourage the authors to reference work from Amanda Chaplin's Lab on NHEJ within nucleosomes that was recently published in Nature Communications. It seems as if the structures within this manuscript and the structures from the Chaplin Lab have some notable similarities and differences. A few sentences pointing these out in the discussion, and how they fit into the authors model of NHEJ within chromatin would be very helpful for readers.

We thank the reviewer for the suggestion to include a comparison between our study and Hall et al., 2025, which was published after our manuscript was originally submitted. To address the similarities and differences between the two studies, we have made the following changes in several paragraphs and figures:

Line 143~152:

A recent study from Hall et al. reported the structure of an N0-Ku complex, suggesting that Ku can bind nucleosomal substrates even in the absence of an available DNA linker (Supplementary Fig. 3b). Notably, Ku does not occupy in its full footprint in that, arguing that Ku does not reach full activity on the N0 substrate (Supplementary Fig. 3a). Given the different linker lengths used in the two studies, it is striking that both nucleosomes are unwrapped to the same position, SHL (-5.5), suggesting that Ku alone cannot outcompete histone-DNA interactions beyond SHL (-5.5) (Supplementary Fig. 3c). Moreover, that study used GraFix for complex reconstitution, in which crosslinking and gradient purification can stabilize transient or weakly associated complexes, explaining why they were able to assemble the complex for cryo-EM while we did not observe stable N0-Ku complex under NS-EM.

Line 218~220:

A similar state was reported by Hall et al. in the recent study of DNA-PK assembled on an N3 substrate, where DNA-PKcs likewise exhibited only a 5 bp footprint at the DNA end (Supplementary Fig. 7c, d).

Line 289~291:

Comparison of the Ku/DNA-PK-nucleosome structures determined in this study with those reported by Hall et al. reveals a notable shared feature: in all structures, nucleosome unwrapping does not extend beyond approximately SHL (-5) (Supplementary Fig. 15).

Minor Comments

1. Lines 67-69: While this is generally a true statement, some chromatin remodeling enzymes involved in the DNA damage response have also been shown to remodel nucleosomes away from DNA breaks (PMID: 38307862).

We thank the reviewer for pointing this out and we have toned down our statement in line 67~69.

In addition, some chromatin-remodeling complexes directly related to DNA-damage response have also been shown to slide the histone octamer to the DNA ends, potentially favoring the formation of short DNA linkers.

2. Lines 95-99: This comparison is valid. However, nucleosome-mediated reduction in the enzymatic activities of multiple DNA repair enzymes within the BER and NER pathways have also been described, suggesting this is not an NHEJ-specific phenomenon. This may be worth pointing out.

We thank the reviewer for this suggestion and have incorporated it into line 99.

Notably, this nucleosome-related reduction in activity resembles observation from in vitro studies of other DNA-repair pathways, such as NER and BER.

3. Lines 103-104: The N7 nucleosome was not previously introduced before this point in the manuscript, only the N0 and N-12. It would be nice to introduce that this nucleosome has a 7 bp linker so that the reader doesn't need to go to the oligonucleotide table to find that information.

We thank the reviewer for this suggestion and have incorporated it into line 105.

…… we modified the N7 (nucleosome substrate with free DNA linker of 7 bp) ……

4. Line 126: The idea of Ku70 as an ATP-independent translocase comes out of nowhere. I'm unsure what this is attempting to convey, but I would suggest the authors try to rephrase this in a way that better described this part of the conclusion.

We agree with the reviewer's comment and have removed the idea of ATP-independent translocase from the manuscript.

5. Lines 138-140: This description is a bit confusing as currently written. The authors may want to use the number of bps and the SHL locations to denote where Ku70 is bound vs. where DNA-PKc is bound in relation to the nucleosome to improve clarity for the reader.

We thank the reviewer for pointing this out. To address this, we altered the text in line 163~165 by counting from the DNA termini inward along the DNA duplex. We hope this will clarify the confusion.

In the structure, DNA-PKcs binds to 11 bp along the linker while Ku occupies to the next 14 bp relative to the nucleosome (Fig. 2c)

6. Lines 146-147: remove the second “binding” from this sentence.

We thank the reviewer for pointing this out and we have adjusted the text accordingly.

7. Line 324: chromatin instead of chromatins

We thank the reviewer for pointing this out and we have adjusted the text accordingly.

8. Lines 504-509: If the cryo-EM maps and models have already been deposited into the EMDB and PDB, respectively, the authors should ensure they edit in the correct EMDB and PDB accession numbers within the “Data and Materials Availability” section of the manuscript.

We thank the reviewer for pointing this out and we have added the number into the form.

9. Figure 4: Histone H2B DNA binding loop is misspelled.

We thank the reviewer for pointing this out and we have adjusted the text accordingly.

Reviewer #3 (Remarks to the Author):

Research on the NHEJ pathway, an essential DNA damage repair mechanism, has been conducted primarily using naked DNA. However, because eukaryotic DNA is packaged into chromatin, understanding how NHEJ proceeds on chromatin substrates is highly significant. In this manuscript, the authors use cryo-EM structural analysis to investigate how the key NHEJ factors Ku70/80 and DNA-PKcs interact with the nucleosome. Notably, they focus on how much DNA is peeled away from the nucleosome and propose that the presence of DNA-PKcs promotes this peeling to secure the DNA substrate required for its activity. Their identification of multiple intermediate states during DNA peeling, and the suggestion that DNA-PK rotates around the nucleosome while peeling the DNA, represent novel aspects of the study. On the other hand, structures of the nucleosome–Ku70/80–DNA-PK complex have already been reported, including work showing DNA-PK–dependent changes in nucleosome structure. The manuscript does not cite and compare with the prior work (Hall et al., Nat Commun., 2025).
Further concerns are detailed below.

We thank the reviewer for their careful reading and thoughtful critique of our manuscript. Responding to their comments and incorporating their suggestions has led to a substantial improvement of our manuscript. We acknowledge the concerns the reviewers raise regarding the novelty of our investigation. We note that Hall et al., 2025 was published after our manuscript was submitted. By comparing the two works, we have found that the structures are actually complementing in these two studies. We also agree with the reviewer that our statement in 'DNA-PK unwraps nucleosome in an ATP-independent way' is not accurate, and based on the additional data, we have changed it into 'DNA-PK captures spontaneous nucleosome end breathing'. Again, we are appreciative of the reviewer's thorough comments and believe that our manuscript has been strengthened by addressing their concerns.

Major Comments

1. The authors should further discuss the physiological significance of the reported structures. In vivo, chromatin remodelers are activated at DSB sites, and several studies suggest that chromatin disassembly occurs prior to Ku70/80 recruitment (Tripuraneni et al., Genome Res, 2021; Li & Tyler, Elife, 2016). While the authors cite work showing nucleosomes sliding toward DNA ends, those same studies observe subsequent ejection. Thus, it remains unclear whether a nucleosome is stably positioned immediately adjacent to a DSB during NHEJ. Clarifying the conditions under which these nucleosome-bound intermediates might exist in vivo would help readers better understand the physiological context of these structures.

We thank the reviewer for the comments on the physiological context of nucleosome-DNA-PK complexes. We agree that the condition is not clarified in the text. To address this, we made following changes to the texts at line 64-72:

As the first sensors of DSBs in mammalian cells, Ku and DNA-PKcs are recruited immediately upon break formation, and its assembly can facilitate downstream histone modifications and local chromatin reorganization. In addition, some chromatin-remodeling complexes directly related to DNA-damage response have also been shown to slide the histone octamer to the DNA ends, favoring the formation of short DNA linkers. Therefore, understanding how Ku and DNA-PKcs engage chromatinized DNA ends under such constrained conditions, where only minimal free duplex DNA is available, is critical for defining the molecular basis of NHEJ within chromatin.

With regard to the studies mentioned (Tripuraneni et al., Genome Res, 2021; Li & Tyler, Elife, 2016), we would like to acknowledge several important considerations. In Tripuraneni et al., Genome Res, 2021, the experiments were performed in Saccharomyces cerevisiae, which lacks a homolog of DNA-PKcs, and YKu70/80 itself does not possess any kinase activity In addition, comparison of human and yeast proteomic data from PaxDB suggests that Ku is present at substantially higher relative abundance in human cells than in yeast. More broadly, yeast relies less heavily on NHEJ as a major DNA double-strand break repair pathway compared with human cells. Given these biological differences, we believe that the sequence of Ku binding and nucleosome disassembly may not be directly comparable between yeast and human systems.

In Li & Tyler, Elife, 2016, they used Ku80-knockdown HEK293T cells and observed local nucleosome disassembly, demonstrating that Ku80 is not required for nucleosome disassembly. However, this does not necessarily establish that nucleosome disassembly occurs prior to the binding of NHEJ factors. We agree that an important question is whether a nucleosome remains stably positioned immediately adjacent to a DSB during NHEJ. Notably, one study has shown that DNA-PK activity promotes chromatin decondensation (PMID: 31396623), supporting the idea that DNA-PK can actively influence local chromatin structure. Therefore, we consider a DNA-PK-bound nucleosome to be a physiologically relevant intermediate during the initiation of NHEJ in vivo.

2. It would also be helpful to clarify whether the nucleosome “unwrapping” seen in the structures is actively induced by DNA-PK, or if it is a passive outcome of DNA-PK’s high DNA affinity. However, nucleosomal DNA ends are inherently prone to spontaneous breathing and partial unwrapping. The complexes might simply capture transiently exposed DNA, rather than actively peeling DNA from the histone core, as suggested in

reference (40). Biochemical experiments comparing the state of the nucleosome before and after DNA-PK binding would help clarify this point and would further strengthen the conclusions of the study.

We thank the reviewer for pointing out that DNA-PK might not actively unwrap nucleosome but rather cause unwrapping due to its high DNA affinity and weak local DNA-histone interaction. After carefully reviewing literatures and the additional data, we have altered our conclusion to DNA-PK capture nucleosomal DNA through spontaneous end breathing and out compete weak DNA-histone interactions in SHL (-7) to SHL (-5). Combining with the ligation assay using a 5sRNA nucleosome instead of widom 601 nucleosome, we made the following changes in the text at line 289-307:

Comparison of the Ku/DNA-PK-nucleosome structures determined in this study with those reported by Hall et al. reveals a notable shared feature: in all structures, nucleosome unwrapping does not extend beyond approximately SHL (-5) (Supplementary Fig. 15). Studies have shown that nucleosome are susceptible to spontaneous breathing especially at the entry/exit site and SHL (-5) has been proposed as a major pause site for proteins that encounter the histone barrier, as observed for RNA polymerase II and the replisome. In these systems, ATP hydrolysis is coupled to active translocation through the nucleosome. By contrast, ATP-dependent DNA translocation has not been reported for the DNA-PK complex. Therefore, DNA-PK assembly on nucleosomal DNA ends likely depends on spontaneous DNA end breathing at the nucleosome entry/exit site. To further examine the contribution of spontaneous DNA breathing, we replaced the Widom 601 nucleosome-positioning sequence with the 5S rRNA nucleosome-positioning sequence, a native sequence that is reported to have stronger DNA breathing effect and more closely reflects physiological histone-DNA affinity. Using an in vitro ligation assay, we observed no significant difference in ligation efficiency between Widom 601 and 5S rRNA nucleosomes for either N0 or N7 substrates (Supplementary Fig. 13b). In conclusion, the structural and biochemical results we present here suggest that the interaction between nucleosome and Ku/DNA-PK is very dynamic. Nucleosomal DNA is captured by Ku/DNA-PK through spontaneous end breathing and allowed to rewrap around histone octamer whenever histones are exposed without contacting DNA-PK.

3. The authors propose direct interactions between the nucleosome, Ku70/80, and DNA-PKcs based on a composite map. However, these contact regions lie at the boundaries of separately refined maps where the resolution gap is big. Consequently, the evidence for these direct contacts is weak, and the map in several areas appears insufficient to define specific amino acid interactions confidently. To substantiate these

claims, the authors should provide further evidence, such as ligation assays using mutant proteins designed to disrupt these interfaces.

We thank the reviewer for the comments on the interactions observed between DNA-PK and histones. We agree that the map quality for some of the interactions described is not sufficient to define these interactions. To address this problem, we deleted the Ku70 helix 521-528 and conducted the ligation assay with the mutant Ku. The reasons we chose this helix to mutate are that 1) In the N7-Ku, N7-DNA-PK, and N20-DNA-PK structures, Ku70 helix 521-528 is observed with the most contact to histone; 2) Ku70 helix 521-528 is located outside of the dimerization domain so that the mutation involving it should not affect the proper folding of the Ku70/80 heterodimer.

By conducting in vitro ligation using mutated Ku, as shown in Fig. 1f and Supplementary Fig. 13a, we found that KuMT shows much lower ligation activity than KuWT on N7 substrate. As a control, KuMT mediates Y30 internal ligation on a similar level to KuWT. This indicates that histone-DNA interaction is able to counter Ku binding without Ku70's interaction with the exposed histones. We added text in the manuscript at line 135-142 and line 272-277 to incorporate this analysis:

To further examine the role of Ku70-histone interactions in nucleosomal DNA engagement, we performed mutagenesis analysis. We generated a Ku70 mutant lacking residues 521-528, which correspond to one of the helices within the Ku70 histone-bridge region. We then compared the enzymatic activity of NHEJ reactions containing wild-type Ku70/80 (KuWT) or the Ku70 Δ521-528 mutant (KuMT). The KuMT-containing reaction displayed only approximately one-seventh of the ligation efficiency observed with KuWT (Fig. 1f), further suggesting that the Ku70 histone-bridge region plays an important role in supporting NHEJ-mediated ligation on nucleosomal substrates.

We also conducted ligation assays comparing KuWT and KuMT with the addition of DNA-PKcs (Supplementary Fig. 13a). Strikingly, the addition of DNA-PKcs increases ligation efficiency in the KuMT-containing reaction to a level comparable to that observed with KuWT. This rescue highlights the ability of DNA-PKcs to promote NHEJ-mediated ligation on nucleosomal substrates and suggests that DNA-PKcs can compensate for weakened Ku-histone interactions.

For other potential interactions between DNA-PK and nucleosome, we did not conduct mutagenesis study because limited resolution at the interfaces specified precludes detailing them at a residue specific level, preventing us from confidently assigning targets for mutational studies. Moreover, given the complexity of the system, a null result of a mutagenesis experiment would not invalidate the presence of these contacts.

Therefore, we decided to not conduct mutagenesis on these potential interactions and toned down the interpretations in the two paragraphs at line 249~277.

…… The density of the FAT domain of DNA-PKcs merges with the H3 N-terminal tail under lower contour threshold, suggesting a potential interaction. ……. The position of this extra density appears at the first major groove of the exiting DNA from Ku70/80, consistent with the Ku70 SAP resolved in the previous structure. Together, these observations suggest potential interactions that might trap DNA-PK in its intermediate state on the N7 substrate. ……. Moreover, in addition to the previously described K70 histone bridge, Ku80 also contributes to potential histone-binding interface ……

4. Further details regarding sample quality are required. Since the state of the DNA ends is central to this study, the authors must provide data (e.g., gel images) showing the purity of the nucleosome preparations and the proportion of naked DNA and hexasomes. In addition, it is unclear how "state 1" and "state 2" samples were prepared for structural analysis, making it difficult to determine the origin of the structural differences observed between the states. The intermediate steps of the GraFix procedure, or any other preparative steps, should also be presented to allow the reader to assess the sample heterogeneity and the validity of the structural conclusions.

We agree with the reviewer that more data is needed to show the results of the complex assembly. We have added Supplementary Fig. 17 and 18 to show the quality of the nucleosome with <5% free DNA, the pull-down assembly of nucleosome-DNA-PK complex, and the NS-EM assessment of complex assembly quality. We have also added Supplementary Fig. 1a and 16a for the assessment of N7-Ku and N7-LR assembly.

We have also clarified the processes of reconstituting N7-DNA-PK state 1 and state 2. In brief, N7-DNA-PK state 1 is adding DNA-PKcs after the elution of N7-Ku for 1 min while N7-DNA-PK state 2 has DNA-PKcs reacting in the tube for 15 min with N7-Ku before elution.

The following text has been added to the manuscript to clarify the sample preparation:

Line 220-222:

To visualize DNA-PK assembly on the N7 substrate, we collected two cryo-EM datasets on N7-DNA-PK complex at different reaction times (1 minute and 15 minutes) (Fig. 3c, Supplementary Fig. 8, 9).

Line 423-436 in Methods:

For N7-DNA-PK (state 1), 2 μL of each nucleosomal substrates were similarly incubated with T1 Streptavidin Magnetic Beads for 15 min in 10 μL assembly buffer, followed by

addition of Ku at a 1:1 molar ratio and incubation for 5 min. After three washes, complexes were eluted with 3 μL assembly buffer containing 2% EcoRI. The eluted Ku-nucleosome complexes were then incubated with DNA-PKcs for 1 min before cross-linking, using a concentration determined by negative-stain EM to achieve an approximate 1:1 molar ratio between Ku-bound nucleosome and free DNA-PKcs.

For N0-DNA-PK, N20-DNA-PK, N7-DNA-PK (state 2) complexes, 2 μL of the desthio-biotinylated nucleosomal substrate was incubated with T1 Streptavidin Magnetic Beads for 15 min in 10 μL assembly buffer, followed by sequential addition of Ku and DNA-PKcs at 1:1 molar ratios, for 5 min and 15 min, respectively. After three washes, the assembled complex was eluted using 3 μL assembly buffer supplemented with 1 mM biotin. Elution was then cross-linked with 0.0625% glutaraldehyde for 5 min on ice before EM analysis.

As shown in Supplementary Fig. 18, it would be hard to assess heterogeneity directly from NS-EM analysis due to the flexibility of nucleosome in the complex. It is hard to distinguish between different views of nucleosome based on the 2D class averages. Therefore, we only used NS-EM as a confirmation for the complex assembly and relied on cryo-EM for actual quality assessment.

5. The authors state that they did not build a model for the N0–DNA-PK complex due to limited resolution (L187–189), yet a model appears to be shown in the figures. Given the limited resolution, as the authors themselves mention, it may be challenging to confidently determine the nucleosome orientation.

We agree with the reviewer that the section corresponding to N0-DNA-PK can be confusing. We made the following changes in the text at line 212~215 and figures (Supplementary Fig. 7) to further explain:

Due to the limited local resolution of the nucleosome density, the nucleosome could only be modeled by rigid-body fitting, whereas the higher-resolution DNA-PK region allowed more detailed model building, particularly at the DNA end within the DNA-PKcs cradle (Supplementary Fig. 7b).

Although the resolution of the nucleosome was low, the overall shape of the histone octamer is preserved, in particular at H2A-H2B dimer. We relied on the histone octamer shape and the DNA termini to rigid-body-fit a nucleosome model into the map.

6. The authors show that DNA-PK induces nucleosome unwrapping in an ATP-independent manner. The authors should clarify precisely where the required energy originates. I also encourage the authors to estimate the energy required for nucleosome unwrapping and to compare it quantitatively with the proposed energy source.

We thank the reviewer for pointing this out. After carefully reviewing literatures and the additional data, we have changed our model to DNA-PK capture the spontaneously unwrapped nucleosomal DNA end. Therefore, we made substantial change in the section "DNA-PK captures spontaneously exposed nucleosomal DNA" (original title: "DNA-PK progressively unwraps nucleosome in an ATP-independent way"). Major change includes a re-write of two paragraphs with additional analysis at line 289-307:

Comparison of the Ku/DNA-PK-nucleosome structures determined in this study with those reported by Hall et al. reveals a notable shared feature: in all structures, nucleosome unwrapping does not extend beyond approximately SHL (-5) (Supplementary Fig. 15). Studies have shown that nucleosome are susceptible to spontaneous breathing especially at the entry/exit site and SHL (-5) has been proposed as a major pause site for proteins that encounter the histone barrier, as observed for RNA polymerase II and the replisome. In these systems, ATP hydrolysis is coupled to active translocation through the nucleosome. By contrast, ATP-dependent DNA translocation has not been reported for the DNA-PK complex. Therefore, DNA-PK assembly on nucleosomal DNA ends likely depends on spontaneous DNA end breathing at the nucleosome entry/exit site. To further examine the contribution of spontaneous DNA breathing, we replaced the Widom 601 nucleosome-positioning sequence with the 5S rRNA nucleosome-positioning sequence, a native sequence that is reported to have stronger DNA breathing effect and more closely reflects physiological histone-DNA affinity. Using an in vitro ligation assay, we observed no significant difference in ligation efficiency between Widom 601 and 5S rRNA nucleosomes for either N0 or N7 substrates (Supplementary Fig. 13b). In conclusion, the structural and biochemical results we present here suggest that the interaction between nucleosome and Ku/DNA-PK is very dynamic. Nucleosomal DNA is captured by Ku/DNA-PK through spontaneous end breathing and allowed to rewrap around histone octamer whenever histones are exposed without contacting DNA-PK.

7. The authors use the 601 sequence for nucleosome assembly. However, the 601 sequence forms relatively rigid nucleosomes, and the total stability of these nucleosomes is higher than that of native genomic sequences. I recommend using nucleosomes assembled with native DNA sequences (e.g., α-satellite or LIN28) to investigate how DNA-PK and Ku overcome these nucleosomal barriers.

We really thank the reviewer for suggesting the experiment of replacing Widom 601 sequence to a more native nucleosome sequence. To address this, we reconstituted nucleosome using 5s RNA nucleosome positioning sequence, which is a native nucleosome sequence and has weaker binding affinity toward histone octamers. By conducting ligation assay on N0 and N7 nucleosomes using these two sequences, we found that the ligation profile is very similar between Widom 601 and 5s RNA

nucleosomes (Supplementary Fig. 3b). In addition, comparison among all of the NHEJ-nucleosome structure (this study and Hall et al. 2025) shows that the nucleosome is only unwrapped to ~ SHL (-5) (Supplementary Fig. 15). This argues that DNA-PK is indeed only capturing the nucleosomal DNA through end breathing effect, as studies have shown that nucleosome breathing effect is strong in the region SHL (-7) ~ (-5). Due to the strong interaction between H2A/H2B and DNA, breathing effect is much weaker beyond SHL (-5). Also, the ligation profile is very similar even on a weaker 5sRNA nucleosome, further suggesting that both Ku and DNA-PK are only capturing the spontaneously breathing nucleosome end.

The analysis is incorporated in the manuscript at line 298-307:

To further examine the contribution of spontaneous DNA breathing, we replaced the Widom 601 nucleosome-positioning sequence with the 5S rRNA nucleosome-positioning sequence, a native sequence that is reported to have stronger DNA breathing effect and more closely reflects physiological histone-DNA affinity. Using an in vitro ligation assay, we observed no significant difference in ligation efficiency between Widom 601 and 5S rRNA nucleosomes for either N0 or N7 substrates (Supplementary Fig. 13b). In conclusion, the structural and biochemical results we present here suggest that the interaction between nucleosome and Ku/DNA-PK is very dynamic. Nucleosomal DNA is captured by Ku/DNA-PK through spontaneous end breathing and allowed to rewrap around histone octamer whenever histones are exposed without contacting DNA-PK.

8. In Figure 5, steps 5 and 6 appear to be speculative, as no direct evidence supporting this model is presented. Although LR complex assembly has been demonstrated using naked DNA in previous studies, the authors have not provided data supporting the proposed model in the nucleosomal context. Additional experimental evidence would be necessary to substantiate this model.

We thank the reviewer for pointing this out. We agree that step 5 and step 6 are speculative. We attempted to obtain cryo-EM structures of these complexes but suffered from low complex assembly efficiency, as suggested in Supplementary Fig. 16, where we used NS-EM to study N7-LR complex. The speculation is based on the ligation assay as all the NHEJ factors associated with synapsis formation were provided and ligation was successfully induced. To clarify step 5 and step 6 in the model, we rephrased its description in the discussion section at line 339-341:

Step 5 and 6: hypothetical models of DNA-PK achieving LR and SR synapsis and permitting NHEJ to carry out repair (Fig. 5, step 5 and 6).

Minor Comments

1. In all structural analyses, information on local resolution and angular distribution is not provided.

We thank the reviewer for pointing this out. To address this, we have added the angular distribution to each cryo-EM data processing figure and a Supplementary Fig.19 to show the local resolution of each map.

2. In Extended Data Fig. 1A, 11A, scale bars are missing.

We thank the reviewer for pointing this out. We have added scale bars to each NS-EM micrograph.

3. In Fig. 4a and 4b, the red arrows are hard to distinguish, making it unclear which regions are highlighted. Using clearer colors or zoomed-in views would improve visibility.

We thank the reviewer for pointing this out. We have adjusted the direction of the arrow and the position of the annotation to make the graph clearer.

4. In Figure 4c, the current H3 tail model appears to be placed in density corresponding to the DNA end–DNA-PK interface. H3 tail density should instead emerge from between the two DNA gyres.

We thank the reviewer for pointing this out. The corrected PDB is attached to the response.

5. In Figure 4e, although the map shows interactions between Ku70–Ku80 and Ku80–H2A/H2B, the direct interaction between Ku70 and H2A/H2B is not visible even at higher contour levels. Clearer map evidence should be provided to support this interaction.

We thank the reviewer for pointing this out. We have added Supplementary Fig. 12 to show the map densities.

6. In Extended Data Fig. 9a, the threshold values should be indicated for reproducibility. In addition, some density is also present in state 2 at lower contour levels; therefore, the

authors should compare map with map rather than map with model. It is also unclear where the model shown originates, as it is not included among the provided PDB files.

We thank the reviewer for pointing this out. After adjusting the content of the section, we decided to remove this panel because it is not relevant to the analysis anymore and would introduce extra confusion.

7. In Extended Data Fig. 10b, the panel is difficult to interpret. The enlarged region should be clearly marked on the full map, and DNA density should be color-coded for clarity. In addition, because the composite map was generated by combining three states, the matching DNA length is expected and should be acknowledged.

We thank the reviewer for pointing this out. We have adjusted Supplementary Fig.14 to make it easier to read and added more information in panel b for clarity.

8. In Supplementary Table 2, the model resolution is reported using the FSC 0.143 threshold, but it should be estimated using the 0.5 criterion.

We thank the reviewer for pointing this out. We have adjusted the resolution reported in Table 2 by using FSC 0.5.

Author's Response

Reviewer #1 (Remarks to the Author):

The authors have addressed my major concerns - after some remaining minor points are addressed, the manuscript is suitable for publication in my opinion.

Minor points:

1. including the 5S RNA sequence is a good addition. However, writing in the text that there is 'no significant difference' between 601 and 5S nucleosomes is not justified without a statistical test for significance, which has not been performed as far as I can see.

We thank the reviewer for bringing up this point. To address this, we toned down the conclusion in the manuscript to 'a similar pattern in ligation efficiency' at line 298.

2. The quantifications are an improvement. However, as above no stats have been performed to test for statistically significant differences between the relevant samples. These should be included prior to publication

We thank the reviewer for bringing this up. We have performed statistical tests and reported P values for all quantified analyses.

Other than that, I commend the authors on a nice study.

Reviewer #2 (Remarks to the Author):

The authors have thoroughly addressed my original comments/concerns. This manuscript will be extremely valuable to the DNA repair field, and I completely support the publication of this manuscript in Nature Communications.

Minor Comment

1. Lines 569-574: It appears the PDB and EMDB accession codes are still missing from the revised manuscript.

We thank the reviewer for pointing this out. We have added all the PDB and EMDB accession codes in the manuscript.

Reviewer #3 (Remarks to the Author):

The authors have addressed the previous comments well, and the manuscript has been substantially improved. In particular, the comparison with the recent study by Hall et al. is well presented, and the identification of multiple intermediate states during DNA-PK assembly on the nucleosome further highlights the novelty of this work. The additional biochemical experiments also strengthen the authors' conclusions.
The remaining comments are minor:
1. Referring to the Ku70 Δ521–528 mutant as "KuMT" is confusing because it is easily mistaken for "KuWT" and does not indicate that the mutation is in Ku70. A more descriptive name, such as "Ku70 Δ521–528", would improve clarity.

We thank the reviewer for pointing this out. We agree with the reviewer and have changed the abbreviation accordingly.

2. Several expressions, including "Advancement of DNA-PKcs" in Fig. 5b and terms such as "DNA-PK unwraps," "DNA-PKcs advances," and "translocates" throughout the manuscript, still imply that DNA-PK actively peels DNA away from the histone octamer. The wording should be revised to better reflect the current model.

We thank the reviewer for pointing this out. We agree with the reviewer that 'Advancement' does not describe the current model accurately as it suggests active movement with energy accompanied. But we think that 'unwrap' and 'translocate' are neutral description of what happens at the ending state of Ku/DNA-PK binding and do not necessarily indicate an active peeling of the DNA. Therefore, we replaced the term 'Advancement of DNA-PKcs' in Fig. 5b to 'Initial translocation of DNA-PKcs'.

3. The statement "DNA-PKcs can compensate for weakened Ku-histone interactions" (lines 276–277) appears stronger than the data support and should be replaced with a more conservative statement, such as "DNA-PKcs rescues the ligation defect."

We thank the reviewer for pointing this out. To address this, we changed the statement to 'This further highlights the ability of DNA-PKcs to rescue ligation defect on nucleosomal substrates.' at line 272 and 273.
